# Genomic Features of the Damselfly *Calopteryx splendens* Representing a Sister Clade to Most Insect Orders

**DOI:** 10.1093/gbe/evx006

**Published:** 2017-01-30

**Authors:** Panagiotis Ioannidis, Felipe A. Simao, Robert M. Waterhouse, Mosè Manni, Mathieu Seppey, Hugh M. Robertson, Bernhard Misof, Oliver Niehuis, Evgeny M. Zdobnov

**Affiliations:** 1 Department of Genetic Medicine and Development, University of Geneva Medical School, Geneva, Switzerland; 2 Swiss Institute of Bioinformatics, Geneva, Switzerland; 3 Department of Entomology, University of Illinois at Urbana-Champaign, Urbana, IL; 4 Center for Molecular Biodiversity Research, Zoological Research Museum Alexander Koenig, Bonn, Germany

**Keywords:** winged insect, banded demoiselle, damselfly, Palaeoptera, Odonata, whole genome sequencing

## Abstract

Insects comprise the most diverse and successful animal group with over one million described species that are found in almost every terrestrial and limnic habitat, with many being used as important models in genetics, ecology, and evolutionary research. Genome sequencing projects have greatly expanded the sampling of species from many insect orders, but genomic resources for species of certain insect lineages have remained relatively limited to date. To address this paucity, we sequenced the genome of the banded demoiselle, *Calopteryx splendens*, a damselfly (Odonata: Zygoptera) belonging to Palaeoptera, the clade containing the first winged insects. The 1.6 Gbp *C. splendens* draft genome assembly is one of the largest insect genomes sequenced to date and encodes a predicted set of 22,523 protein-coding genes. Comparative genomic analyses with other sequenced insects identified a relatively small repertoire of *C. splendens* detoxification genes, which could explain its previously noted sensitivity to habitat pollution. Intriguingly, this repertoire includes a cytochrome P450 gene not previously described in any insect genome. The *C. splendens* immune gene repertoire appears relatively complete and features several genes encoding novel multi-domain peptidoglycan recognition proteins. Analysis of chemosensory genes revealed the presence of both gustatory and ionotropic receptors, as well as the insect odorant receptor coreceptor gene (*OrCo*) and at least four partner odorant receptors (*ORs*). This represents the oldest known instance of a complete OrCo/OR system in insects, and provides the molecular underpinning for odonate olfaction. The *C. splendens* genome improves the sampling of insect lineages that diverged before the radiation of Holometabola and offers new opportunities for molecular-level evolutionary, ecological, and behavioral studies.

## Introduction

The banded demoiselle *Calopteryx splendens* [from the Greek καλóς (*kalos*), meaning “beautiful”, and πτɛ´ρυξ (*pteryx*), meaning “wing”] is a species of the damselfly family Calopterygidae. With about 150 described species, Calopterygidae represent a relatively species-poor lineage of damselflies (Allen et al. 1984). Calopterygidae are found on all continents except Australia and New Zealand. Damselflies (Zygoptera) constitute together with the dragonflies (Anisoptera) the insect order Odonata. Molecular analyses corroborate that Odonata and Ephemeroptera (mayflies) form the winged insect clade Palaeoptera, which diverged from other winged insects roughly 406 Ma (Misof et al. 2014). Odonates have been extensively used as models for ecological, evolutionary, and behavioral studies (Cordoba-Aguilar and Cordero-Rivera 2005). Damselfly species of the genus *Calopteryx* have a complex life cycle, with a drastic habitat shift from the immature to the adult life stage (Stoks and Cordoba-Aguilar 2012). *C* *alopteryx* *splendens* has a bi-annual life cycle with an aquatic naiad stage (Brooks and Lewington 1997). The adults devote their time to feeding and increasing their fat reserves, which contributes to their subsequent mating success. The courtship behavior of many Calopterygidae, including *C. splendens*, has been studied in great detail (Cordoba-Aguilar and Cordero-Rivera 2005) and includes an initial stage during which the male removes the sperm stored by the female in her storage organs (Waage 1979). After copulation, the male guards the female in order to prevent her from mating with other males before oviposition.

Despite *C. splendens* being such an important model species for studying ecology, evolution, and behavior, the molecular nature of its studied traits has not been readily accessible to date. Very few nucleotide sequences referring to calopterygids are currently deposited in GenBank: ∼460 nucleotide sequences refer to species of the genus *Calopteryx*, 74 of which are specifically from *C. splendens*. The majority of these sequences are mitochondrial. In fact, such an underrepresentation with regard to genomic resources holds for most nonholometabolan insect clades, except for only a few genomes from representatives of Psocodea (Kirkness et al. 2010), Hemiptera (International Aphid Genomics Consortium 2010; Mesquita et al. 2015; Benoit et al. 2017; Rosenfeld et al. 2017), Isoptera (Terrapon et al. 2014), Phasmatodea (Soria-Carrasco et al. 2014), and Orthoptera (Wang et al. 2014). Without genomic resources for species of Palaeoptera, attempts to elucidate the molecular basis of the early evolution of traits in winged insects remain limited.

In this study, we sequenced and analyzed the draft genome of the damselfly *C. splendens*. This genome sequencing project contributes to the *i*5K initiative, whose target is to sequence the genomes of 5,000 arthropods (Robinson et al. 2011; i5K Consortium 2013). Moreover, it is one of the few efforts aimed at sequencing insect species from clades that diverged early in the evolution of winged insects. Of more than 100 currently publicly available insect genomes, the vast majority are of Holometabola and in particular of flies, mosquitoes, ants, and bees (www.orthodb.org; last accessed January 30, 2017). This generally reflects their roles as disease vectors, model species, and/or pollinators. Importantly, in order to understand the evolution of insect genes and genomes, we need to expand species sampling to include the tremendously diverse nonholometabolan insect orders. However, many hexapod clades, such as Palaeoptera (damselflies, dragonflies, and mayflies), Polyneoptera (mantids, termites, roaches, grasshoppers, and relatives), Zygentoma (silverfish), Archaeognatha (bristletails), Diplura (forcepstails), Collembola (springtails), and Protura (coneheads) remain unexplored or are very underrepresented. According to the Animal Genome Size Database (Gregory et al. 2007), many of these tend to have genomes that are much larger than those of most other sequenced insects, and this presents a challenge that undoubtedly plays a role in delaying the genome sequencing of species from these lineages. Thus, the sequencing of the *C. splendens* draft genome is a tremendously important step in augmenting available genomic information for insect clades that diverged before the radiation of Holometabola.

At 1.6 Gbp, *C. splendens* has one of the largest arthropod genomes currently sequenced, larger in size than the genome of the *Aedes aegypti* mosquito (1.3 Gbp) (Nene et al. 2007) and smaller than that of the deer tick, *Ixodes scapularis* (2.1 Gbp) (Gulia-Nuss et al. 2017) and the migratory locust, *Locusta migratoria* (6.3 Gbp) (Wang et al. 2014). Comparison of the gene set of *C. splendens* with the gene sets of other arthropods revealed that it contains features not seen in any other currently available sequenced insect genome. Our study shows that certain characteristic features of winged insects, such as chemoreceptors, appeared early in the evolution of insects. Other features, such as the presence of a cytochrome P450 protein, and the domain structure of peptidoglycan recognition proteins (PGRPs), a family of immunity proteins, seem to be unique to Odonata.

## Materials and Methods

### DNA Extraction, Sequencing, Assembly, and Annotation

Individuals were field-collected at Freckenfeld, Germany (49.056928, 8.139490; June 07, 2013; legit Dr. M. Niehuis), and their genome size was estimated to ∼1.7 Gbp using flow-through cytometry and applying the protocol outlined by DeSalle et al. (2005) (the genome of *Acheta domestica*, at ∼3.9 Gbp, served as size standard). Genomic DNA of female individuals was extracted from legs and head using the Qiagen DNeasy Blood & Tissue kit (Qiagen, Hilden, Germany) and following the “insect” nucleic acid isolation protocol described by the manufacturer. We produced four short-insert libraries from the isolated genomic DNA using Illumina’s TruSeq DNA Nano kit (Illumina, Inc., San Diego, CA, USA), using 550 bp of target insert size, according to the manufacturer’s protocol. Long-insert libraries were produced using Illumina’s Nextera Mate Pair kit with size selection performed on precast E-gel (Life Technologies, Europe BV) 0.8% agarose gels. Four long-insert libraries were prepared in total, with 3 kbp (two libraries), 6 and 9 kbp insert sizes. We sequenced a total of 109 Gbp from the short-insert libraries and another 150 Gbp from the long-insert libraries on a HiSeq 2500 sequencer. All raw reads (∼2.6 billion) are deposited in the NCBI short-read archive (SRA) under accession numbers SRP075442 (BioProject: PRJNA315816).

Contig assembly was performed using SparseAssembler v. 2012-06-15 (Ye et al. 2012), and scaffolding was performed on contigs >200 bp in size using SSPACE v. 3.0 (Boetzer et al. 2011). Finally, short (<15 kbp) scaffolds were removed, as these usually contain repeats and fragmented genes. Results from the Benchmarking Universal Single-Copy Orthologs (BUSCO) pipeline v. 1.0 (Simao et al. 2015) were used when searching for optimal parameters during the individual assembly steps. Genes were structurally annotated with the aid of the MAKER pipeline v 2.31.8 (Campbell et al. 2014), resulting in a gene set comprising 22,523 genes. Extrinsic evidence provided to MAKER were 1) transcripts of adult *C. splendens* (both sexes) sequenced in the context of the 1KITE project (www.1kite.org; last accessed January 30, 2017), 2) arthropod proteomes obtained from OrthoDB v8 (Kriventseva et al. 2015), and 3) all entries from the SwissProt protein database (Bairoch et al. 2004). Functional annotation of predicted protein-coding genes was performed using 1) InterProScan v. 5.13-52 (Jones et al. 2014) for finding conserved domains and 2) BLASTP v. 2.2.28+ (Altschul et al. 1997; Camacho et al. 2009) against Uniref50 (Suzek et al. 2015) for finding conserved functions. The analysis details are described in supplementary text, Genome assembly and annotation (Supplementary Material online).

### Comparative Genomics

The predicted gene set of *C. splendens* was searched against the gene sets of all arthropods contained in the OrthoDB v8 database (Kriventseva et al. 2015), and all genes in the gene set of *C. splendens* were mapped against the already delineated orthologous groups. Subsequently, a phylogenomic analysis was conducted based on 642 single-copy orthologs present in *C. splendens* and each of another eight arthropods: *Drosophila melanogaster*, *Danaus plexippus*, *Tribolium castaneum*, *Apis mellifera*, *Acyrthosiphon pisum*, *Pediculus humanus*, *Zootermopsis nevadensis*, and *Daphnia pulex*. In addition, the orthologous amino acid sequences (identified from transcriptome data with the BUSCO pipeline) of the azure damselfly, *Coenagrion puella* (Johnston and Rolff 2013), and the blue-tailed damselfly, *Ischnura elegans* (Chauhan et al. 2014), were added to the phylogenomic analysis. To perform an initial unbiased analysis of gene families we took a conservative approach and used blastclust 2.2.9 from the BLAST+ package (Camacho et al. 2009) to infer gene families by clustering the *C. splendens* genes with those of the other seven insect species (supplementary text, Protein families, Supplementary Material online). For the families of particular interest we then undertook a more comprehensive analysis including detailed manual curation. All phylogenetic analyses were performed using MAFFT v. 7.050b (Katoh and Standley 2013) for multiple sequence alignment, Trimal v. 1.2rev59 (Capella-Gutierrez et al. 2009) for automatic trimming, and RaxML v. 7.6.6 (Stamatakis 2006) for inferring phylogenetic hypotheses under the maximum likelihood optimality criterion. Details can be found in supplementary text, Phylogenomics and orthology (Supplementary Material online). The resulting phylogenetic trees were viewed and annotated with EvolView (He et al. 2017) and Inkscape v. 0.91.

Genes encoding cytochrome P450s monooxygenases, glutathione *S*-transferases (GSTs), and choline/carboxylesterases were identified by searching for the corresponding InterPro domains and by means of BLAST searches using reference proteins from other insect species (supplementary table S1, Supplementary Material online). More details can be found in supplementary text, Detoxification enzymes (Supplementary Material Online). Genes and gene families that make up the canonical immune gene repertoire in other insects (Waterhouse et al. 2007; Bartholomay et al. 2010; Barribeau et al. 2015) and more distantly related arthropods (Palmer and Jiggins 2015) were identified by employing orthology assignments, sequence homology searches, and considering the occurrence of characteristic InterPro domains. More details can be found in supplementary text, Immunity (Supplementary Material online). Reference chemoreceptor genes from the termite *Z. nevadensis* (Terrapon et al. 2014) were used to exhaustively search for chemoreceptors in *C. splendens* (supplementary text, Chemoreceptors, Supplementary Material online). Similarly, reference opsins (supplementary table S2, Supplementary Material online) were used to search for the corresponding genes in the genome of *C. splendens* by using BLAST, HMMer, and also by identification of characteristic InterPro domains (supplementary text, Opsins, Supplementary Material online). All genes whose protein sequences were used to build gene family phylogenetic trees were carefully inspected, and any incomplete or inaccurate gene models were manually curated to improve the annotations.

## Results and Discussion

### One of the Largest Insect Genomes Sequenced so Far

We sequenced the genome of the banded demoiselle, *C. splendens*, using four short-insert and four long-insert libraries (see Materials and Methods for details). The starting material for library construction was the legs and heads of two individual females. We avoided using the abdomen in order to minimize contamination with bacterial and eukaryotic microorganisms from the gut. This approach generated 109 Gbp from short insert libraries and resulted in an estimated sequencing depth of 65.5 (table 1). The resulting draft genome assembly of *C. splendens* consists of 8,896 scaffolds with a total assembly size of 1.63 Gbp, of which 305 Mbp are gaps. The genome is estimated to be 1.7 Gbp (see Methods), which means that most of the genome is likely covered by this assembly. Contig N50 and scaffold N50 are at 3.1 and 422.3 kbp, respectively, with a maximum scaffold length of 2,779.5 kbp (table 1). The assembly contains 85.3% of the 2,675 single-copy genes conserved in arthropods as assessed by the BUSCO tool (Simao et al. 2015). However, assembly gaps and fragmentation resulted in recovery of only 53.5% of them as complete genes, leaving 31.8% as fragments (table 1). Nevertheless, assessing *C. splendens* using a subset of 801 highly conserved arthropod orthologs from OrthoDB v9 recovered 95.3% complete matches in the genome (supplementary table S3, Supplementary Material online). Additionally, the presence of the conserved *TipE* gene cluster (Li et al. 2011) was confirmed in the damselfly genome, with the expected ancestral arrangement (supplementary fig. S1, Supplementary Material online), indicating that the assembly has successfully recovered conserved insect gene clusters. Finally, a fraction (18.6%) of the assembly corresponds to repetitive sequences (supplementary table S4, Supplementary Material online), most of which seem to be species-specific.

**Table 1:** Features of the *Calopteryx splendens* Genome Assembly and Gene Annotation and Comparison to Those of Other Arthropods

	CSPLE^a^	ZNEVA^b^	APISU^c^	ISCAP^d^	AALBO^e^	AAEGY^f^	LMIGR^g^
Estimated genome size (Mbp)	1,670	562	517	2,100	606-1,623	1,317	6,300
Assembly size (Mbp)	1,630	494	464	1,800	1,967	1,384	6,500
Sequencing technology	Illumina	Illumina	Sanger	Sanger	Illumina	Sanger	Illumina
Sequencing coverage (x)	65.5	98.4	6.2	3.8	350	7.6	114
Total no of contigs	1,278,437	–^h^	72,844	570,640	607,139	36,206	1,438,086
Contig N50 (kbp)	3.1	20.0	10.8	2.9	17.3	82.6	9.3
Total no of scaffolds	8,896	93,931	23,924	369,495	401,027	4,758	551,270
Scaffold N50 (kbp)	422.3	740.0	88.5	51.5	195.5	1,500.0	320.3
Max scaffold length (kbp)	2,780	5,111	3,073	3,952	1,305	5,856	7,903
Number of genes predicted	22,523	15,876	34,604	20,486	17,539	15,419	17,307
Mean exon length (bp)	193	210	255	855	1,381	–^h^	1,160
Average no of exons/gene	5.0	6.1	4.5	–^h^	3.3	4.0	5.8
BUSCO^i^ (genome)	53.5 (1.7), 31.8, 14.7	75.7 (2.6), 20.8, 3.5	60.5 (6.1), 19.1, 20.4	57.4 (2.6), 28.2, 14.4	83.2 (24), 12.6, 4.2	80.4 (11), 15.7, 3.9	32 (2.8), 36, 30
BUSCO^i^ (gene set)	68.6 (5.9), 21.2, 10.2	94.9 (9.8), 3.9, 1.2	89.9 (14), 4.1, 6	69.4 (6.6), 23.4, 7.2	83 (23), 6.3, 10.7	93.3 (17), 3.6, 3.1	76 (10), 12, 10

a Abbreviations used for species names; CSPLE, *Calopteryx splendens*; ZNEVA, *Zootermopsis nevadensis*; APISU, *Acyrthosiphon pisum*; ISCAP, *Ixodes scapularis*; AALBO, *Aedes albopictus*; AAEGY, *Aedes aegypti*; LMIGR, *Locusta migratoria*.

b Numbers taken from Terrapon et al. (2014).

c Numbers taken from International Aphid Genomics Consortium (2010).

d Numbers taken from Gulia-Nuss et al. (2016).

e Numbers taken from Chen et al. (2015).

f Numbers taken from Nene et al. (2007).

g Numbers taken from Wang et al. (2014).

h Numbers not provided in the corresponding manuscript.

i BUSCO completeness scores are in the format: % complete BUSCOs (of which, duplicated), % fragmented BUSCOs, % missing BUSCOs.

Automatic protein-coding gene annotation resulted in 22,523 predicted gene models, 205 of which were manually inspected. The average number of exons per gene in this set of genes is 5.0, which is in the same range as those inferred from analyzing other relatively large arthropod genomes (table 1). There are 16,155 genes that had either a functional annotation with InterProScan or a significant (*e*-value <1e-05) BLASTP hit in the Uniref50 database. The most abundant InterPro entries, excluding the ones related to transposable elements, are WD40 repeat (IPR001680), ankyrin repeat-containing domain (IPR020683), and RNA recognition motif domain (IPR000504) (supplementary table S5, Supplementary Material online). The most abundant gene ontology terms found were those for binding functions (*n* = 5,528 genes), metabolic processes (*n* = 3,583), cellular processes (*n* = 3,154 genes), and catalytic activities (*n* = 3,045) (supplementary fig. S2, Supplementary Material online). Finally, a blastclust-based clustering was performed to highlight the most over- and under-represented gene families in *C. splendens* shown in supplementary figure S3, Supplementary Material online (details can be found in supplementary text, Protein families, Supplementary Material online).

In an attempt to remove possible contaminant sequences, the raw reads as well as the genomic scaffolds were searched for similarity to genomic sequences of gregarine parasites, known to infect species of Odonata (Cordoba-Aguilar and Cordero-Rivera 2005; Stoks and Cordoba-Aguilar 2012), and also for similarity to bacterial genome sequences (supplementary text, Identification of contamination, Supplementary Material online). While there was no significant similarity to gregarine sequences, there were ∼550,000 reads with similarity to nucleotide sequences of bacteria. The majority of these possible bacterial reads exhibited the highest similarity to *Wolbachia*, the most common arthropod endosymbiont (Duron et al. 2008). However, assembly of these reads did not result in assembling a full bacterial genome. We also found only 50 genes in the damselfly gene set that are likely of bacterial origin, with 20 of these genes having a significant similarity to *Wolbachia* genes. These genes (supplementary table S6, Supplementary Material online) could theoretically also be acquired from *Wolbachia* via one or more lateral gene transfer events, which is apparently very frequent in *Wolbachia*-insect symbiotic relationships (Robinson et al. 2013). It should be noted that *Wolbachia* infections in odonates are not frequent and have been found in only five species so far (two damselflies and three dragonflies) (Thipaksorn et al. 2003; Wiwatanaratanabutr and Zhang 2017). Neither of these studies, however, tested for the presence of *Wolbachia* in any calopterygid damselflies.

### Odonates Are a Sister Lineage to Neopteran Insect Orders

**Fig. 1.—:**
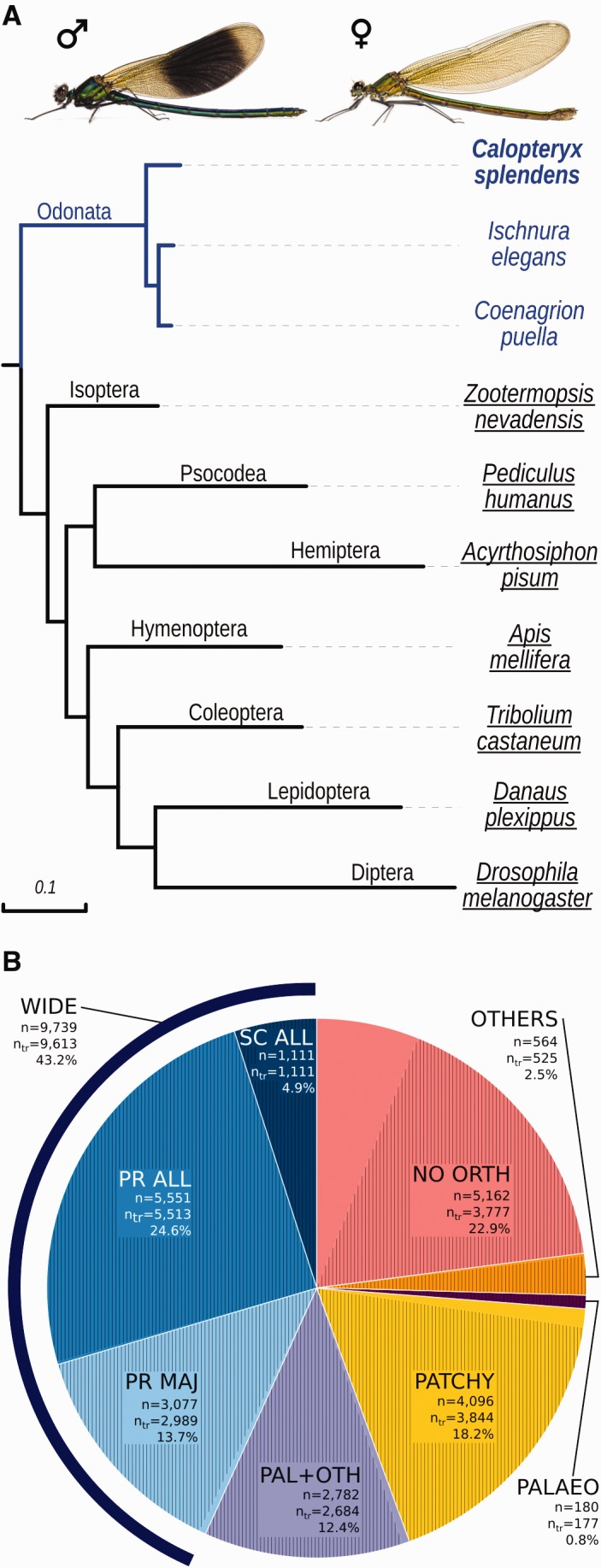
(*A*) Phylogenetic position of the damselfly *Calopteryx splendens*, relative to other insects, based on the phylogenetic signal of 642 single-copy protein-coding genes. The phylogenetic position of *Ischnura elegans* and *Coenagrion puella* was inferred from studying transcripts of the above genes in the corresponding transcriptomes. The tree is rooted using the crustacean *Daphnia pulex* and distances represent amino acid substitutions per site. All nodes received statistical bootstrap support >98%. *Calopteryx splendens*, *I. elegans* and *C. puella* are all damselflies and belong to the insect order of Odonata. Also, note the characteristic dark wing spots in *C. splendens* males. (*B*) Orthology profile for the *C. splendens* proteome (22,523 genes). Shaded areas represent the fraction of genes having a match in at least one of the available damselfly transcriptomes (*C. splendens*, *I. elegans*, *C. puella*). The number of genes (*n*=) and the number of transcribed genes (*n*_tr_=) for each part of the pie graph are also shown. *Calopteryx splendens* was compared with another ten arthropod species, which included all underlined species from Figure 1*A*, the outgroup *D. pulex* and the as yet unpublished genomes of the dragonfly *Ladona fulva* and the mayfly *Ephemera danica*. The two damselfly transcriptomes were used for assessing whether a *C. splendens* gene is transcribed, but not for determining orthology. Abbreviations used; SC ALL, single copy in all species; PR ALL, present in all species; PR MAJ, present in the majority of species; PAL + OTH, present in Palaeoptera (*C. splendens*, *L. fulva*, and *E. danica*) and at least one other species; PATCHY, present in *C. splendens* and at least one other species; PALAEO, present only in Palaeoptera; OTHERS, present in *C. splendens* and one or more Arthropod species, other than the selected ten species; NO ORTH, no orthology with any other arthropod species; WIDE, widely conserved.

We conducted a phylogenomic analysis of 642 single-copy orthologs across eleven different arthropod species. Ten of those were insect species and represented the insect orders Diptera, Lepidoptera, Coleoptera, Hymenoptera, Hemiptera, Psocodea, Isoptera, and Odonata. In addition, the genome of the water flea, *Daphnia pulex* was included as a noninsect outgroup. The resulting phylogeny places *C. splendens* (Odonata) together with two other damselfly species, *Ischnura elegans* and *Coenagrion puella*, whose transcriptomes have recently been published (Chauhan et al. 2014; Johnston and Rolff 2013), as a sister lineage to all other analyzed neopteran insect orders (fig. 1*A*). This result is consistent with the reconstruction presented by Misof et al. (2014).

In addition to the phylogenomic analysis, we conducted an orthology analysis based on the predicted gene set. Using the OrthoDB database (Kriventseva et al. 2015), the predicted 22,523 genes of the *C. splendens* draft genome were classified into different categories based on their occurrence in ten other arthropod species (fig. 1*B*). These species include those that are highlighted by underscores in figure 1*A*, the outgroup taxon *D. pulex*, and the genomes of the dragonfly *Ladona fulva* (Odonata) and the mayfly *Ephemera danica* (Ephemeroptera) (dragonfly and mayfly genome data are currently unpublished, but are used here for this analysis with permission, see acknowledgements). A considerable fraction of the damselfly genes (*n* = 9,739 or 43% of the total genes) have orthologs in all or most of the other species (categories SC ALL, PR ALL, and PR MAJ in fig. 1*B*), and the vast majority of these (*n* = 9,613) also has matching transcripts in the *C. splendens* transcriptome, or in the transcriptomes of the other two damselflies (*I. elegans* and *C. puella*). However, almost one-quarter of the genes (*n* = 5,162) shows no orthology with genes of other arthropods and for 1,385 of them, we also lack evidence for them being transcriptionally active. We further found a small fraction of genes (*n* = 180) that appear taxonomically restricted to Palaeoptera (i.e., Odonata and Ephemeroptera: *C. splendens*, *L. fulva*, and *E. danica*). Virtually all of them (*n* = 177) appear to be transcribed, but no InterPro domains were enriched in this category.

### A Relative Paucity of Detoxification Gene Family Members

*Calopteryx* *splendens* is, ecologically, an insect apex predator with a relatively long life span. Thus, detoxification of xenobiotic compounds is very important for this species, because xenobiotic compounds can accumulate in the body of the damselfly over time. Additionally, it is known that odonates are vulnerable to various pesticides such as chlorpyrifos (Arambourou and Stoks 2015), fipronil (Kasai et al. 2017), and spinosad (Jones and Ottea 2013). To investigate the set of enzymes involved in the protection against harmful compounds, we studied the three major groups of enzymes commonly associated with detoxification of xenobiotics: Cytochrome P450 monooxygenases (CYPs), carboxyl/cholinesterases (CCEs) and GSTs (Li et al. 2007). It should be noted, however, that certain classes of detoxification enzymes can have physiological functions other than detoxification (Gilbert and Auld 2005; Johnson and Moore 2013; Clayton et al. 1998; Singh et al. 2001; Li et al. 2008; Sawicki et al. 2003).

#### Cytochrome P450 Monooxygenases

We identified 56 putative *CYPs* in the draft genome assembly of *C. splendens* whose amino acid sequences were aligned with *CYP* genes of the fruit fly *D. melanogaster* and the copepod *Paracyclopina nana.* The copepod *CYPs* (Han et al. 2015) were used to assign the *CYPs* of *C. splendens* to *CYP* clans of arthropods (Feyereisen 2006) that are not found in the fruit fly. The *C. splendens CYP* genes belong to clan 2 (20 genes), clan 3 (18 genes), clan 4 (eight genes), and the mitochondrial clan (nine genes). Possessing genes in these four clans is typical for insects (Feyereisen 2006). However, compared with other insects, *C. splendens* has a high number of *CYPs* in clan 2, comparable to the number of clan 2 *CYPs* in *P. nana* and *D. pulex*. In contrast, the number of *CYPs* in clans 3 and 4 is smaller than in other insects (supplementary table S1, Supplementary Material online). While such low gene numbers could explain the susceptibility of *C. splendens* to certain xenobiotics, it should be noted that there is no clear correlation between the size of detoxification-related gene families and the resistance to xenobiotics (Rane et al. 2017). Finally, the number of mitochondrial *CYPs* is very similar among the investigated species (supplementary table S1, Supplementary Material online).

**Fig. 2.—:**
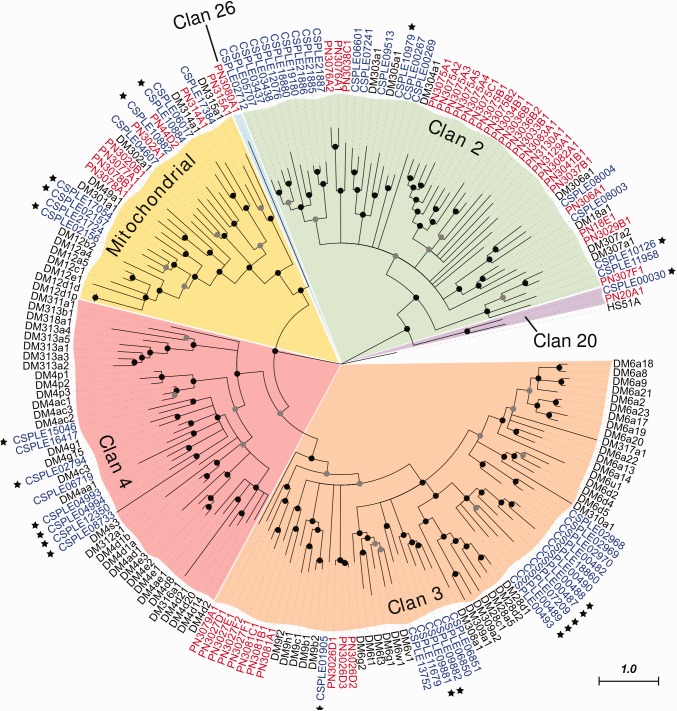
Maximum likelihood phylogenetic tree of CYP amino acid sequences from *Calopteryx splendens* (CSPLE, in blue), *Drosophila melanogaster* (DM, in black) and the marine copepod *Paracyclopina nana* (PN, in red). The tree was rooted with the human (HS) *CYP51* gene as an outgroup. Nodes with <50% bootstrap support collapsed into multifurcating nodes, nodes with bootstrap support between 50% and 75% are indicated with gray circles and nodes with bootstrap support >75% are indicated with black circles. Stars indicate transcript evidence for the *C. splendens* CYP450s. Different colored labels indicate the *CYP* clans, which include the four known insect clans (CYP2, CYP3, CYP4, and mitochondrial) as well as CYP26 and CYP20 clans. The CYP20 clan was for the first time identified in an insect species.

Interestingly, our phylogenetic analysis uncovered the presence of one *CYP* gene (CSPLE_00030) belonging to clan 20 (fig. 2). We were able to confirm the presence of CSPLE_00030 orthologs in the genomes of two other available Palaeoptera: *L. fulva* and *E. danica* (identified copies exhibited 81% and 54% amino acid sequence similarity, compared with the orthologous protein of *C. splendens*). To the best of our knowledge, this is the first time that a gene belonging to clan 20 has been found in an insect genome. Because it is found in all three palaeopteran genomes, it is possible that this *CYP* clan has been lost in the lineage that later gave rise to Neoptera. Outside of Hexapoda it is present in many organisms from anemones and sponges to humans (Nelson et al. 2013). The putative ortholog in the human genome is CYP20A1 (34% amino acid identity), which displays peculiar structural features, suggesting unusual catalytic functions (Stark et al. 2008). Even though in humans it is transcribed in many different tissues, its specific function is still unknown (Nebert et al. 2013).

#### Carboxyl/Cholinesterases

By manually curating the damselfly gene set we identified 22 CCE genes that we subsequently used for phylogenetic analysis. We used the functional categorization of *CCEs* proposed by Oakeshott et al. (2010) to assign *CCEs* to different CCE classes (supplementary fig. S4, Supplementary Material Online). The *C. splendens CCEs* represent seven of the 13 major insect CCE clades, with 14 genes in the neuro/developmental and cell adhesion class (clades H, L, M, K, and J), three genes in the hormone/semiochemical processing class (clades D and E), and no genes in the dietary/detoxification class (clade B). It should be noted that one of the genes belonging to the hormone/semiochemical processing class (CSPLE_05872) has a relatively low bootstrap support (<75%). Our detailed analysis revealed that the number of *CCE* genes identified in *C. splendens* is below average, with only the honey bee having a similar number of CCE genes (*n* = 24) (supplementary table S1, Supplementary Material online). While *C. splendens* may harbor additional CCE genes not present in our assembly, this initial analysis points to a paucity of CCEs that could impact processes such as detoxification or hormone/pheromone degradation.

#### Glutathione S-Transferases

A total of 18 putative *GSTs* were identified in the damselfly genome, 3 of which are microsomal GSTs whereas the remaining 15 are cytosolic. Phylogenetic analysis of the cytosolic GSTs from *C. splendens* and *D. melanogaster* (supplementary fig. S5, Supplementary Material Online) revealed that the damselfly GSTs belong to various different classes of cytosolic GSTs: Sigma, omega, theta, and zeta. The microsomal GSTs were not included in this analysis because they are considerably more diverged than cytosolic GSTs and thus hamper confident phylogenetic analysis. Three genes (CSPLE_11611, CSPLE_09480, and CSPLE_09481) do not cluster confidently with any GST class. Interestingly, *C. splendens* has eight members of the sigma class, whereas *D. melanogaster* has only one. Six of these sigma *GSTs* are collocated in the damselfly genome in a single cluster, on scaffold223 (supplementary fig. S6, Supplementary Material online). Similar to CCEs, the total number of *GST* genes in *C. splendens* is below average when compared with other insects (supplementary table S1, Supplementary Material Online).

### Robust Immunity with Novel Multidomain PGRPs

*Calopteryx* *splendens* is exposed to a variety of pathogens and parasites during its lifecycle, including fungi, bacteria, and viruses, and particularly suffers from ectoparasitic water mites and apicomplexan gregarine parasites. The ability to defend and recover quickly from infections is clearly vital for survival and reproductive success, and previous studies have indicated that the melanization response is particularly important in this context (Rantala et al. 2010; Rantala et al. 2011; Kaunisto et al. 2013). Especially in odonates, the melanization response has traditionally been used as an indication of immunocompetence either by indicating levels of phenoloxidase activity and/or encapsulation of pathogens by melanin (Moreno-Garcia et al. 2013). The *C. splendens* genome offers new opportunities to identify genes putatively involved in the immune defense responses of the damselfly.

#### The Immune Repertoire

Genome-wide searches for immune response-related genes in *C. splendens* led to the identification of the full complement of members of the major recognition, signal transduction, modulation, and effector insect immune-related gene families (supplementary table S7, Supplementary Material online). This finding supports earlier observations of a particularly complete immune gene repertoire in zygopterans based on transcriptome analyses of immune-challenged azure damselflies (*C. puella*) (Johnston and Rolff 2013). However, *C. splendens* immune-related gene families are generally not particularly larger or smaller than those of other insects, with the possible exception of caspases (cysteine aspartases involved in apoptosis) and *PGRPs* (binding and/or degrading peptidoglycan from bacterial cell walls). Orthologs of the three key insect immune pathway transcription factors are also present: The nuclear factor kappa-B (*NF-κB*) genes *Relish* (Imd pathway) and *dorsal* (Toll pathway), and the signal transducer and activator of transcription gene *STAT* (JAK/STAT pathway). Their upstream signaling factors were also identified, including orthologs of *caspar*, *Dredd*, *Fadd*, *Tak1*, *Tab2*, *Imd*, *cactus*, *Traf6*, *pelle*, *Myd88*, and *hopscotch*. We also identified transmembrane receptors, including several Toll-like receptors, long-type *PGRPs*, and the JAK/STAT pathway receptor gene *domeless*. This catalog of canonical immunity genes strongly suggests that all three major insect immune signaling pathways are likely to be fully functional in *C. splendens*. Furthermore, members of gene families known to modulate the cascade of signals that lead to melanization included many identified proteases, C-type lectins, serine protease inhibitors, as well as melanin-producing prophenoloxidases and a suite of peroxidases to deal with harmful reactive oxygen species during melanization. Future functional studies will be required to determine if any of these proteases and serine protease inhibitors modulate melanization responses and distinguish between those that control cascades leading to prophenoloxidase activation and those that trigger Toll pathway responses.

**Fig. 3.—:**
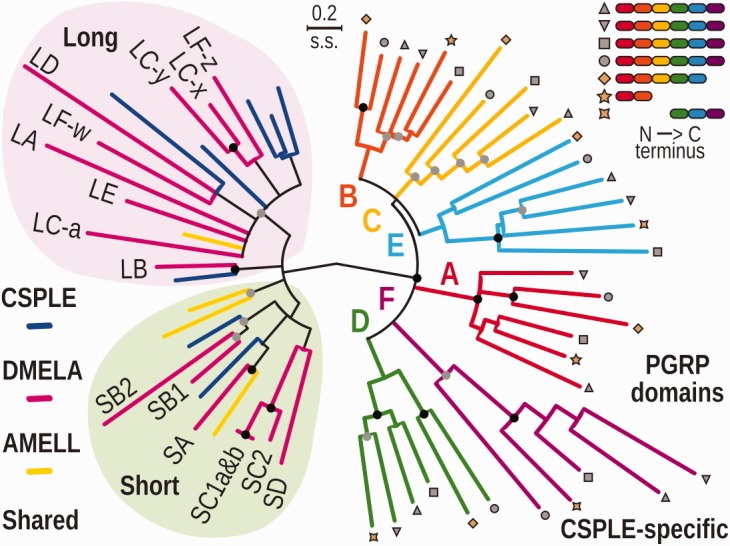
Molecular phylogeny of peptidoglycan recognition protein (PGRP) domains from *Calopteryx splendens* (CSPLE), *Drosophila melanogaster* (DMELA), and *Apis mellifera* (AMELL). The 28 shared domains from 25 genes (left) include both short and long-type PGRPs and are clearly distinct from the 34 domains from seven *C. splendens*-specific genes (right). Labeled leaves indicate *D. melanogaster* gene or domain names. Leaves marked with shapes (gray 6-domains, brown 5/3/2-domains) correspond to the domains from the damselfly-specific genes whose N-to-C terminus protein domain architectures are depicted (top right), color-matched to groups A to F of the phylogeny. The maximum likelihood phylogeny was estimated from the amino acid sequence alignment of PGRP domains with branch lengths representing substitutions per site. Nodes with <25% bootstrap support collapsed into multifurcating nodes, nodes with bootstrap support between 50% and 75% are indicated with gray circles and nodes with bootstrap support >75% are indicated with black circles.

In damselflies, melanin also forms in the wing-spots, where males with lighter, more heterogeneous melanin depositions showed higher parasite burdens than males with darker, homogenous distributions, suggesting that the degree of wing pigmentation is an honest indicator of a strong melanization activity in response to infection (Siva-Jothy 2000). Additionally, faster pathogen encapsulation through melanization was observed in males with larger wing-spots, and encapsulation rate was positively correlated with hemolymph hemocyte densities, suggesting overall better immunocompetence (Rantala et al. 2000). Thus, examining expression patterns of the identified prophenoloxidase genes could distinguish those that are important for wing-spot formation in males and those whose activity leads to parasite encapsulation immune responses in both sexes. Interestingly, it has been shown that the insect prophenoloxidase cascade is triggered by many different PGRP proteins (Yoshida et al. 1996; Royet et al. 2011), and in the fruit fly, PGRP-LE triggers the cascade (Takehana et al. 2004). Based on our phylogenetic analysis of the PGRP domains, there is no clear PGRP-LE ortholog in *C. splendens*, but there are several long-type PGRPs that could potentially perform this role (fig. 3; clade shaded in pink).

#### Peptidoglycan Recognition Proteins

Examining the expanded set of *PGRPs* revealed that, in addition to the canonical insect short and long-types, the *C. splendens* genome encodes several novel multi-domain *PGRP* genes (fig. 3). Four genes, three of which are neighbors on the same scaffold, each encode six divergent PGRP domains that are found in a single exon. The others are also single exons and encode five, three, and two domains, respectively. The PGRP domain amino acid sequence-based phylogeny clearly separates those domains shared with other insects and the *C. splendens*-specific domains from the multi-domain genes (fig. 3). Among the shared domains from short and long-type genes *C. splendens* has likely orthologs of *D. melanogaster PGRP-SA*, *PGRP-SB1/2*, and *PGRP-LB*, as well as an additional five long-type *PGRP* genes, each with a single domain. Genes with two domains are found in both insects and mammals, for example, *PGRP-LF* from *D. melanogaster* and mammalian *PGLYRP3* and *PGLYRP4* each have two domains (Royet et al. 2011). However, there are no examples reported to date of *PGRP* genes with six domains encoded in a single exon as found here for the first time in the *C. splendens* genome. Indeed, searching the complete UniProt archive (Uniprot Consortium 2015) for InterPro matches led us to identify only a few proteins with more than two domains and none with more than four domains. Examining these in detail (supplementary table S8, Supplementary Material online) led to the identification of an interesting case in the genome of *Drosophila willistoni* in which *PGRP-LF* appears to have acquired a third domain originating from a duplication of the LCa domain of the neighboring *PGRP-LC*, and possible three-domain blowfly and four-domain mussel proteins. However, the other proteins appear to be the result of erroneous gene annotations rather than representing true multi-domain *PGRP* genes. Finally, the genomes of the other two available Palaeoptera were also scanned, and even though no genes with multiple PGRP domains were found, there were cases of 1-domain *PGRP* genes located next to each other. The domains of the *C. splendens* six-domain PGRPs form six distinct groups (labeled A-F, fig. 3) that show a common N-to-C terminus protein domain architecture. The phylogeny shows that the last domain (F) is the most divergent and that the 5-domain gene corresponds to the first five domains, and the two- and three-domain genes to the first two and last three domains, respectively.

The *D. melanogaster PGRP-LC* gene has three domains each on a different exon that give rise to three different protein isoforms each with a single PGRP domain. Strikingly, in *C. splendens* all PGRP domains of the novel multi-domain genes are encoded by single exons. In mammals, these multi-domain PGRPs evolved by a domain duplication followed by a gene duplication (Montano et al. 2011), and mammalian PGRPs form disulfide-linked homodimers or heterodimers (Royet et al. 2011), effectively creating protein complexes of up to four domains. Hence, in *C. splendens* evolution may have followed an alternative route to give rise to multi-domain PGRPs. In insects, several extracellular PGRPs are known to trigger prophenoloxidase cascades, and sensing lysine-type peptidoglycan requires clustering of PGRP-SA (Park et al. 2007). It is consequently tempting to speculate that the novel *C. splendens* PGRPs may perform such roles, which may also explain the particularly robust melanization response of the damselfly. Future functional studies of these novel multi-domain genes will be needed to investigate these hypotheses and shed light on the evolution of insect immune responses in Odonata.

### Environmental Perception in a Predator

Prey detection and capture, and also maintaining hunting and courtship territory are essential to odonates and, as a result, perception of the environment is very important for them. In studying perception, we searched for genes involved in detection of chemical cues and light perception. Insects are well-known for their ability to detect chemical tastants and odorants using a variety of chemoreceptors. Yet, until recently (Rebora et al. 2012; Piersanti et al. 2014; Frati et al. 2015, 2017) odonate species were thought to be unable to detect odors, primarily based on visual and tactile stimuli for feeding and mating (Corbet 1980; Crespo 2011). Light perception, on the other hand, is accomplished by a family of G protein-coupled receptors (GPCR) called opsins, and damselflies and dragonflies are known to have an expanded repertoire of opsin genes (Futahashi et al. 2015). It thus proved worthwhile to investigate the repertoire of genes involved in chemosensation and light perception in the genome of the banded demoiselle and to compare it with that of other insect species with sequenced genomes.

#### Chemosensation

Three large families of chemoreceptors mediate most of the specificity and sensitivity of olfaction and taste in insects. Two of them, the gustatory receptor (GR) and odorant receptor (OR) families, are seven-transmembrane, ligand-gated ion channels (Benton 2015; Joseph and Carlson 2015), which are distantly related to each other in the insect chemoreceptor superfamily now known to be present even in basal animals (Robertson et al. 2003; Robertson 2015; Saina et al. 2015). The third family is the unrelated three-transmembrane ionotropic receptors (IR), which are variants of the ionotropic glutamate receptors that are also widespread in animals (Rytz et al. 2013). The genome of *C. splendens* was searched against a set of known chemoreceptors mainly from the termite *Z. nevadensis* and contains 51 *GRs*, five *ORs* and 20 *IRs* (supplementary text, Chemoreceptors, Supplementary Material online).

**Fig. 4.—:**
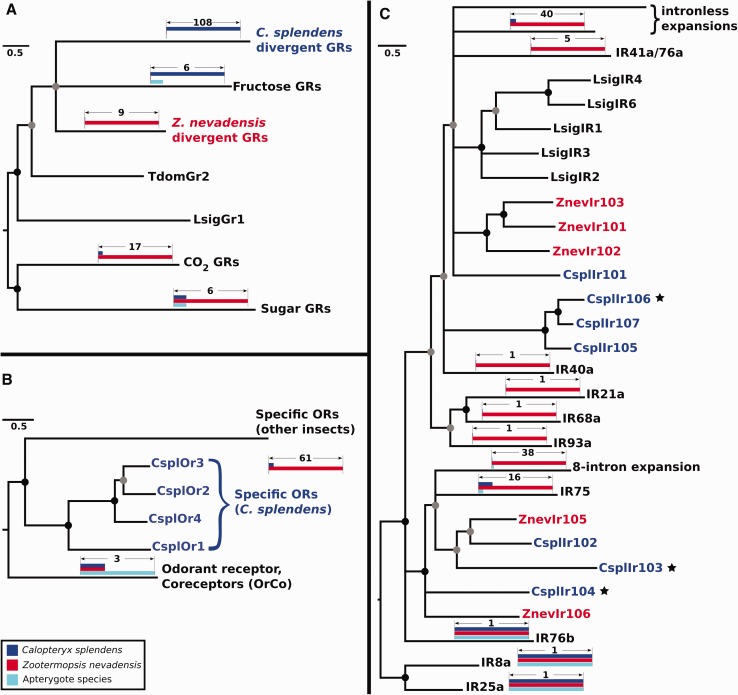
Phylogenetic analysis of the chemoreceptors identified in the genome of *Calopteryx splendens*. (*A*) The majority of the 115 gustatory receptors (GRs) belong to a species-specific expansion whose specificity is unknown. There are also genes with similarity to GRs for recognizing fructose, CO_2_, and sugars; (*B*) *C. splendens* contains only five odorant receptors (ORs). One of them is the conserved OrCo protein, whereas the remaining four are specific ORs, which appear as a sister group to specific ORs from other insect species. (*C*) Many conserved ionotropic receptors (IRs) were identified in the damselfly genome, in addition to an equal number of divergent IRs. In all three panels, damselfly genes are colored in blue and genes from the termite *Zootermopsis nevadensis* are colored in red. Transcribed genes are indicated with a star next to them. Nodes with <50% bootstrap support collapsed into multifurcating nodes, nodes with bootstrap support between 50% and 75% are indicated with gray circles, and nodes with bootstrap support >75% are indicated with black circles. Branch length scale is in substitutions per site. Abbreviations used for species names: Cspl, *C. splendens*; Znev, *Zootermopsis nevadensis*; Tdom, *Thermobia domestica*; Lsig, *Lepismachilis y-signata*.

The 51 *GR* genes code for 115 proteins, the vast majority of which belongs to a species-specific expansion. While the ligand specificity of this clade is not clear, they have similarities with bitter taste receptors in other insects. The seven remaining GRs belong to receptor subfamilies related to sugar (*n* = 1), carbon dioxide (*n* = 1), and fructose perception (*n* = 5) (fig. 4*A* and supplementary fig. S7, Supplementary Material online). More specifically, CsplGr1 clusters together with sugar GRs. These receptors function as dimers in *Drosophila* (Fujii et al. 2015), and all other insect genomes encode at least two sugar GRs. Therefore it is unclear how CsplGr1 might function as sugar receptor in *C. splendens*. Additionally, this subfamily appears to be very old in the insect lineage because it was found in the transcriptome of the bristletail *Lepismachilis y-signata* (Missbach et al. 2014), while it might even predate insect evolution because members of this subfamily have been found in Crustacea (Penalva-Arana et al. 2009). CsplGr2 belongs to a clade of putative carbon dioxide receptors. Despite the fact that their specificity is not clear in the bedbug and the termite, where this family expanded considerably (Benoit et al. 2017; Terrapon et al. 2014), it is clear that it is the lineage from which the holometabolan carbon dioxide GRs evolved. Finally, CsplGr3–CsplGr6 have a similarity to DmelGr43a, which functions as a fructose receptor in *D. melanogaster* (Miyamoto and Amrein 2014). Nevertheless, in the phylogenetic analysis they do not cluster confidently with DmelGr43a and the other fructose receptors (supplementary fig. S7, Supplementary Material online).

*Calopteryx* *splendens* contains five *OR* genes one of which is the conserved odorant receptor–coreceptor (*OrCo*), whereas the remaining four are specific ORs (fig. 4*B* and supplementary fig. S8, Supplementary Material online). *OrCo* is located in a genomic region where it is flanked by *CYP* genes (supplementary fig. S9, Supplementary Material Online). Such a low number of odorant receptors is consistent with the reduced olfactory abilities of odonates, but it remains unclear why they do not appear to have glomerular antennal lobes and mushroom body calyces usually involved in transmission of olfactory signals, structures that are present in apterygote firebrats for example (Farris 2005).

Robertson et al. (2003) speculated on the basis of a tree of the insect chemoreceptor superfamily of ORs and GRs from *D. melanogaster*, that the OR family might have evolved from a lineage of GRs early in the evolution of Insecta, perhaps in conjunction with the evolution of terrestriality. Missbach et al. (2014), however, could not identify OrCo or specific ORs in transcriptomic data of the wingless archaeognathan bristletail *L. y-signata.* Nevertheless, they discovered three OrCo-like proteins but no specific ORs in another wingless insect, the firebrat *Thermobia domestica* (Zygentoma), which most insect phylogenies indicate is a slightly more recent branch in the insect tree. They concluded that OrCo, at least, had evolved within insects, with specific ORs evolving after these wingless orders, perhaps by the Palaeoptera. Our finding of both a single OrCo and at least four specific ORs in this odonate indicates that the complete OrCo/OR system had indeed evolved by the time of the Palaeoptera.

To examine the relationships of these ORs further, our phylogenetic analysis included the three *T. domestica* OrCo proteins, a representative set of OrCo proteins from other insects, three ORs from the phasmatodid *Phyllium siccifolium*, also identified by Missbach et al. (2014), and a representative subset of the 69 ORs in *Z. nevadensis* (Terrapon et al. 2014). The resultant tree shows the confident clustering of the *C. splendens* OrCo with other insect OrCo proteins, while the four specific ORs form a distinct sister lineage to the termite and phasmatodid ORs (fig. 4*B* and supplementary fig. S8, Supplementary Material online), consistent with them representing early, specific ORs. It remains possible, however, that one or two of the *T. domestica* OrCo-like proteins, for example TdomOr1 and TdomOr3, in fact have evolved the role of a specific OR (Missbach et al. 2014).

We identified and named 20 *IR* genes (fig. 4*C* and supplementary fig. S10, Supplementary Material online). Among the predicted *IRs*, we identified orthologs to the conserved coreceptors *IR25a*, *IR8a*, and *IR76b*. Additionally, we identified orthologs of the conserved genes *IR40a*, *IR75a-c*, *IR21a*, *IR68a*, and *IR93a*. We also identified three genes that belong to the *IR75* clade, which is commonly expanded in other insects. Finally, there are ten more receptors belonging to highly divergent clades that are named IR101–IR110.

Ionotropic receptors have been implicated in both olfaction and gustation in *D. melanogaster* (Rytz et al. 2013), and some are even involved in detection of other stimuli such as temperature and humidity (Knecht et al. 2017). It is remarkable that in addition to the three conserved coreceptors, the IR93a, IR21a, IR40a, IR68a, and the IR75 clades are present in this palaeopteran, indicating that they are at least this old in the insect lineage, with the IR75 clade being even older. It remains unclear what role the divergent IR101–IR110 play in odonate chemosensation as they have only distant relationships with either the “antennal” or “divergent” IRs recognized in *Drosophila*, which generally are involved in olfaction and gustation, respectively (Rytz et al. 2013).

We also searched the damselfly genome for other proteins involved in chemosensation, such as odorant-binding proteins (OBPs) and chemosensory proteins (CSPs). OBPs are small proteins expressed by support cells at the base of chemosensory sensilla and secreted into the sensillar lymph where they are believed to bind and transport odorants from the atmosphere to chemoreceptors in the membranes of the dendrites of chemosensory neurons (Pelosi et al. 2006). Because OBPs are small and also fast-evolving their identification, using similarity-based methods, is particularly challenging. Consequently, it was not surprising that we found only four OBPs, three of which were fragmented (supplementary text, Odorant Binding Proteins, Supplementary Material online). CSPs are also soluble sensillar proteins but do not share a significant sequence similarity with OBPs. We identified seven putative CSPs, two of which grouped within the ancient 5-helical CSPs (Kulmuni and Havukainen 2013), although one of them (CSPLE_06529) with an intermediate bootstrap support (<75%) (supplementary fig. S11A, Supplementary Material online). Moreover, all *C. splendens* CSPs displayed four cysteines in the typical C1-X6-C2-X18-C3-X2-C4 pattern (supplementary fig. S11B, Supplementary Material online).

#### Opsins

**Fig. 5.—:**
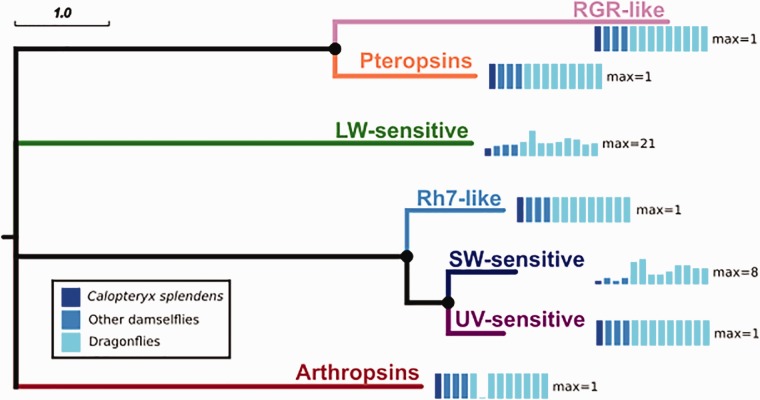
Comparison of the number of opsin genes found in each different group of opsins, in *Calopteryx splendens* and other odonates from Futahashi et al. (2015). The gene counts for each odonate species are shown as a small bar graph, where the maximum is noted at the right side of each graph. The counts for *C. splendens* are shown in dark blue, for the other damselflies (*Indolestes peregrinus*, *Mnais costalis*, and *Ischnura asiatica*) in light blue, and for the dragonflies (*Epiophlebia superstes*, *Anax parthenope*, *Asiagomphus melaenops*, *Tanypteryx pryeri*, *Anotogaster sieboldii*, *Macromia amphigena*, *Somatochlora uchidai*, *Orthetrum albistylum*, and *Sympetrum frequens*) in cyan. *Calopteryx splendens* has an opsin gene for each of the major groups but appears to have fewer LW-sensitive and SW-sensitive opsins than other odonates. The scale bar is in substitutions per site. Nodes with <50% bootstrap support collapsed into multifurcating nodes, and nodes with bootstrap support >75% are indicated with black circles. This tree is a pruned version of the full tree which is shown in supplementary figure S12, Supplementary Material online.

A set of 13 opsins in *C. splendens* was identified using BLASTP searches to known opsins from other animals (Hering and Mayer 2014). We conducted a phylogenetic analysis, in which we compared the damselfly opsins with opsins from a diverse range of odonates, consisting of another three damselflies and ten dragonflies (Futahashi et al. 2015). This analysis showed that the *C. splendens* opsins cluster together with all major opsin groups found in insects. More specifically, *C. splendens* has seven long wavelength (LW)-sensitive opsins, four of which are located next to each other on the same genomic scaffold. It should be noted, however, that two of the LW-sensitive opsins (CSPLE_20854 and CSPLE_13355) have a relatively low bootstrap support (<75%). Another six opsins were found in each of the following six opsin groups: Short wavelength (SW)-sensitive, UV-sensitive, Rhodopsin7-like, Pteropsin, RGR-like, and Arthropsin (fig. 5; the full tree is shown in supplementary fig. S12, Supplementary Material online). For the RGR-like and arthropsin groups no genes were predicted by the automatic genome annotation. However, upon searching the genome sequence we were able to find genomic fragments with significant similarity to these opsins from other odonate species (Futahashi et al. 2015). All 13 opsins have significant BLAST matches (*e*-value <1e-05) to transcripts from *C. splendens* or one of the other two publicly available damselfly transcriptomes (*C. puella* and *I. elegans*). Moreover, eleven of these genes contain the K296 retinal-binding residue (Palczewski et al. 2000), which further strengthens the possibility that they are typical opsins. The two genes that do not have the K296 residue are CSPLE_13355 (LW-sensitive) and the RGR-like opsin. CSPLE_13355 appears to be partial, because it is missing a coding sequence of about 120 amino acids in its N-terminus and another 130 amino acids from at least one exon. Upon examining the genomic area where this gene is located, it is apparent that the missing exons are most probably due to the presence of numerous gap regions, ranging in size from 50 to 500 bp. As for the RGR-like opsin, it seems that the conserved lysine residue is replaced by glutamic acid. The gene count for the SW-sensitive and LW-sensitive opsins is slightly lower than that inferred from studying other Odonata (Futahashi et al. 2015). Of course, such small differences could be explained by misannotations and need corroboration from additional experiments. The considerable expansion of the opsin gene family, as it is clearly shown for other odonates (Futahashi et al. 2015), is thought to be the result of an adaptation to the very different lifestyles of naiads and adults, the former being aquatic and the latter terrestrial. Another hypothesis, not necessarily incompatible with the previous one, is that the enhanced opsin repertoire is essential for hunting, because odonates are agile predators, being able to capture prey while flying (Mischiati et al. 2015). Interestingly, β-arrestins, the proteins regulating the activity of opsins and other GPCRs (DeWire et al. 2007) have been greatly expanded in *C. splendens*, compared with other insects (supplementary fig. S13 and supplementary text, Arrestins, Supplementary Material online).

## Conclusion

The draft genome of *C. splendens* is the first publicly available genome of a palaeopteran. The genome of such a nonholometabolan insect is valuable for comparative studies examining ancestral insect traits. Our analysis highlighted certain interesting aspects of the biology of this insect, such as the discovery of a CYP enzyme that has not been previously found in insects and is worth studying further. Moreover, the immunity-related proteins belonging to the PGRP family appear to have a peculiar structure, containing up to six PGRP domains, never observed before in an animal genome. Equally interesting is the finding of only a few ORs and a large complement of species-specific GRs, in the *C. splendens* genome. These findings suggest that the underlying molecular mechanism of common insect traits, such as detoxification of xenobiotics, immunity and olfaction, can be very different in clades other than the well-studied Holometabola. Thus, the banded demoiselle harbors genomic features that are as interesting as its truly splendid wings.

## Supplementary Material

Supplementary data are available at *Genome Biology and Evolution* online.

## Supplementary Material

Supplementary Data
