# Supplementary material for: Genomic Features of the Damselfly Calopteryx splendens Representing a Sister Clade to Most Insect Orders
Source: Genome Biol Evol. 2017 Jan 30;9(2):415–30. doi: 10.1093/gbe/evx006 (PMC5381652; doi:10.1093/gbe/evx006)
Supplement: Supplementary Data [file evx006_Supp.pdf]

Supplementary Material

Genomic features of the damselfly *Calopteryx splendens* representing a sister clade to most insect orders

Panagiotis Ioannidis, Felipe A. Simao, Robert M. Waterhouse, Mosè Manni,
Mathieu Seppey, Hugh M. Robertson, Bernhard Misof, Oliver Niehuis
and Evgeny M. Zdobnov

Table of Contents

A) Supplementary Text **3**
Genome assembly and annotation 3
Identification of contamination 4
Scanning reads 4
Scanning predicted genes 4
Phylogenomics and orthology 4
Protein families 5
Detoxification enzymes 5
Immunity 6
Chemoreceptors 7
The Gustatory Receptor (GR) family 8
The Odorant Receptor (OR) family 10
The Ionotropic Receptor family 12
Conclusion 13
Odorant binding proteins (OBPs) 14
Opsins 15
Arrestins 16
B) Supplementary figures and tables **17**
Figure S1. *TipE* gene cluster 17
Figure S2: GO terms 18
Figure S3: InterPro entries of interest 19
Figure S4: Maximum likelihood phylogenetic tree of CCEs 20
Figure S5: Maximum likelihood phylogenetic tree of GSTs. 21
Figure S6: Genomic region encoding a cluster of six sigma GSTs 22
Figure S7: Gustatory receptors in *Calopteryx splendens* and other insects 23
Figure S8: Odorant receptors in Calopteryx splendens and other insects. 24
Figure S9: Genomic region encoding the putative Orco gene. 25
Figure S10: Ionotropic receptors in *Calopteryx splendens* and other arthropods 26
Figure S11: Phylogenetic analysis of CSPs. 27
Figure S12: Phylogenetic analysis opsins 28
Figure S13: Phylogenetic analysis of arrestins 29
Table S1: Comparison of detoxification enzymes in different insect species. 30
Table S2: Accession numbers of the reference opsins 31
Table S3: BUSCO scores based on a highly conserved arthropod BUSCO set. 32
Table S4: Repetitive elements present in the genome of *Calopteryx splendens*. 33
Table S5: Top ten InterPro domains present in the gene set of *Calopteryx splendens* 34
Table S6: Calopteryx splendens genes with similarity to those of Bacteria. 35
Table S7: Counts of immune-related genes 36
Table S8: Results of searching UniParc for multi-domain PGRPs. 37
C) References **38**
D) Curated protein sequences **43**
Gustatory Receptors (GRs) 43
Odorant Receptors (ORs) 58
Ionotropic Receptors (IRs) 59
Odorant binding proteins (OBPs) 63
Calopteryx splendens 63
Ischnura elegans 63
Coenagrion puella 63

A) Supplementary Text

Genome assembly and annotation

Contig assembly was performed using SparseAssembler (Ye et al. 2012), with parameters "K 51 g 20 GS 2000000", where K is the k-mer size, g is the number of skipped k-mers and GS is the estimated genome size in Kbp. Scaffolding was then performed on contigs >200 bp, using SSPACE (Boetzer et al. 2011) with default parameters. A combination of different parameters was tested at both the contig assembly and scaffolding steps, in order to get the best assembly. The results of this "parameter scan" were evaluated using the BUSCO (Benchmarking Universal Single-Copy Orthologs) pipeline with the arthropod data set (Simao et al. 2015), in order to find the best possible assembly. The last step in genome assembly was to remove short scaffolds. Using BUSCO, we filtered out scaffolds <15 Kbp; removing larger scaffolds resulted in losing conserved arthropod genes. This resulted in an assembly consisted of 8,896 scaffolds, which is a relatively easy to work with, while the assembly size (1.63 Gbp) is also very close to the estimated genome size (1.7 Gbp). However, less conserved and/or short genes can possibly be found in the remaining 430,431 short (<15 Kbp) scaffolds. Therefore, we also made them available in our website for the scientific community. Preliminary analysis of these contigs showed that their vast majority does not contain a significant hit against the SwissProt database. More specifically, 21,871 (5.1%) short scaffolds have a significant (e-value <1e-05) match against a SwissProt entry, excluding transposable elements. Furthermore, of those 21,871 scaffolds, only 1,369 (0.3%) cover a >60% of the corresponding SwissProt entry.

It should be noted that reads from long insert libraries were only used for scaffolding, but not for contig assembly. The reason for this is because long insert libraries do not sample equally well the entire genome and this unequal sequencing coverage could lead to assembly errors and/or fragmentation. In fact, the few attempts where both short and long insert libraries were used for contig assembly, resulted in assemblies with a much lower contig N50. Moreover, these runs required more computational resources and also more runtime, such that it was not practical to perform the above-mentioned parameter scan.

Genes were annotated with the MAKER pipeline v. 2.31.8 (Campbell et al. 2014) and resulted in a gene set containing 22,523 genes. The evidence used were (a) *Calopteryx splendens* transcripts obtained from 1KITE, (b) arthropod proteomes from OrthoDB v8 (Kriventseva et al. 2015), and (c) the SwissProt protein database (Bairoch et al. 2004). Functional annotation was performed using InterProScan (Jones et al. 2014) for finding conserved domains and BLASTP (Camacho et al. 2009) against Uniref50 (Suzek et al. 2015) for finding conserved functions.

For assembling the above mentioned *C. splendens* transcriptome, one RNASeq library was prepared from whole body RNA extracts of pooled males and females. Sequencing resulted in 13,095,991 read pairs, or 3.9 Gbp of raw sequence data. Transcriptome assembly was done using SOAPdenovoTrans and generated 101,092 sequences (transcripts) comprising 39 Mbp.

Identification of contamination

We used two strategies to assess the occurrence of bacterial contamination in our libraries: by scanning (a) the reads, and (b) the predicted genes of the assembly for significant similarity to bacterial sequences. Such similarity would mean that the given damselfly sequence (read or gene) likely represents a bacterial contaminant. Alternatively, it could also mean that these sequences were acquired by lateral gene transfer (LGT), especially since there is an increasing number of studies that has documented such LGT events in various arthropods and other animals (Robinson et al. 2013).

Scanning reads

Reads from all eight libraries (four short-insert and four long-insert) were searched against the NCBI NT database using BLASTN. We found 549,172 reads (of a total ~2.6 billion reads) having a bacterial best match. Several attempts were made to assemble these reads but without success; we could only get very short contigs that very rarely contained whole genes. This finding suggested that there is very low contamination with bacterial reads. As a result, we decided to not remove any bacterial-like reads from our data set, especially since some of them could be the result of LGT.

Scanning predicted genes

The amino acid sequence of all 22,523 predicted damselfly genes was searched against the NCBI NR database, using BLASTP. There were only 50 genes in this *C. splendens* predicted gene set whose first BLASTP hit referred to bacteria. These sequences were further examined to determine if it is more likely to represent bacterial contaminants or potentially laterally transferred bacterial genes. To this end, a number of features was extracted for each of these genes, which are shown in table S6. As can be seen in this table, a considerable number of genes shows high similarity to *Wolbachia* bacteria. *Wolbachia* bacteria are common insect endosymbionts that have frequently transferred parts of their genome, or even its entire genome, to the nuclear genome of their arthropod host (Robinson et al. 2013). These genes are good LGT candidates and it would be interesting to verify their ancestry in future studies.

It should be noted what we also scanned our reads for possible contamination by sequences from gregarine parasites, which are known to infect Odonata (Cordoba-Aguilar and Cordero-Rivera 2005; Stoks and Cordoba-Aguilar 2012). First, we searched the reads using the NCBI NT database and found that there were only 1,069 reads matching some gregarine parasite. Second, we also searched the assembled scaffolds using the same nucleotide database (NCBI NT). No scaffolds, however, were had a best hit to a sequence originating from a gregarine parasite. The results from these two searches strongly suggest that gregarine contamination is negligible, both in the reads and also in the assembled scaffolds.

Phylogenomics and orthology

The predicted gene set was mapped against all arthropods in OrthoDB v8 (Kriventseva et al. 2015) and assigned to ortholog groups (OGs). Subsequently, a phylogenomic analysis was undertaken using OGs that had exactly one ortholog (i.e. single-copy orthologs) in each of the

following species: *Daphnia pulex* (water flea), *Zootermopsis nevadensis* (termite), *Pediculus humanus* (body louse), *Acyrthosiphon pisum* (pea aphid), *Apis mellifera* (honey bee), *Tribolium castaneum* (red flour beetle), *Danaus plexippus* (monarch butterfly) and *Drosophila melanogaster* (fruit fly). In addition, the BUSCO pipeline was used in order to extract single-copy orthologs from the transcriptome assembly of the azure damselfly, *Coenagrion puella* (Johnston and Rolff 2013) and the blue-tailed damselfly, *Ischnura elegans* (Chauhan et al. 2014). For studying the latter, we assembled its transcriptome from the deposited raw reads in SRA, since there was no publicly available assembly. The assembly was done using Trinity (Haas et al. 2013) with the default parameters.

Protein families

The protein sequence of *C. splendens* predicted genes was clustered with those of another seven insect species, using blastclust 2.2.9 from the BLAST+ package (Camacho et al. 2009), with a length coverage threshold >60% on both genes, and a percentage of identities threshold >35%. The seven insects used for this clustering scheme were: *Z. nevadensis*, *P. humanus*, *A. pisum*, *A. mellifera*, *T. castaneum*, *D. plexippus* and *D. melanogaster*. Families of interest with regard to this study, such as detoxification and immunity-related genes, chemoreceptors, and opsins were subsequently studied in depth. Other families, such as the β-arrestin family (see the section on arrestins below), that were seemingly over- or under-represented in *C. splendens* were also further studied. Finally, for identifying over-represented InterPro entries, these were extracted from the InterProScan results and filtered to keep entries covering >75% of the corresponding Pfam hidden Markov model (HMM) (v3.1b1).

Detoxification enzymes

Sequences encoding CYPs, GSTs, and CCEs were identified by searching for the corresponding InterPro domains (CYPs: IPR001128; GSTs: IPR003081, IPR005442, IPR003080, IPR003082; CCEs: IPR019819, IPR002018, IPR019826) in the InterProScan result file of the predicted protein set, as well as by BLAST (Camacho et al. 2009) searches using already known proteins for each superfamily from other insects species. A first manual analysis of the retrieved predicted peptides was performed with BLASTP searches against NCBI NR and Uniprot/SwissProt protein database. The predicted genes were then visualized and manually edited, when necessary, in the genome browser WebApollo (Lee et al. 2013). In some cases, when short sequences with partial domains were found, we attempted to retrieve more complete sequences using GeneWise (Birney et al. 2004). Manually curated sequences with at least 300 amino acids for CYPs/CCEs and 170 amino acids for GSTs were used for subsequent phylogenetic analyses. These length cutoffs were chosen based on the average length of proteins in the protein family. Predicted proteins were aligned with MAFFT (Katoh and Standley 2013), with the default parameters, to the corresponding proteins from *D. melanogaster*. For the CYP phylogeny, additional CYPs from *Paracylopina nana* (Copepoda, Cyclopoida) (Han et al. 2015) were added to the analysis in order to better resolve nodes. A maximum-likelihood phylogeny was created for each superfamily using RaxML (Stamatakis 2006), using the PROTGAMMAAUTO model and performing 100 bootstrap replicates. The trees were visualized and drawn with Evolview (He et al. 2016) and Inkscape v0.91. SignalP (Petersen et al. 2011) and TMHMM (Krogh et al. 2001) were used to infer subcellular localization and presence of transmembrane helices.

Immunity

Putative immune-related genes were identified in *C. splendens* and each of seven other insects and the crustacean *D. pulex* (table S7) by searches for characteristic InterPro domains of genes and gene families that make up the canonical immune gene repertoire in previously investigated insects (Waterhouse et al. 2007; Bartholomay et al. 2010; Barribeau et al. 2015) and other arthropods (Palmer and Jiggins 2015). Gene families involved in immune recognition phases included: Gram-negative bacteria-binding proteins (GNBPs), peptidoglycan recognition proteins (PGRPs), fibrinogen-related proteins (FREPs), galectins (GALEs), MD2-like proteins (MLs), scavenger receptor types A, B, and C (SCRAs, SCRBs, SCRCs), and thioester-containing proteins (TEPs). Immune modulators included: C-type lectins (CTLs), serine protease inhibitors (serpins, SRPNs), inhibitors of apoptosis (IAPs), and cysteine aspartases (CASPs). Immune effector families included: antimicrobial defensins (DEFs), lysozymes (LYSs), catalases (CATs), prophenoloxidases (PPOs), superoxide dismutases (SODs), haem peroxidases (HPXs), glutathione peroxidases (GPXs), and thiol peroxidases (TPXs). Genes involved in immune signaling pathways included: nuclear factor kappa-B (NF-κB) genes (RELs), Toll-like receptors (TOLLs), and spaetzles (SPZs), as well as Imd (immune deficiency) pathway members *caspar*, *Dredd*, *Fadd*, *Tak1*, *Tab2*, and *Imd*; Toll pathway members *cactus*, *Traf6*, *pelle*, *Myd88*, and *tube*; and JAK/STAT (janus kinase - signal transducer and activator of transcription) pathway members *STAT*, *hopscotch*, and *domeless*.

C. splendens orthologs of all immune signaling pathway members were identified, except for the death domain-containing gene *tube*, which in *D. melanogaster* interacts with *pelle* and *Myd88* (Towb et al. 2009). Representatives of all gene families were also identified, and these genes were checked for the proportions of the InterPro domain profiles that they matched to reach a more conservative count of genes with domains that match more than 75% of the corresponding profile. Pfam profiles were used unless the domain had no corresponding Pfam profile, in which case Superfamily (LYSs & HPXs), SMART (PGRPs), and PANTHER (SODs) were used. In general, *C. splendens* immune-related gene families are not especially larger or smaller than those of other insects, although families of CASPs and PGRPs appear to have expanded. Only one GNBP and one DEF gene were identified, but their presence suggests that *C. splendens* is capable of GNBP and DEF-mediated immune responses even if some other arthropods have more of these types of genes, particularly of GNBPs. The immune gene catalogue is therefore generally complete, suggesting that *C. splendens* is capable of mounting robust immune responses to a variety of different pathogens and parasites.

To examine the PGRPs in detail, the regions corresponding only to the matched PGRP domains from each protein were first extracted. Initial phylogenetic analyses with RAxML on MAFFT amino acid sequence alignments clearly distinguished between ‘shared’ (more closely-related to those from other insects) and ‘specific’ (only found in *C. splendens*) domains. Thus, to estimate the PGRP-domain maximum likelihood phylogeny with domains from *C. splendens*, *D. melanogaster*, and *A. mellifera*, MAFFT alignments were first built for the shared and specific sets separately, and these alignments were then combined with MAFFT’s merge function. MAFFT alignments were performed with default parameters and RAxML phylogenies were built with 100 bootstrap replicates. To confirm that such multi-domain PGRP domains were indeed not previously found in any other known species,

InterPro matches against the UniProt Archive (UniParc) (UniProt Consortium 2015) (data from InterPro FTP site: uniparc_match.tar.gz file dated 02/17/16) were scanned for entries showing more than two PGRP (IPR006619) domains. The details of the twelve resulting matches, along with comments on the reliability of their annotation, are presented in table S8. Several matches were to proteins that have already been withdrawn (status inactive) and replaced with corrected annotations that no longer have multiple PGRP domains. For example, several fly proteins, each with three domains, are in fact incorrect gene annotations of PGRP-LC that have combined the three domains that in *D. melanogaster* are mutually exclusive alternative transcripts, and a 4-domain monkey protein results from an incorrect gene annotation that fuses the neighboring *PGLYRP3* and *PGLYRP4*. This comprehensive search revealed that no other organism with available protein sequences has genes annotated with more than four PGRP domains encoded by a single gene.

Chemoreceptors

Although odonates had long been considered to be largely anosmic, relying primarily on visual and tactile stimuli for feeding and mating (e.g. Corbet 1980; Crespo 2011), recent studies have revealed diverse chemosensory capabilities (Rebora et al. 2012, 2013, 2014; Piersanti et al. 2014a,b, 2016; Frati et al. 2015, 2016). Three large families of chemoreceptors mediate most of the specificity and sensitivity of olfaction and taste in insects, the Odorant Receptor and Gustatory Receptor families of seven-transmembrane ligand-gated ion channels (Benton 2015; Joseph and Carlson 2016), which are distantly related to each other in the insect chemoreceptor superfamily now known to be present even in basal animals (Robertson et al. 2003; Saina et al. 2015; Robertson 2015), and the unrelated three-transmembrane Ionotropic Receptors, which are variants of the ionotropic glutamate receptors also widespread in animals (Rytz et al. 2013). With the exception of two kinds of co-receptors (OrCo in the ORs and Ir8a, 25a, and 76b in the IRs) and a few other GRs and IRs, most of these receptors evolve rapidly and are highly divergent both across orders of insects and across each receptor family. Gene models for them are usually therefore not well built by genome-wide automated gene modeling, unless supported by deep transcriptome sequencing of relevant chemosensory and other tissues. Indeed, the automated annotation for *C. splendens* has only partial models for *OrCo*, the single sugar receptor *GR1*, the three *IR* co-receptors, and four more *IRs*.

We therefore undertook an exhaustive manual annotation of these three chemoreceptor families. TBLASTN searches with e-values up to 100,000 (Altschul et al. 1997; Camacho et al. 2009) with query proteins from the termite *Z. nevadensis*, a non-holometabolan insect with a complete manually annotated set of chemoreceptors (Terrapon et al. 2014), and many receptors from other insects, were performed on both the main genome assembly and the excluded short scaffold set. Gene models were built manually in the text editor TextWrangler, which accommodates up to 18kb of sequence on a single line, which is important because genes in large genomes commonly have long introns. Splice predictions were obtained from the Splice Prediction by Neural Network (Reese et al. 1997) webserver at the Berkeley Drosophila Genome Project (http://www.fruitfly.org/seq_tools/splice.html), although it does not recognize variant GC donor sites, five of which were invoked to build suitable models. Relevant gaps in the assembly were repaired using raw reads from the four 550bp shotgun libraries available in the Short Read Archive at NCBI, and many of them simply required

collapsing unresolved flanking direct repeats (negative gaps). Occasionally models were created that spanned two scaffolds, based on the appropriateness of connecting them across scaffolds. A few pseudogenes were included in the gene set, when they could be built to encode more than 50% of the family length without disrupting the alignments too badly, and were translated as best possible to encode an alignable protein (using Z for stop codons and X for all other pseudogenizing mutation such as frameshifting indels and mutated intron splice junctions). Iterative TBLASTN searches were performed with all newly identified chemoreceptors in an attempt to exhaustively find all members of each family. Gene models were refined in light of repeated multiple alignments of each family.

The final multiple alignments for each family, along with representative receptors from *Z. nevadensis* (removing all pseudogenes, most partial proteins, and some closely related ones), as well as *D. melanogaster* and other insects when relevant, were performed with CLUSTALX v2.1 (Larkin et al. 2007), which is particularly good at aligning these highly divergent proteins through alignment of their transmembrane domains. Poorly aligning and gapped regions were removed using Trimal v1.4 (Capella-Gutierrez et al. 2009) with the “gappyout” option. Maximum likelihood phylogenetic analysis for the trees presented in this supplementary were performed using RAxML v7.6.6 (Stamatakis 2006), and tree figures were prepared in Figtree v1.4.2 (http://tree.bio.ed.ac.uk/software/figtree/) and InkScape v0.91 (http://inkscape.org).

The Gustatory Receptor (GR) family

There are 51 *GR* genes in the *C. splendens* genome, coding for 115 proteins. It is hard to be confident that all of the divergent GRs in a genome have been identified, especially since there are no GRs from closely related insect species that could greatly increase the sensitivity of our searches. Moreover, the relatively well-conserved C-terminal region is split across three short exons, which makes it difficult to find divergent members of the family using TBLASTN searches. PSI-BLASTP searches of the automatically annotated proteins from a genome will sometimes reveal divergent GRs not identified by TBLASTN searches, however in this case only one of the identified GRs is partially annotated, so this is not a useful approach. We attempted to perform exhaustive TBLASTN searches using as query the protein sequences encoded by the last two exons of each identified GR with LQ before them (to represent the last six positions of a consensus splice acceptor) and VS after the penultimate one (to represent the first six positions of a consensus splice donor), with E=1,000,000, but found no additional candidate GRs. TBLASTN searches with E=100,000 with the 58 GRs from *D. pulex* also failed to identify any additional GRs. We therefore believe we have identified most, if not all, of the GRs in this genome, although many fragments, most recognizable as pseudogenic, related to the named genes remain and some might represent intact genes.

For the phylogenetic analysis we first tested inclusion of the GRs reported by Missbach et al. (2014) from their transcriptome analysis, however most are too short or align too poorly to be usefully included. We therefore included only the putative full-length GRs for the bristletail and firebrat. Representatives of the sugar receptor clade from *D. melanogaster* and *Z. nevadensis*, the carbon dioxide receptor clade from *Anopheles gambiae* and *Z. nevadensis*, and representative members of the fructose receptor clade from diverse insects were included. A large clade of intronless GRs from *Z. nevadensis* was excluded as *C. splendens*

has no close relatives of them. The tree was rooted with the sugar and carbon dioxide subfamilies because these are the most distinctive and conserved subfamilies in the insect GR family (Figure S7).

There is a single relative of the conserved subfamily of sugar receptors of insects, named CsplGr1, and it is the only GR for which there is at least a partial model in the automated gene set (CSPLE_14204). The *D. melanogaster* sugar receptors appear to function as dimers (Fujii et al. 2015), and all other examined insect genomes encode at least two candidate sugar receptors, e.g. AmelGr1/2 in the honey bee (Robertson & Wanner 2006) and ZnevGr1-6 in *Z. nevadensis* (Terrapon et al. 2014), so it is unclear how CsplGr1 in *C. splendens* might function as a sugar receptor. Freeman et al. (2014) reported that single sugar GRs expressed in an "empty" olfactory sensory neuron mediated appropriate responses to sugar, while Jung et al. (2015) found that AmelGr1 alone is responsive to sugars, but the dimer is more sensitive, so a single sugar GR could suffice. Missbach et al. (2014) found a member of this subfamily in the bristletail (LsigGr2) showing that the sugar receptor subfamily is at least that old, and the crustacean *D. pulex* also has members of it (Penalva-Arana et al. 2009), so it predates insect evolution.

CsplGr2 is similarly a single relative of the expanded subfamily of GRs related to the three carbon dioxide receptors of flies and some other Holometabola that also function as dimers (Robertson & Kent 2009), but this subfamily is expanded in the bedbug *Cimex lectularius* (Benoit et al. 2016) and in *Z. nevadensis* (Terrapon et al. 2014), and it is not clear what ligands they recognize. Therefore CsplGr2 cannot be designated as a carbon dioxide receptor, although we note that odonates are capable of detecting carbon dioxide, involving inhibition of olfactory sensory neurons in coeloconic sensilla (Piersanti et al. 2016), however this response might be mediated by ionotropic receptors (see below). Nevertheless, CsplGr2 clearly represents the GR lineage from which the sensitive carbon dioxide receptors of Holometabola evolved, and indicates the antiquity of this lineage in insects. Missbach et al. (2014) did not find a clearcut member of this subfamily in their three insects, but since they worked from transcriptomes, albeit deep ones, it is always possible that members of this subfamily reside in zygentomans and/or archaeognathans. The subfamily has not been detected outside of insects.

CsplGr3-6 are highly divergent proteins that were discovered as weak matches in searches with the DmGr43a protein and its relatives in other insects. This protein functions as a fructose receptor both in the sensory periphery and the brain of *Drosophila* (Miyamoto and Amrein 2014). It has multiple relatives in various other insects, but relatives were not identified in the termite (Terrapon et al. 2014). In the tree, along with TdomGr4, this clade clusters near, but not confidently with, the fructose receptor clade, so it remains uncertain where they and TdomGr4 represent the origins of this fructose receptor subfamily in basal insects (see also Figure 4 in Missbach et al. 2014). This subfamily has also not been detected outside of insects.

The remaining 108 GRs form an expanded and species-specific clade confidently related to the intron-containing divergent GRs of the termite (Figure S7). They have features common to most GRs in other insects and indeed other arthropods (e.g. Robertson et al. 2003;

Robertson 2015). First, their genes have the ancestral structure of a long first exon that encodes transmembrane domains 1-6 followed by three short exons separated by three phase-0 introns in locations shared across most GRs, encoding intracellular loop 3, transmembrane domain 7, and the extracellular C-terminus. There are only a few exceptions to this structure in that some genes have idiosyncratically acquired novel introns that interrupt this usually long first exon (Gr6 has a phase 2 intron, Gr10 a phase 0 intron, Gr11/12 independent phase 1 introns, Gr17 a phase 2 intron, and Gr29 a phase 0 intron, and importantly none of these genes are parts of the alternatively-spliced genes described below, where such an intron interrupting the first long exon would not be compatible with the alternatively-spliced model). Second, the transmembrane 7 domain includes the only reasonably well-conserved and signature region of the GRs (the 7_tm7 family in Conserved Protein searches at NCBI, which they all find), the TYhhhhhQF motif (where h is any hydrophobic amino acids), although commonly it is THhhhhhQF with a few more unusual modifications, e.g. ANhhhhhQF in Gr7. Third, many of them exhibit an unusual form of alternative splicing in which multiple first exons are spliced to a set of these final three exons, sometimes generating large numbers of GRs that share their C-termini but differ in transmembrane domains 1-6, implying that these mediate the specificity of ligand-binding (Gr4a/b is similarly modeled as being alternative-spliced). While we have no RNAseq data to support these alternatively-spliced models, they are so similar to ones highly supported in some other insects that they are clearcut. However, the fragmented nature of the genome assembly, perhaps sometimes caused by the similarity of many of these long first exons, resulted in several scaffolds containing only a few first exons. In each case, however, these could be confidently assigned to one of the alternatively-spliced loci. The largest of the alternatively-spliced loci is Gr51a-u, with 21 first exons (and many pseudogenic fragments, see below). Fourth, the five large alternatively-spliced loci (Gr47-51) largely form separate clusters in the tree, as expected since they presumably result from tandemly-arranged expansions of the first exon through unequal crossing-over, and share their C-terminal regions. Fifth, these alternatively-spliced loci contain several pseudogenic first exons, consistent with rapid ecologically-relevant evolution of chemoreceptors. Only those encoding at least 50% of a typical GR were included, and many fragmentary pseudogenic remnants are present in the five largest alternatively-splice loci, and a few elsewhere in the genome. Fifteen such pseudogenic constructs were included in the named protein set, so the number of potentially functional GRs is 100. It is worth noting that none of these pseudogenes had only a single stop codon, indeed most had multiple pseudogenizing mutations including frameshifts and intron boundary mutations, hence they could not be pseudo-pseudogenes (Prieto-Godino et al. 2016). While the functions of these GRs is unknown, they share the features of bitter taste receptors in other insects, and are presumably involved in mediating much of gustation in the contexts of feeding and oviposition in this damselfly.

The Odorant Receptor (OR) family

The OR family in most insects consists of a single highly conserved protein that serves as a co-receptor with all the remaining ORs, known as the Odorant receptor Co-receptor or OrCo. The remaining ORs are known as “specific” ORs as they confer the specificity of ligand-binding to the dimer. OrCo was partially modeled as CSPLE_00492, however no specific ORs were found in the automated models. Searches of the assembly with ORs from *Z. nevadensis* uncovered a single specific OR in the assembly, called CsplOr1, encoded by a 6-exon gene.

Searches of the short scaffolds excluded from the main assembly revealed at least three more genes split across multiple short scaffolds, which were successfully connected to yield three 6-exon genes (CsplOr2-4) with evidence that the latter two are in a tandem arrangement. CsplOr2-4 share 42-49% amino acid identity and have 25-27% identity with CsplOr1. There are multiple short scaffolds with sequences similar to parts of these ORs and examination of the read depth for these three genes indicates that they probably represent 2-3 closely-related genes each. Finally, a single severely degraded pseudogene distantly related to these was noted, but could not be built sufficiently to include in the analysis. These proteins are unequivocally members of the OR family because their gene structure is similar to other ORs, especially in sharing three phase-0 introns in the same locations near the C-terminus (see above for GRs too), and all recover the 7tm_6 family in the NCBI Conserved Protein search, which is the OR family in insects. They are nevertheless highly divergent, sharing just 25-27% identity over their C-terminal two thirds, with their closest OR relatives in the GenBank non-redundant protein database, while an iteration of PSI-BLASTP searches yields full-length alignments with ~20% identity. Thus *C. splendens* has at least four specific ORs. This low number of ORs is consistent with the known reduced olfactory capacities of odonates, but it remains unclear why they do not appear to have glomerular antennal lobes and mushroom body calyces usually involved in transmission of olfactory signals, structures present in the older firebrat (Farris 2005).

Robertson et al. (2003) speculated on the basis of a tree of the insect chemoreceptor superfamily of ORs and GRs from *D. melanogaster* that the OR family might have evolved from a lineage of GRs near the base of the Insecta, perhaps in conjunction with the evolution of terrestriality. Missbach et al. (2014), however, could not identify OrCo or specific ORs in extensive transcriptomes of a wingless archaeognathan, the bristletail *Lepismachilis y-signata*, but discovered three OrCo-like proteins, but no specific ORs in another wingless insect, the firebrat *Thermobia domestica* (Zygentoma), which most insect phylogenies indicate is a slightly more recent branch in the tree. They concluded that OrCo, at least, had evolved within insects, with specific ORs evolving after these wingless orders, perhaps by the Palaeoptera. Our finding of both a single OrCo and at least four specific ORs in this odonate indicates that the complete OrCo/OR system had indeed evolved by the time of the Palaeoptera.

To examine the relationships of these ORs further, our phylogenetic analysis included the three *T. domestica* OrCo (TdomOrCo) proteins, a representative set of OrCo proteins from other insects, three ORs from a phasmatodid *Phyllium siccifolium* also generated by Missbach et al. (2014), and a representative subset of the 69 ORs in *Z. nevadensis* (Terrapon et al. 2014). The OrCo proteins were declared the outgroup to root the analysis based on the intermediate position of this protein at the base of the OR family and nearer the GRs in analysis of the insect chemoreceptor superfamily (e.g. Robertson et al. 2003). The resultant tree shows the confident and phylogenetic appropriate clustering of the *C. splendens* OrCo, while the four specific ORs form a distinct and basal lineage relative to the termite and phasmatodid ORs (Figure S8), consistent with them representing early, specific ORs. It remains possible, however, that one or two of the TdomOrCo-like proteins, for example TdomOr1 and 3, in fact have evolved the role of a specific OR (Missbach et al. 2014).

The Ionotropic Receptor family

Twenty IRs are recognized and named. The partial models for the co-receptors Ir8a (CSPLE_01514), Ir25a (CSPLE_02687), and Ir76b (CSPLE_15032), as well as Ir40a (CSPLE_01471) and Ir75a-c (CSPLE_12959 and CSPLE_09716/8), were improved manually, although Ir8a, 25a, and 75c remain incomplete. New models were built for orthologs of Ir21a, 40a, 68a, and 93a, indicating that these are ancient genes in insects, although *Z. nevadensis* appears to have lost Ir40a. However, unlike the three co-receptors, with the exception of Ir93a in crustaceans (Corey et al. 2013; Groh-Lunow et al. 2015), they are not found in other arthropods (Chipman et al. 2014; Hoy et al. 2016; Gulia-Nuss et al. 2016). Ir21a, Ir40a, and Ir93a, along with Ir25a, have recently been shown to mediate sensing of temperature and humidity in *Drosophila* (Ni et al. 2015; Enjin et al. 2016; Knecht et al. 2016), while the role of Ir68a remains unclear.

Three relatives of the commonly-expanded Ir75 clade were also found, and this clade in *Drosophila* responds to various acids and amines (Silbering et al. 2011; Prieto-Godino et al. 2016). No convincing relatives were found for the pair of Ir41a/76a in *D. melanogaster* that are also commonly expanded in other insects. No clear orthologs for any other of the 60 *D. melanogaster* IRs were discovered, and specifically no relative of Ir64a, which is implicated in perception of high concentrations of carbon dioxide (Ai et al. 2010). Many insects and other arthropods examined from full genome sequences also have highly divergent IRs in two distinct sets, those with a set of introns roughly comparable to those of the above IRs, and an “intronless” set (a few of which have idiosyncratic newly-acquired introns). Following an approach used for *Z. nevadensis* (Terrapon et al. 2014) and other arthropods (e.g. Hoy et al. 2016) these were numbered from Ir101, avoiding any confusion of possible orthology with the D. melanogaster IRs, which were named for their cytological locations and only go up to Ir100a. These intron-containing genes are particularly hard to model as they are so divergent, and just five are included here (Ir101-105), although there are fragments of more of them, plus a set of fragmentary pseudogenes that might be remnants of a once-expanded clade. Only five “intronless” genes were found (Ir106-110), although Ir110 has acquired two novel introns.

The IR phylogenetic analysis included representative IRs from *Z. nevadensis*, a few from *D. melanogaster*, and several from each of the three insects in Missbach et al. (2014), including several of their partial sequences because they usually include the more conserved C-terminal regions, facilitating alignment and phylogenetic analysis. It was rooted with the Ir8a and 25a proteins, which in larger analyses including the ionotropic glutamate receptors from which the IRs evolved, clearly cluster with them (Terrapon et al. 2014; Missbach et al. 2014) (Figure S10). It reveals the orthology of Ir8a, 21a, 25a, 40a, 68a, and 93a, as well as the Ir75a-c set. Inclusion of the IRs from Missbach et al. (2014) reveals that the IR75 clade is even older than paleopterans because their LsigIr9 belongs in it. Interestingly, among the divergent IRs the intron-containing Ir105 is closely related to the intronless Ir106/107, indicating a recent loss of its five introns from a common ancestor, presumably through recombination with a cDNA copy. In contrast, Ir108-110 cluster confidently in the clade of intronless termite IRs, indicating a far more ancient loss of their introns (see Terrapon et al. 2014).

Ionotropic receptors have been implicated in both olfaction and gustation in *D. melanogaster* (Rytz et al. 2013; Koh et al. 2014; Stewart et al. 2015), and some are even involved in detection of other stimuli like temperature and humidity (Ni et al. 2016; Enjin et al. 2016; Knecht et al. 2016). It is remarkable that in addition to Ir93a the three conserved co-receptors, Ir21a, 40a, 68a and the Ir75 clade are present in this palaeopteran, indicating that they are at least this old in the insect lineage, with the IR75 clade being even older. It remains unclear what role the divergent Ir101-110 play in odonate chemosensation as they have only distant relationships with either the “antennal” or “divergent” IRs recognized in *Drosophila* which generally are involved in olfaction and gustation, respectively (Rytz et al. 2013; Koh et al. 2014; Stewart et al. 2015).

Conclusion

We describe a complete set of OrCo/OR proteins for a paleopteran insect, showing that this central system of insect olfaction had evolved by then. It remains to be seen from genome sequences of the other major lineage of paleopterans, the mayflies, whether they too have this set, and genome sequence for zygentomans and archaeognathans are also required before one can confidently conclude they do not have the complete system (Missbach et al. 2014). We note that from a deep transcriptome of the damselfly *Ischnura elegans* generated from head, thorax, and abdomen, Chauhan et al. (2014) claimed to have found a single “Odorant receptor 2t1-like”, however they do not provide a sequence for it, and the “Most Similar Locus” they note (LOC101663266) was from a small Madagascar hedgehog and has been removed from GenBank.

The GR family that mediates much of insect olfaction is well represented in this damselfly genome, with a candidate sugar receptor, a receptor related to the carbon dioxide receptors of holometabolous insects, and 98 other apparently functional GRs including five that might be related to the fructose receptor of *Drosophila* and other insects. The remaining GRs are commonly encoded by large alternatively-spliced loci of the kind found in many other insects. Chauhan et al. (2014) reported seven GRs in their transcriptome, with similarities to Gr2a and 43a of *D. melanogaster* and other GRs in aphids and beetles, however we find no such convincing relationships to these receptors, and again their sequences do not appear to be publicly available. These are presumably fragments of GRs that have best matches to these other insect GRs, and probably are in fact related to the divergent GRs we describe.

The IR family has several highly conserved members, and in addition to the three co-receptors Ir8a, 25a, and 76b found widely in insects and beyond, we report Ir93a, which is a hygroreceptor also found beyond insects, and Ir21a, 40a, 68a, and a 75a-related clade for the first time in a palaeopteran. Ir21a and 40a are involved in thermoperception, however the combination of Ir8a and Ir75a-like proteins is likely to mediate olfactory perception of acids and amines, and some of the other divergent Ir101-110 might also be involved in olfaction, although IRs are also involved in gustation.

We therefore provide evidence for the likely molecular underpinnings of the emerging understanding that odonates are capable of olfaction in several ecologically relevant contexts (Rebora et al. 2012; Piersanti et al. 2014a; Piersanti et al. 2014b; Piersanti et al. 2016; Frati et al. 2015; Frati et al. 2016), and surely also gustation in both the context of feeding (Rebora et

al. 2014) and oviposition (Rebora et al. 2013), and that these sensory modalities are important to their biology.

Odorant binding proteins (OBPs)

OBPs are small proteins expressed by support cells at the base of chemosensory sensilla, and secreted into the sensillar lymph where they are believed to bind and transport odorants from the atmosphere to chemoreceptors in the membranes of the dendrites of chemosensory neurons (Pelosi et al. 2006; Pelosi et al. 2014). They usually have six conserved cysteines (Classic OBPs) that form three disulphide bonds maintaining their globular shape with a binding pocket in the extra-cellular environment, although some have lost two of these cysteines and one of the disulphide bonds (Minus-C OBPs), while others have gained two more cysteines that are presumed to form a novel disulphide bonds (Plus-C OBPs). While most are expressed in antennae, some are more widely expressed. Genome sequences and antennal transcriptomes have revealed tens of OBPs in most examined insects, and the gene family is present back to basal Hexapoda such as Collembola (Vieira and Rozas 2011; Pelosi et al. 2014; Missbach et al. 2015; P. Engsontia and H. M. Robertson, unpublished data). Unfortunately most OBPs are rapidly evolving and highly divergent proteins, and are commonly encoded by 5-8 short exons, so they are difficult to find using TBLASTN searches of genome sequences. Fortunately they are highly expressed, so most are recovered as full-length transcripts in antennal transcriptomes, and even whole body transcriptomes commonly contain at least partial transcripts, which are far easier to detect in TBLASTN searches because of their contiguity.

We nevertheless first searched the genome using TBLASTN with Evalue=100,000 with all available OBPs from the most closely related available neopteran insects, the termite *Z. nevadensis* with 29 (Terrapon et al. 2014), the cockroach *Blattella germanica* with 48 (Niu et al. 2016), and the wingless firebrat *T. domestica* with 32 and bristletail *L. y-signata* with 40 (Missbach et al. 2015). This search revealed a single OBP, CsplOBP1, with ~50% identity for the mature protein region to TdomOBP1, ZnevOBP22, and BgerOBP38, and 30% identity to LsigOBP1, which are conserved orthologs of DmelOBP73a (Missbach et al. 2015; Niu et al. 2016). Although they cluster with Classic OBPs, these proteins have eight conserved cysteines, one more N-terminal and one more C-terminal than the conserved six cysteines, which is different from the Plus-C OBPs (see Missbach et al. 2015). All but the first exon of this 7-exon gene are supported by multiple reads from the 1KITE transcriptome for *C. splendens* (Misof et al. 2014), and the first exon was identified by similarity to orthologs in other damselflies identified below.

No other OBPs were identified this way, so we similarly searched the assembled transcriptomes of two coenagrionid damselflies, *I. elegans* (Chauhan et al. 2014) and *C. puella* (Johnston and Rolff 2013). Chauhan et al. (2014) found a fragment of one OBP, but do not provide its sequence. We identified highly conserved homologs of CsplOBP1 in both species as well as three more full-length OBPs from each species that allowed us to partially model their orthologs in *C. splendens* (CsplOBP2-4). The first exon of OBP genes typically encodes the signal sequence, and is followed by a phase 0 intron, and in this large genome with large genes and long introns, could easily be many kb upstream (for example, it is 23kb

upstream for OBP1). It is therefore difficult to discover the first exon, and we failed for CsplOBP3 and 4, however it was identified using a single RNAseq read for CsplOBP2. The final short exon could not be identified confidently for CsplOBP2, however. These are Classic OBPs with six conserved cysteines, but are highly divergent from all OBPs in the four species above. They are encoded by six-exon genes with intron phases 0-1-0-1-0 (typical for OBPs and largely shared with OBP1, although OBP3 appears to have lost the final short divergent exon). CsplOBP2/3 are in a tandem arrangement and all three proteins share ~30% amino acid identity.

It is difficult to be certain we have identified all the OBPs in *C. splendens* because any OBP that is too divergent to find with TBLASTN in the genome sequence or expressed at too low a level to be found in the whole body transcriptomes of the two coenagrionid damselflies would not be discovered. Nevertheless the unusually small number of OBPs we found, an order of magnitude lower than many studied insects, is consistent with the small number of ORs.

Opsins

The *C. splendens* gene set was also searched for opsins using a set of 16 reference opsins (table S2) (Hering and Mayer 2014). All *C. splendens* genes that had a match with an e-value <1e-10 were retained as candidate opsins. In addition, HMM profiles were generated for each of the nine major opsin clades; cnidopsins, vertebrate c-opsins, pteropsins, Group 4 opsins, arthropsins, melanopsins, non-arthropod r-opsins, arthropod visual opsins and onychopsins (table S2) (Hering et al. 2012). These profiles were used to also scan the *C. splendens* gene set and genes having a match with an e-value <1e-30 were also retained as candidate opsins. The two candidate opsin sets were then merged, giving 34 candidate opsins, which were further examined for (a) existence of the conserved retinal-binding K296 residue (Palczewski et al. 2000), and (b) whether they had a significant match against an opsin-related cluster in Uniref50. Unless the candidate opsin contained at least one of the above, it was discarded. This filtering step resulted in a final set of 17 genes that could likely represent real opsins. As a last step before the phylogenetic analysis, all 17 opsins were manually curated using WebApollo (Lee et al. 2013). Additionally, we extracted the partial sequence of another two opsins from the genome sequence. These opsins were not present in the predicted gene set and belong to two separate groups: RGR-like opsins and arthropsins. Finally, we compared these 19 banded demoiselle opsins to the opsins that were recently identified in another three damselflies and ten dragonflies (Futahashi et al. 2015). It should be noted that six of the opsins were basal to every other opsin in the analysis and also the branches leading to them were very long. Apparently, they are distantly related to opsins, but do not represent real opsins. In agreement with this result, none of them contained the K296 conserved residue, which is present in all other damselfly opsins. As a result, we repeated the phylogenetic analysis without these six genes, in order to avoid errors, such as long branch attraction. For the phylogenetic analysis, we applied the same methods as for chemoreceptors: MAFFT, Trimal. RAxML, EvolView and Inkscape v0.91.

Arrestins

One of the protein families in our blastclust clusters (see "Protein families"), with similarity to arrestins, had at least twice as many proteins in the damselfly genome compared to any other insect genome. In an attempt to better study this family, we first obtained additional genes from the InterProScan analysis, by fetching entries matching the keyword "arrestin". The results encompassed all genes found in the arrestin blastclust cluster and four extra matches, for a total of 14 damselfly arrestins. We then examined the amino acid sequences to verify that the corresponding gene models were not fragmented, by looking for the presence of complete C- or N-terminal arrestin domains (NCBI conserved domain online, last accessed April 2016). Subsequently, the amino acid sequences encoded by these genes were extracted and compared with those corresponding sequences of 18 *D. melanogaster* arrestins. We restricted our analysis to only the fruit fly arrestins because only these are well-annotated. Phylogenetic analysis was performed as before (see above) using MAFFT, Trimal, RAxML, Evolview, and Inkscape.

B) Supplementary figures and tables

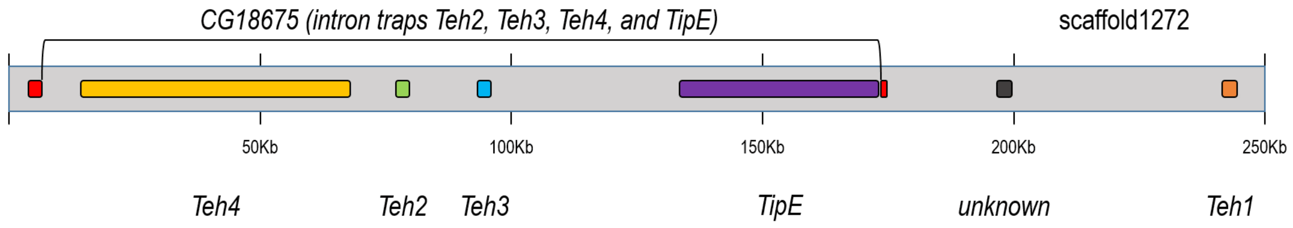

Figure S1. Conserved genomic arrangement of the *TipE* gene cluster in *Calopteryx splendens*. The last intron of the ortholog of *Drosophila melanogaster CG18675* gene traps the *Teh2*, *Teh3*, *Teh4*, and *TipE* orthologs, and the *Teh1* ortholog is located about 75Kb further downstream, matching the inferred ancestral arrangement of these genes in insects (Li et al. 2011). As in other insects, the last exon of *TipE* and of *CG1867* have a short sequence region in common, although in different reading frames.

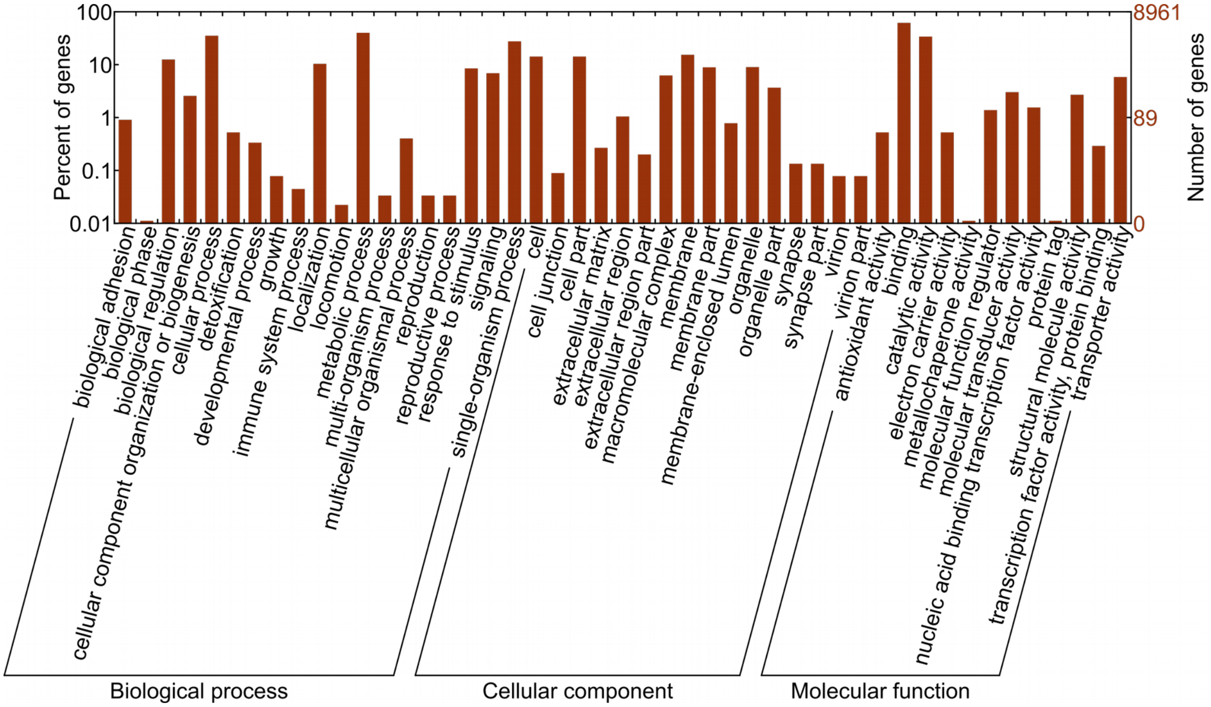

Figure S2: Number of *Calopteryx splendens* genes present in each GO functional category.

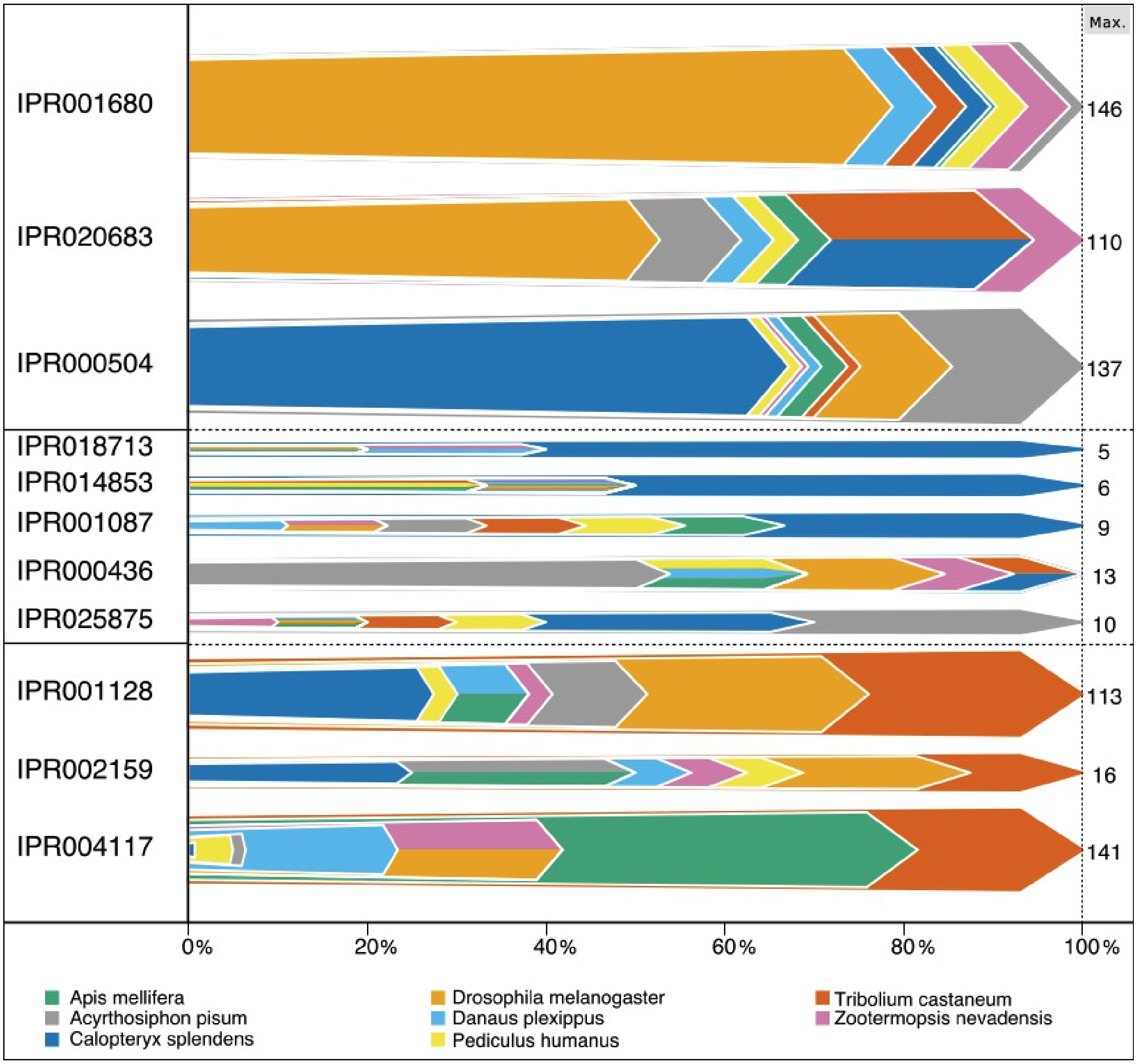

Figure S3: Comparison between eight insect species of InterPro entries of interest. The corresponding Pfam model is covered >75%. In the cases presented here, the three most abundant InterPro entries in the *Calopteryx splendens* gene set are shown, in comparison to other species (upper section). Cases in which *C. splendens* has a number of genes that is either above-average or below-average, are shown in the middle and lower section, respectively. Transposable elements were not considered. The value indicated on the right corresponds to the number of genes found in the species having the highest, and defining 100% on the scale. The height of each bar is proportional to its total gene count, following a square root scale. The description of each InterPro entry shown is: IPR001680 – WD40 repeat; IPR020683 – Ankyrin repeat-containing domain; IPR000504 – RNA recognition motif domain; IPR018713 – Domain of unknown function DUF2236; IPR014853 – Uncharacterized domain, cysteine-rich; IPR001087 – GDSL lipase/esterase; IPR000436 – Sushi/SCR/CCP domain; IPR025875 – Leucine rich repeat 4; IPR001128 – Cytochrome P450; IPR002159 – CD36 family; IPR004117 – Olfactory receptor, *Drosophila*.

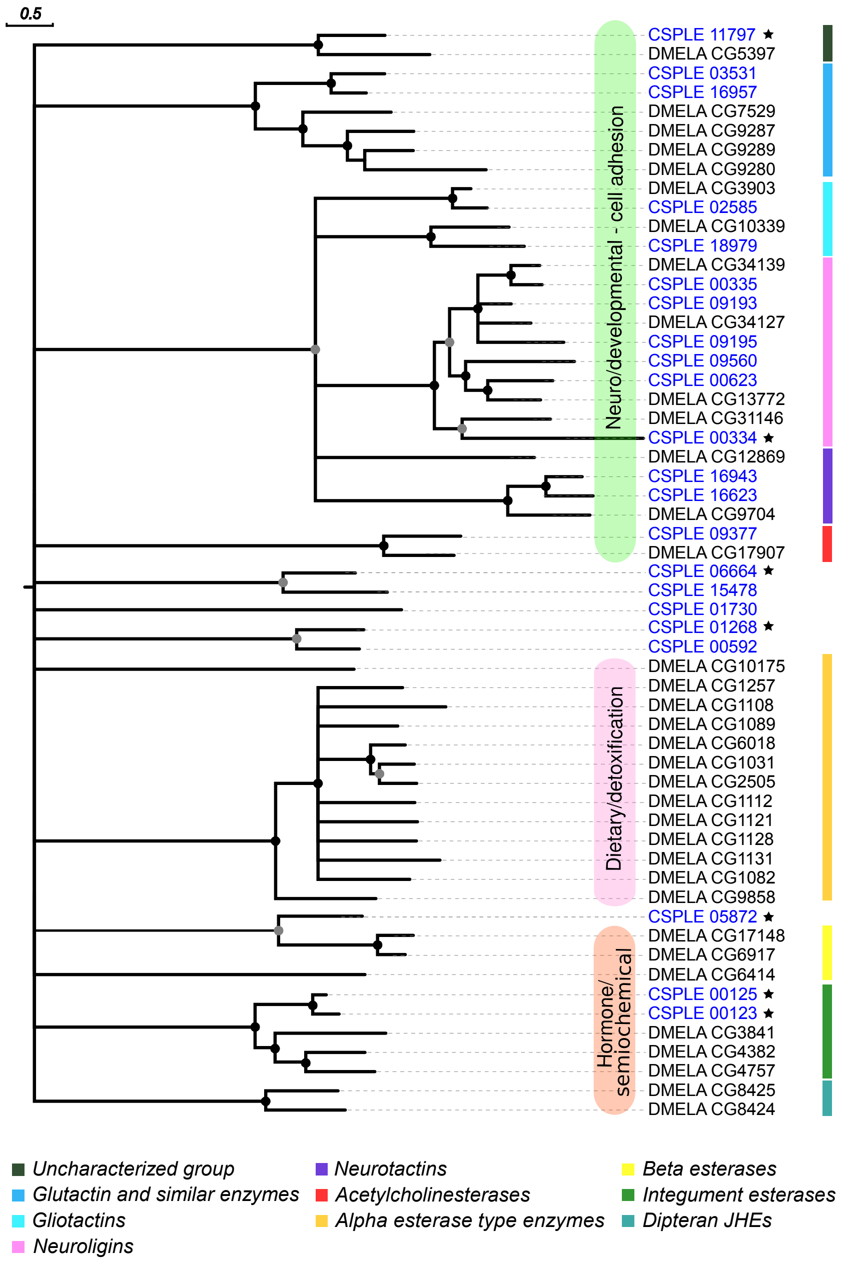

Figure S4: Maximum likelihood phylogenetic tree of CCE amino acid sequences from *Calopteryx splendens* (CSPLE, in blue) and *Drosophila melanogaster* (DMELA, in black). The tree was rooted with the human (HSAPI) AADAC gene as an outgroup. Nodes with >50% bootstrap support (100 replicates) are indicated with gray circles and nodes with >75% with black circles. Nodes with <50% support were collapsed. Stars indicate transcript evidence for the *C. splendens* CCEs. The functional assignment of clades follows the new system proposed by Oakeshott et al. (2010). The scale bar is in substitutions per site.

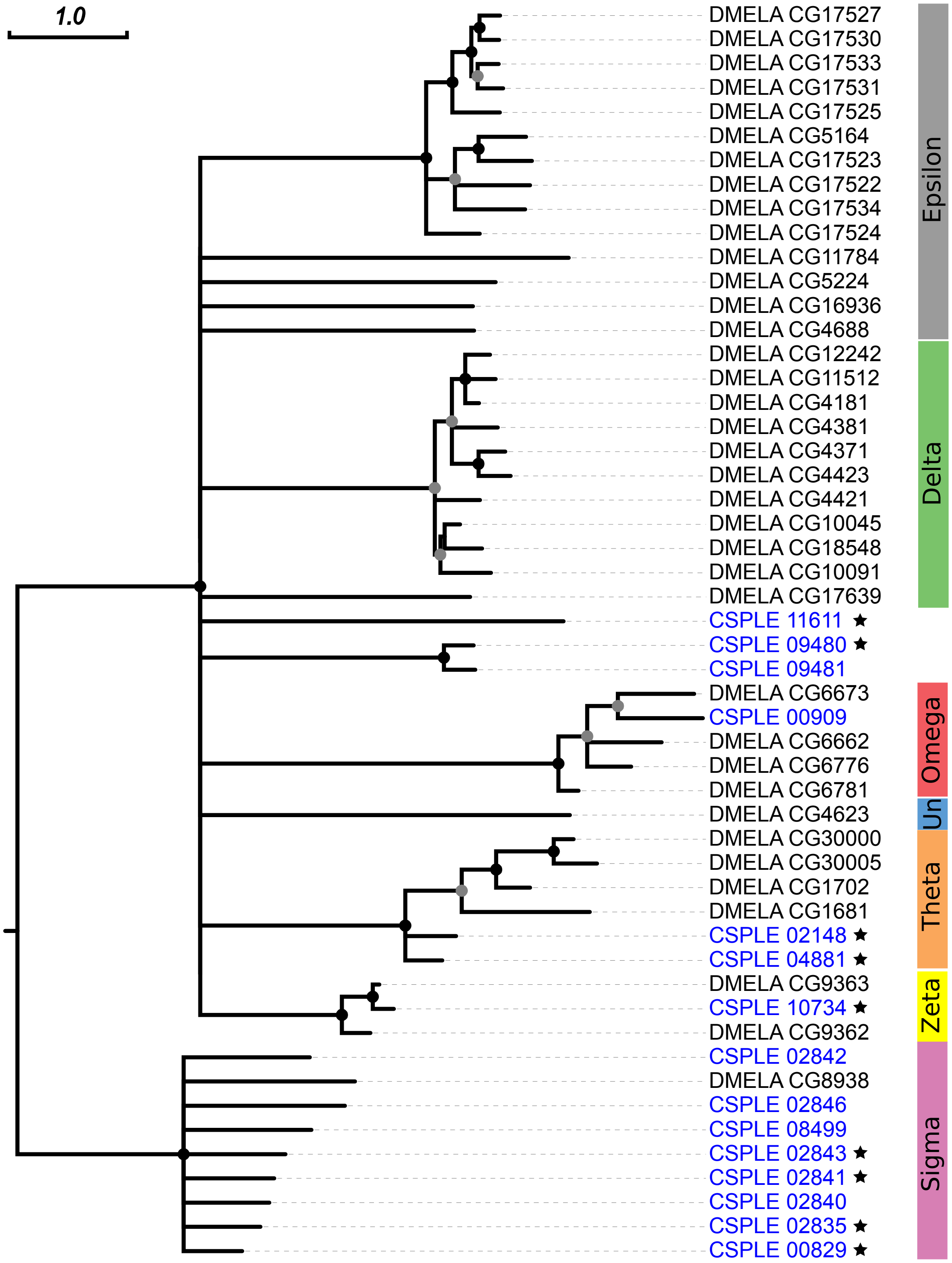

Figure S5: Maximum likelihood phylogenetic tree of GST amino acid sequences from *Calopteryx splendens* (CSPLE, in blue) and *Drosophila melanogaster* (DMELA, in black). The tree was rooted with the human (HSAPI) GSTA1 gene as an outgroup. Nodes with >50% bootstrap support (100 replicates) are indicated with gray circles and nodes with >75% support with black. Nodes with <50% support were collapsed into multifurcating nodes. Stars indicate transcript evidence for the *C. splendens* GSTs. The scale bar is in substitutions per site.

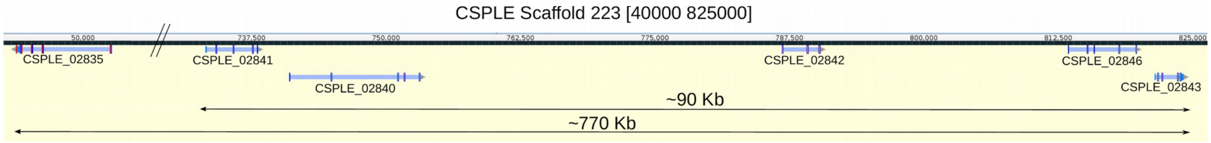

Figure S6: Genomic region encoding a cluster of six sigma *GST* genes in the *Calopteryx splendens* genome. Large boxes correspond to exons and arrows indicate gene orientation.

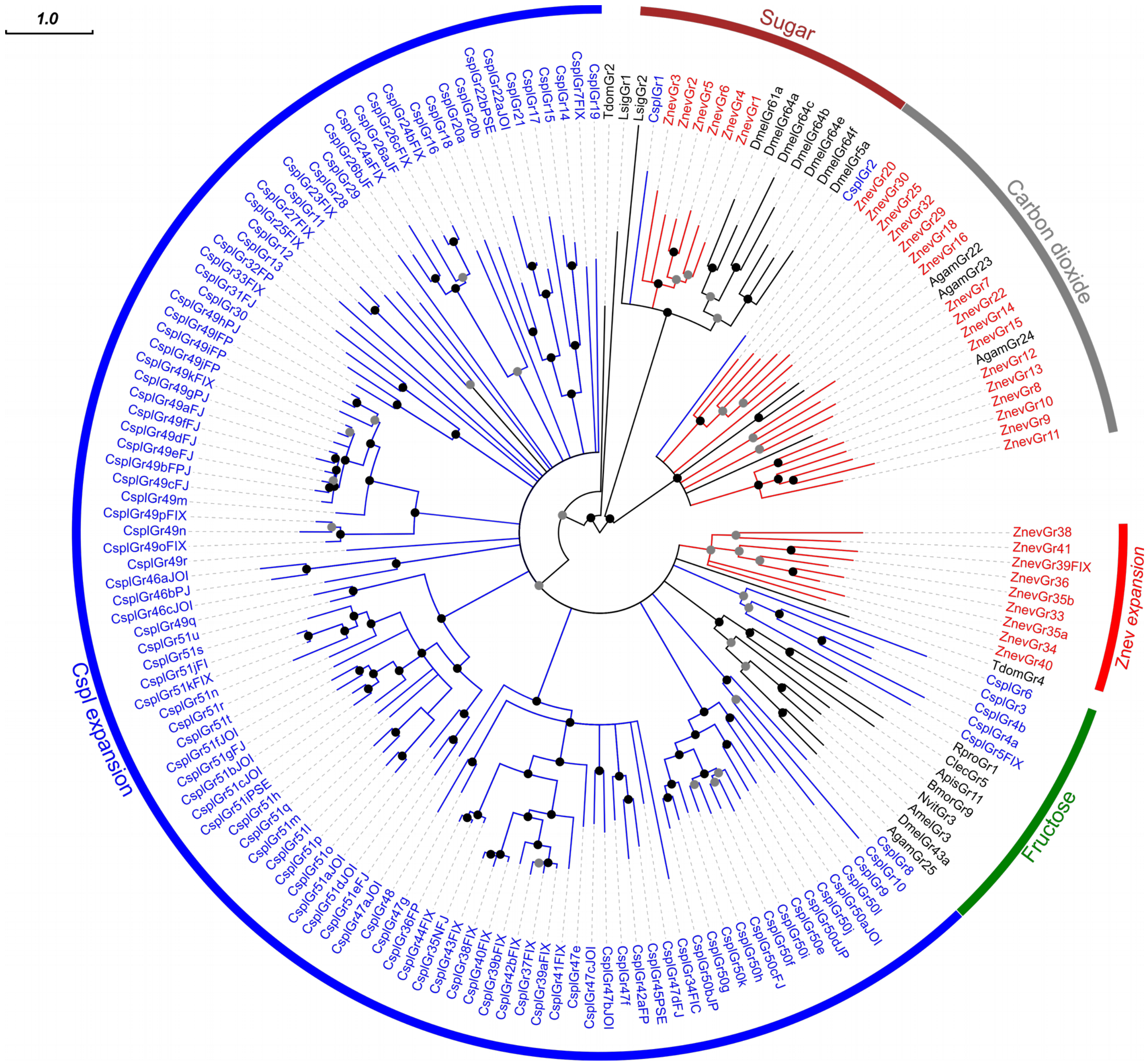

Figure S7: Phylogenetic analysis of the 115 identified *Calopteryx splendens* gustatory receptors (GRs). *C. splendens* genes appear in blue, and the termite *Zootermopsis nevadensis* genes appear in red. Nodes with >50% bootstrap support (100 replicates) are indicated with gray circles and nodes with >75% support with black. Nodes with <50% support are collapsed into multifurcating nodes. The scale bar is in substitutions per site.

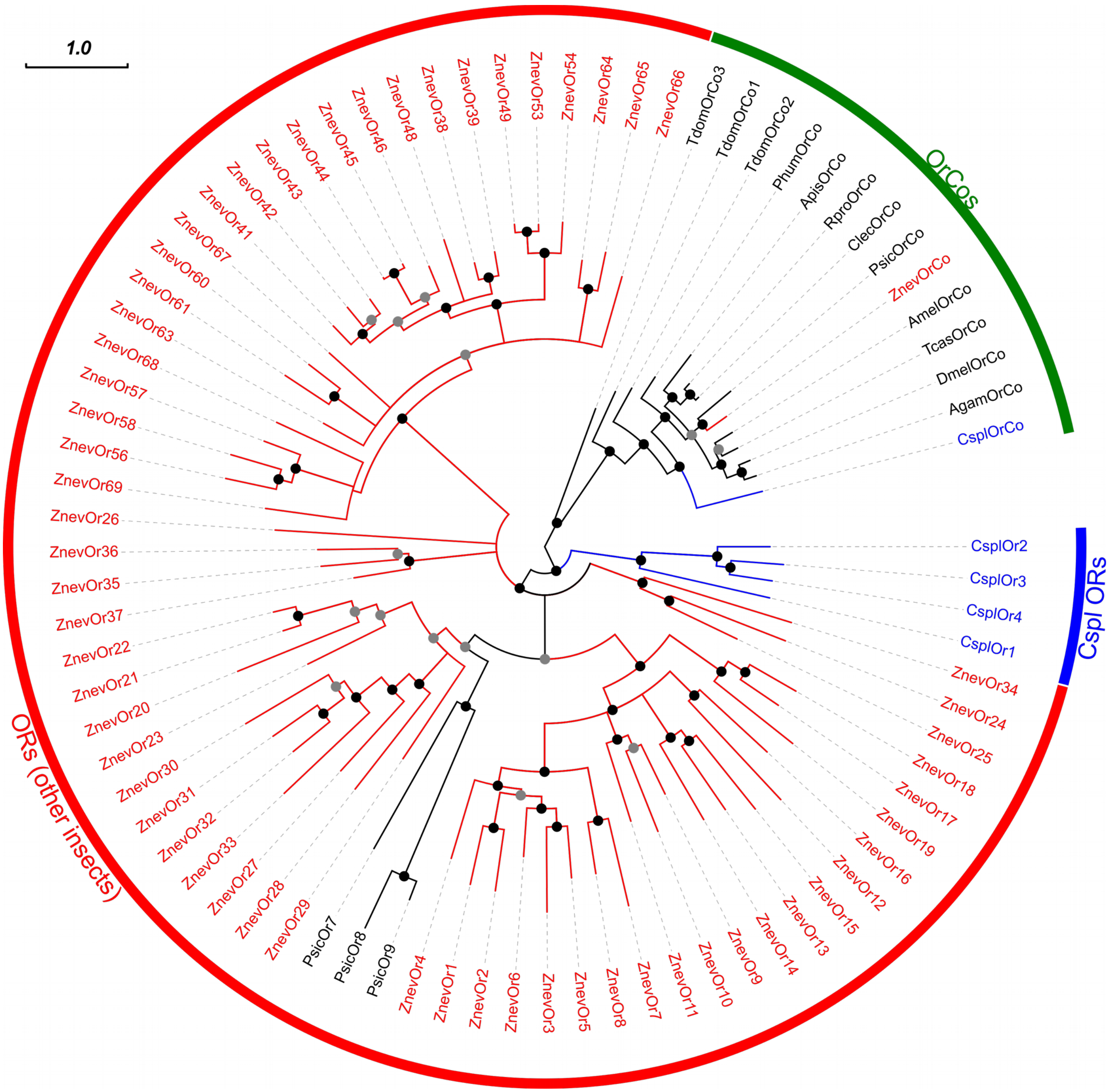

Figure S8: Phylogenetic analysis of the odorant receptors found in the *Calopteryx splendens* genome. *C. splendens* genes appear in blue, and the termite *Zootermopsis nevadensis* genes appear in red. Nodes with >50% bootstrap support (100 replicates) are indicated with gray circles and nodes with >75% support with black. Nodes with <50% support are collapsed into multifurcating nodes. The scale bar is in substitutions per site.

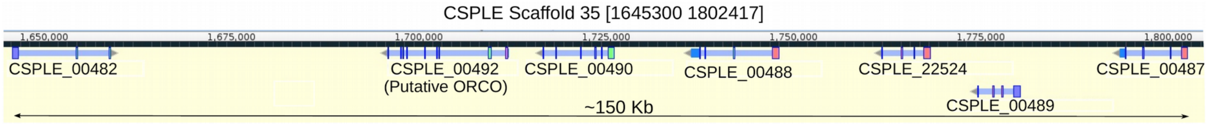

Figure S9: Genomic region encoding the putative *Calopteryx splendens Orco* gene (CSPLE_00492) inside a cluster of six *CYP* genes. Large boxes correspond to exons, while shorter boxes near the 3' end of a gene correspond to 3 UTRs (untranslated regions). Also, the arrows at each gene indicate whether the gene is encoded by the forward or the reverse strand.

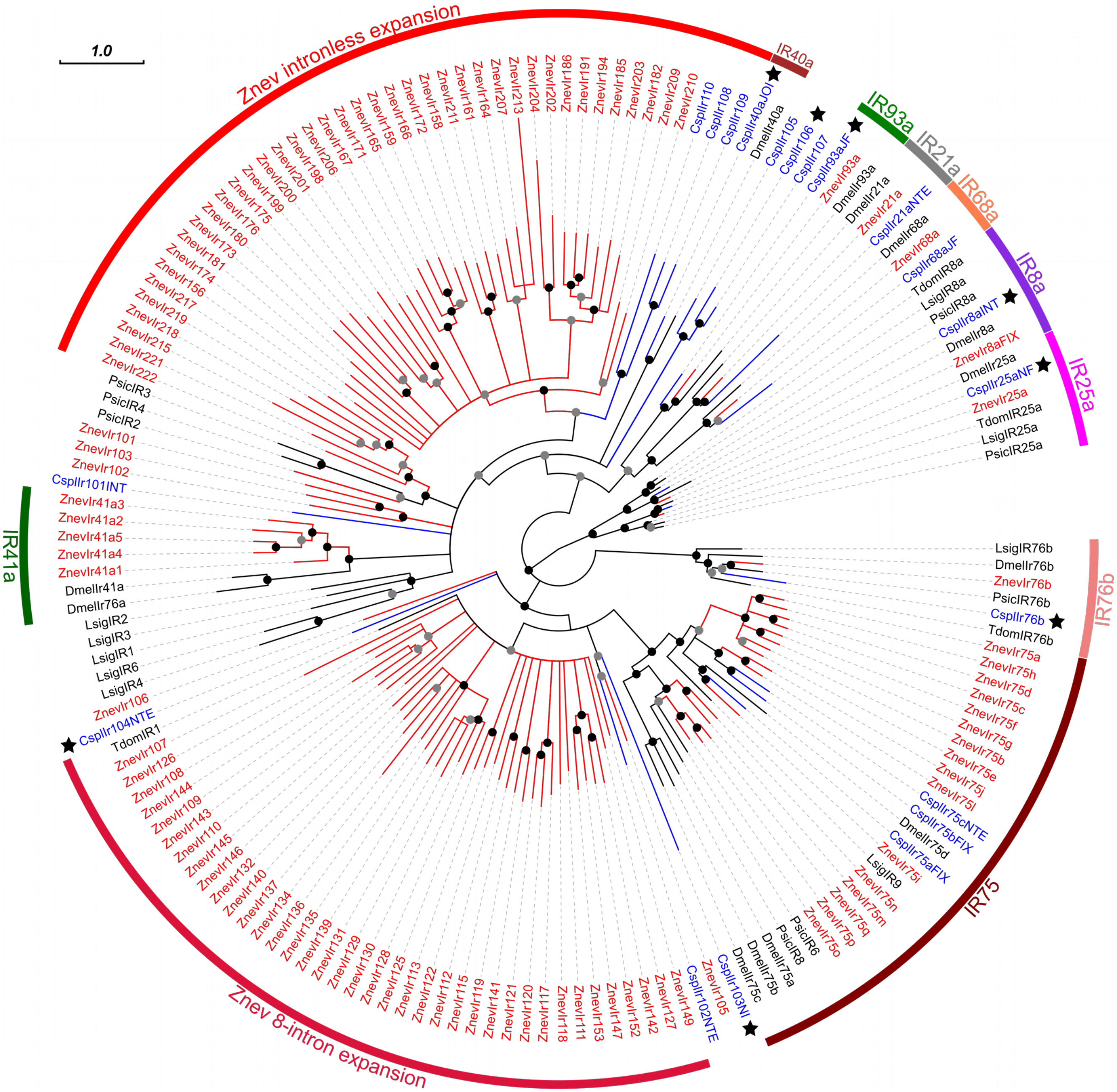

Figure S10: Phylogenetic analysis of ionotropic receptors (IRs) found in the *Calopteryx splendens* genome sequence. *C. splendens* genes appear in blue, and the termite *Zootermopsis nevadensis* genes appear in red. Stars indicate damselfly IRs for which there are matching transcripts in the 1KITE transcriptome. Nodes with >50% bootstrap support (100 replicates) are indicated with gray circles and nodes with >75% support with black. Nodes with <50% support are collapsed into multifurcating nodes. The scale bar is in substitutions per site.

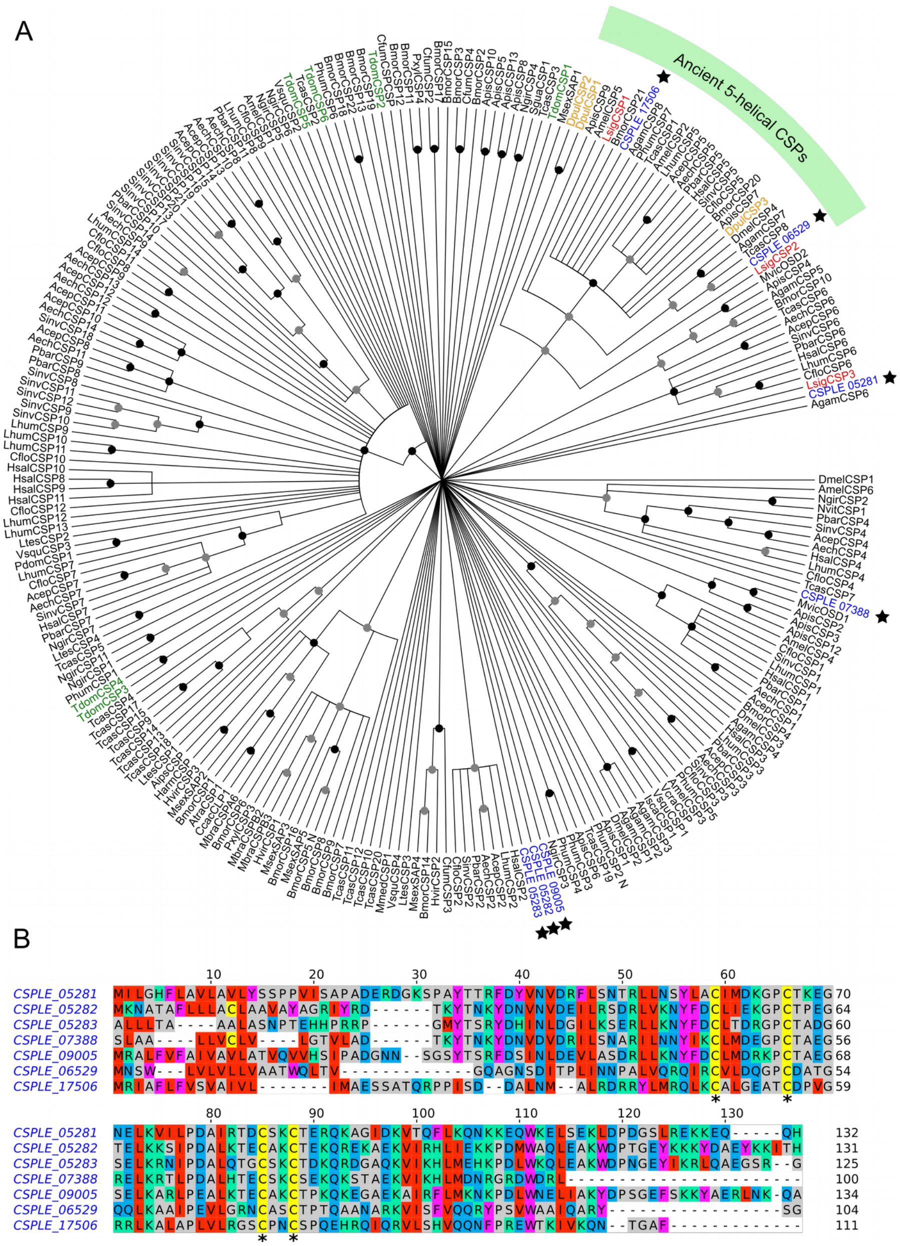

Figure S11: *Calopteryx splendens* chemosensory proteins (CSPs). (A) Maximum likelihood cladogram displaying arthropod CSPs. Different colors highlight CSPs from different species belonging in early-diverged insect lineages and the crustacean *Daphnia pulex*. Proteins from *C. splendens* are shown in blue, from *Thermobia domestica* in green, from *Lepismachilis y-signata* in red, and from *Daphnia pulex* in orange. The ancient, 5-helical CSP group is labeled in light green. Nodes with >50% bootstrap support (100 replicates) are indicated with gray circles while nodes with >75% support with black circles. Nodes with <50% support are collapsed. Transcribed *C. splendens* genes are indicated with stars. CSP amino acid sequences were taken from Kulmuni and Havukainen (2013) and Missbach et al. (2015). (B) Sequence alignment of *C. splendens* CSPs represented in the tree showing the highly conserved pattern of cysteines.

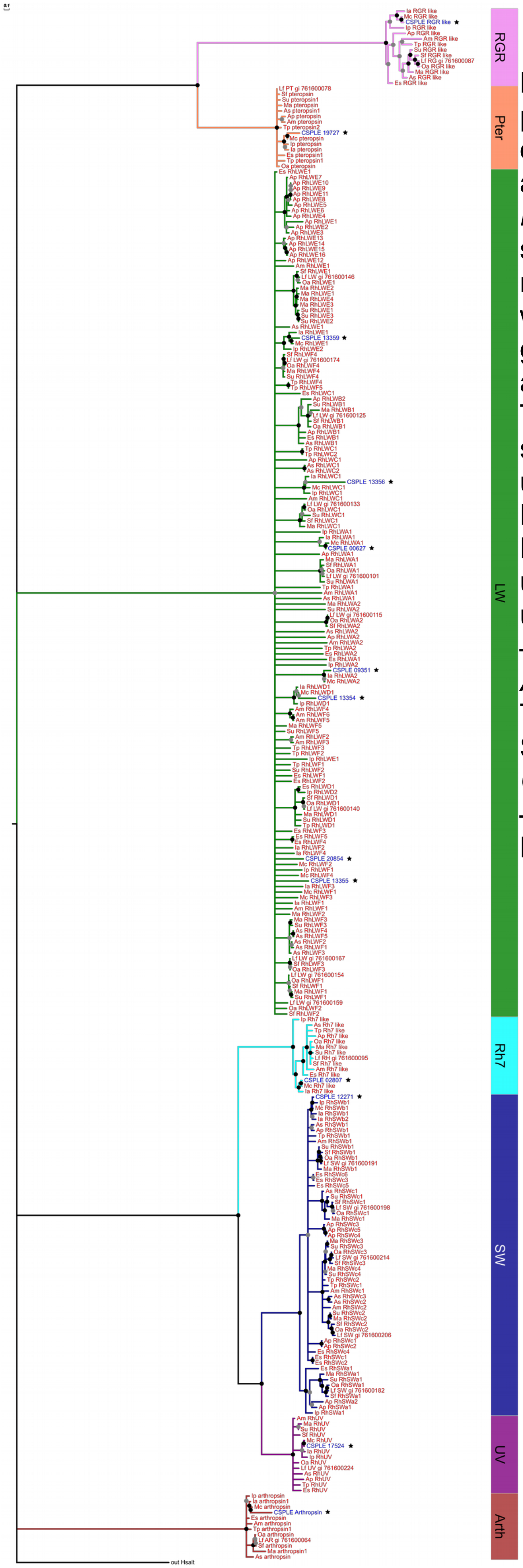

Figure S12: Phylogenetic analysis of the opsin proteins in *Calopteryx splendens* (names in blue) and other Odonata (names in red). The tree is rooted with an ancestral GPCR protein from the ant *Harpegnathos saltator*. Nodes with a bootstrap support >50% are marked with gray circles, while nodes with a support >75% with black circles. Nodes with <50% support were collapsed. Different opsin groups are indicated with a label on the right as well as with differently colored branches leading to them. Transcribed genes are indicated with a star. The scale bar is in substitutions per site. Abbreviations used for opsin groups: RGR – RGR-like; Pter – Pteropsins; LW – long wavelength-sensitive; Rh7 – Rh7-like; SW – short wavelength-sensitive; UV – ultraviolet-sensitive; Arth – Arthropsin. Abbreviations used for odonate species: Ia – *Ischnura asiatica*; Mc – *Mnais costalis*; Ip – *Indolestes peregrinus*; Ap – *Anax parthenope*; Am – *Asiagomphus melaenops*; Tp – *Tanypteryx pryeri*; Su – *Somatochlora uchidai*; Sf – *Sympetrum frequens*; Lf – *Ladona fulva*; Oa – *Orthetrum albistylum*; Ma – *Macromia amphigena*; As – *Anotogaster sieboldii*; Es – *Epiophlebia superstes*. Branch length scale is in substitutions per site.

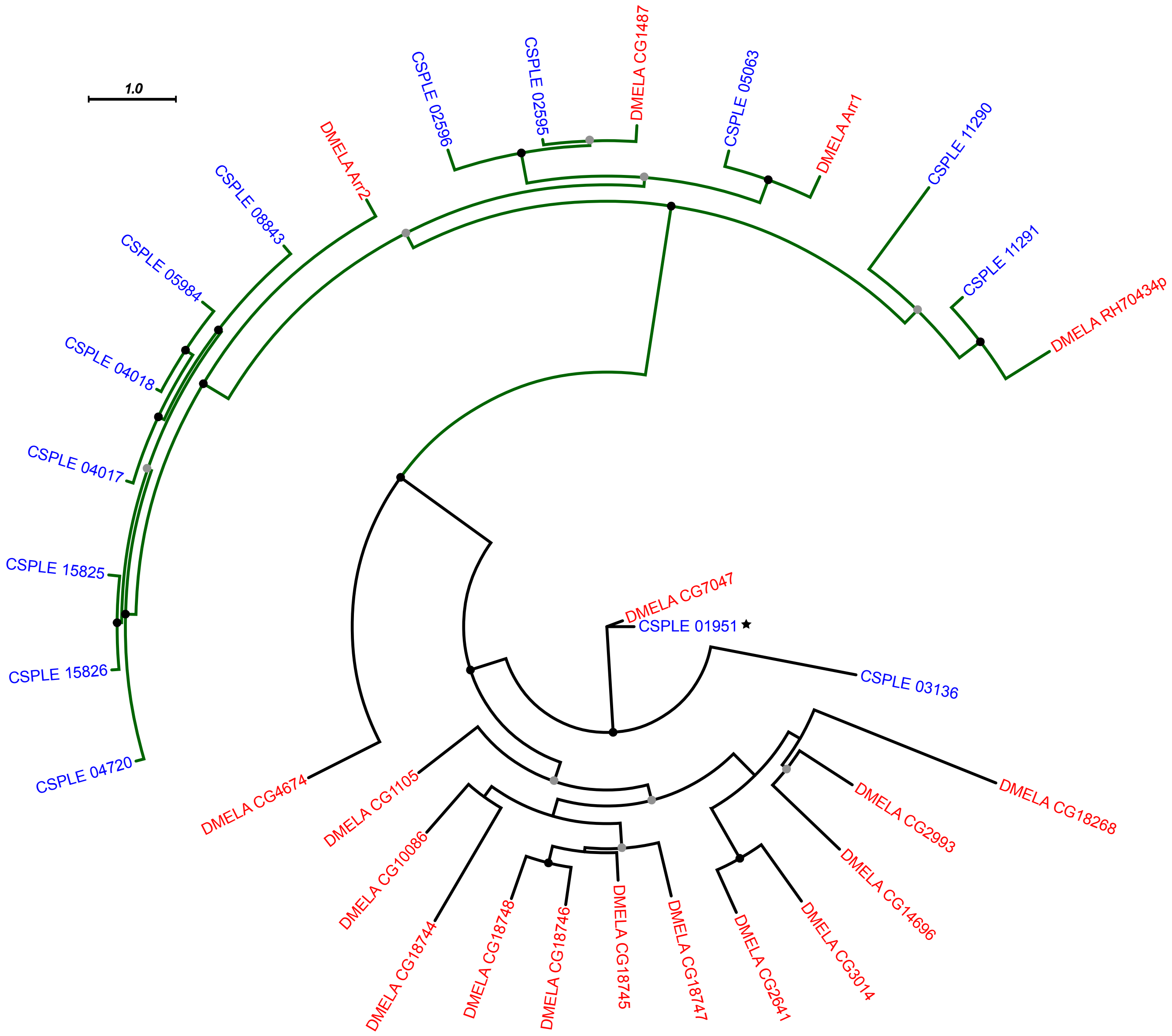

Figure S13: Phylogenetic analysis of *Calopteryx splendens* arrestins (names in blue), in comparison to those of *Drosophila melanogaster* (names in red). Twelve of the damselfly arrestins cluster with the four fruit fly β-arrestins (Arr1, Arr2, CG1487 and RH70434), thus representing an expansion of this particular group of arrestins in damselflies (branches shown in green). Nodes with a bootstrap support >50% are marked with gray circles, while nodes with a support >75% with black circles. The scale bar is in substitutions per site.

Table S1: Comparison of detoxification enzymes in different insect species.

		CS	DM	TC	AM	AP	BM
**CYPs**							
	CYP2 Clan	20	6	8	8	10	10
	CYP3 Clan	18	36	72	28	33	36
	CYP4 Clan	8	33	44	4	32	32
	Mitochondrial Clan	9	11	9	6	8	8
	CYP20 Clan	1	0	0	0	0	0
	Total	56	86	133	46	83	86
**CCEs**							
	**Dietary/detoxification class**						
	A clade	0	0	0	5	5	42
	B clade	0	13	14	3	0	13
	C clade	0	0	12	0	0	0
	**Hormone/semiochemical processing class**						
	D clade (integument esterases)	2	3	2	1	0	4
	E clade (beta esterases)	1	3	7	2	18	2
	F clade (dipteran JHEs)	0	2	2	2	0	0
	G clade (lepidopteran JHEs)	0	0	0	0	0	2
	**Neuro/developmental class**						
	H clade (glutactins)	2	4	1	1	0	1
	I clade (unknown function)	1	1	1	1	1	0
	J clade (acetylcholinesterases)	1	1	2	2	2	2
	K clade (gliotactins)	2	2	1	1	1	2
	L clade (neuroligins)	6	4	5	5	3	6
	M clade (neurotactins)	2	2	2	1	0	2
	**Not determined**	5	-	-	-	-	-
	Total	22	35	49	24	30	76
**GSTs**							
	Delta	0	11	3	1	16	5
	Epsilon	0	14	19	0	1	8
	Omega	1	4	3	1	2	4
	Sigma	8	1	7	4	6	2
	Theta	2	4	1	1	2	1
	Zeta	1	2	1	1	0	2
	Others	0	1	2	0	3	1
	Microsomal	3	3	5	2	2	0
	Kappa (Mitochondrial)	0	0	0	0	0	0
	Not determined	3	-	-	-	-	-
	Total	18	40	41	10	32	23

Abbreviations are as follows: CS - *Calopteryx splendens*; DM - *Drosophila melanogaster*; TC - *Tribolium castaneum*; AM - *Apis mellifera*; AP - *Acyrthosiphon pisum*; BM - *Bombyx mori*. Data are taken from Yu et al. 2008, Yu et al. 2009, Claudianos et al. 2006, Tribolium Genome Sequencing Consortium 2008, Shi et al. 2012, Ramsey et al. 2010, Roncalli et al. 2015 and Baldwin et al. 2009. CCE – carboxyl/cholinesterases; CYP – cytochrome P450 monooxygenases; GST – glutathione S-transferases.

Table S2: Accession numbers of the reference opsins

**BLAST searches**	AFM75824.1, AAA02499.1, AAA69069.1, BAG14332.1, AAC26329.1, CAC86665.1, EFX83617.1, NP_150598.1, AAV63834.1, AAA30674.1, NP_001138950.1, EFX86931.1, BAC76021.1, BAJ22674.1, BAC76019.1, FAA00384.1
**HMMer searches, Cnidopsins**	BAD67141.1, BAD67142.1, BAD67146.1, BAF95825.1, BAF95829.1, BAF95844.1, BAG80696.1
**HMMer searches, Vertebrate c-opsins**	NP_064445.1, NP_571267.1, NP_571329.1, NP_571250.1, NP_571394.1, AAM77793.1, NP_878311.1, NP_878312.1, NP_571328.2, BAD17961.1, NP_001002443.1, AAY56361.1, AAZ79904.1, ABB88727.1, AAI71332.1, ADI59671.1, ADI59673.1, ADI59675.1
**HMMer searches, Arthropod pteropsins**	XP_312502.2, NP_001035057.1, EFX86931.1, ADZ24786.1, XP_312503.4
**HMMer searches, Group 4 opsins**	NP_006574.1, NP_033128.1, NP_002912.2, BAC76019.1, BAC76020.1, BAC76023.1, AAR02098.1, AAR02099.1, NP_859528.1, BAJ22674.1
**HMMer searches, Arthropsins**	EFX83617.1, EFX83618.1, EFX83619.1, EFX83830.1, EFX83831.1, EFX84031.1, EFX84032.1, EFX84250.1
**HMMer searches, Melanopsins**	AAK59988.1, NP_038915.1, NP_150598.1, AAL82577.1, AAM95160.1, AAO20043.1, AAX73255.1, AAX73256.1, BAE00065.1
**HMMer searches, Non-arthropod r-opsins**	CAA30644.1, CAA49906.1, CAA40108.1, CAA88923.1, AAC26329.1, BAA22217.1, AAD28720.1, CAB89516.1, AAF73286.1, CAC86665.1, AAR18073.1, CAD13146.1, ACB05673.1, CAX73070.1
**HMMer searches, Arthropod visual opsins**	AAA02498.1, AAA02499.1, AAA69069.1, AAC05091.1, AAC05092.1, AAG17119.1, AAG17120.1, AAL59876.1, AAL59877.1, AAL59878.1, AAS55401.1, AAT73202.1, AAU07978.1, AAW80336.1, AAL59879.2, BAG14330.1, BAG14331.1, BAG14332.1, BAG14333.1, BAG14334.1, BAG14335.1, ACH56536.1, ACO05013.1, BAH56227.1, EFX75461.1, EFX81332.1
**HMMer searches, Onychopsins**	AFM43712.1, AFM43711.1, AFM43710.1, AFM75824.1, AFM75825.1

Table S3: BUSCO scores based on a highly conserved arthropod BUSCO set.

	**CSPLE[1]**	**ZNEVA**	**APISU**	**ISCAP**	**AALBO**	**AAEGY**	**LMIGR**
BUSCO[2] (genome)	95.3 (0.2), 2.7, 2.0	97.7 (0.6), 1.0, 1.3	96.8 (4.0), 0.7, 2.5	83.9 (0.4), 7.9, 8.2	95.4 (22.5), 1.7, 2.9	97.3 (5.7), 0.7, 2.0	82.9 (2.6), 10.6, 6.5
BUSCO[2] (gene set)	95.0 (0.6), 4.4, 0.6	98.2 (1.1), 0.9, 0.9	98.8 (5.4), 0.2, 1.0	86.4 (1.0), 9.9, 3.7	89.6 (18.7), 2.9, 7.5	97.0 (6.0), 1.1, 1.9	87.9 (3.6), 7.2, 4.9

[1] Abbreviations used for species names; CSPLE - *Calopteryx splendens*; ZNEVA - *Zootermopsis nevadensis*; APISU - *Acyrthosiphon pisum*; ISCAP - *Ixodes scapularis*; AALBO - *Aedes albopictus*; AAEGY - *Aedes aegypti*; LMIGR – *Locusta migratoria*.

[2] BUSCO completeness scores are in the format: % complete BUSCOs (of which, duplicated), % fragmented BUSCOs, % missing BUSCOs.

Table S4: Repetitive elements present in the genome of *Calopteryx splendens.*

Family	Number of family members	Average size of family members	% of assembly
Unknown	403937	210	5.206
LINE/RTE-BovB	192600	396	4.683
LINE/CR1	89618	377	2.077
Simple_repeat	593497	40	1.473
SINE/tRNA	95332	217	1.272
DNA/TcMar-Mariner	29486	397	0.719
Low_complexity	149468	50	0.463
LINE/L2	27636	240	0.407
LINE/Penelope	18941	307	0.357
DNA/PiggyBac	9299	393	0.225
SINE/tRNA-Deu-L2	14085	186	0.161
DNA/TcMar-Tc2	7766	288	0.138
DNA/TcMar-Tigger	6719	310	0.128
LTR/Gypsy	15162	134	0.125
DNA/hAT-Charlie	6931	286	0.122
DNA	7289	165	0.074
LINE/Jockey	3824	276	0.065
DNA/Zator	2754	365	0.062
LINE/L1	6262	151	0.058
DNA/hAT-Tip100	4040	228	0.057
LINE/Dong-R4	1962	464	0.056
SINE/MIR	5317	167	0.055
LINE/I	3789	201	0.047
LTR/Copia	5213	136	0.043
RC/Helitron	4197	151	0.039
SINE?	4558	133	0.037
DNA/hAT-Ac	2101	288	0.037
DNA/CMC-EnSpm	5332	107	0.035
DNA/TcMar-Pogo	1664	324	0.033
SINE/tRNA-Deu-CR1	2893	152	0.027
DNA/CMC-Transib	1751	222	0.024
LINE/I-Nimb	1063	353	0.023
DNA/hAT-hATm	1762	204	0.022
LTR/Pao	2431	140	0.021
LINE	1053	257	0.017
DNA/Maverick	1782	128	0.014
LTR/ERV1	2430	92	0.014
DNA/CMC-Chapaev-3	742	298	0.014
DNA/hAT	664	290	0.012
DNA/TcMar-Fot1	1025	186	0.012
DNA/MULE-MuDR	1607	104	0.010
tRNA	2319	70	0.010
DNA/TcMar-Tc1	1008	152	0.009
LINE/LOA	701	217	0.009
RNA	956	153	0.009
DNA/Sola	771	172	0.008
DNA/hAT-Tol2	152	848	0.008
DNA/PIF-Harbinger	1139	110	0.008
LINE/CR1-Zenon	926	118	0.007
DNA/hAT-Blackjack	911	112	0.006
DNA/Crypton	460	215	0.006
Other	14500	75	0.078

Table S5: Top ten InterPro domains present in the gene set of *Calopteryx splendens.*

InterPro accession[1]	Description	Number of genes
IPR001680	WD40 repeat	131
IPR020683	Ankyrin repeat-containing domain	104
IPR000504	RNA recognition motif domain	92
IPR000719	Protein kinase domain	88
IPR000618	Insect cuticle protein (Family)	88
IPR013098	Immunoglobulin I-set (Domain)	88
IPR007087	Zinc finger, C2H2 (Domain)	81
IPR001611	Leucine-rich repeat	73
IPR001254	Serine proteases, trypsin domain	64
IPR001356	Homeobox domain	56

[1] The entries shown have >75% of the corresponding Pfam HMM profile is covered. Also, entries related to transposable elements were omitted.

Table S6: *Calopteryx splendens* genes with similarity to those of Bacteria.

Gene name	Likely bacterial source	Function	Near either end of a scaffold?	Coverage dips?	Transcribed?	Full-length?	Introns
CSPLE_10102	Wolbachia (Nasonia vitripennis)	ABC transporter ATP-binding protein	Yes	No	No	Yes	0
CSPLE_12238	Erwinia tracheiphila	hypothetical protein	No	No	No	Yes	8
CSPLE_12763	Frankia symbiont of Datisca glomerata	hypothetical protein	Yes	No	No	Yes	9
CSPLE_02463	Wolbachia	peptide chain release factor 2	No	No	No	Yes	1
CSPLE_17379	Staphylococcus epidermidis	hypothetical protein	Yes	No	No	Yes	2
CSPLE_21547	Persephonella sp. IF05-L8	hypothetical protein	Yes	No	No	Yes	0
CSPLE_08439	Rhodococcus sp. P14	transcription termination factor Rho	Yes	No	No	Yes	9
CSPLE_09629	Bacillus safensis FO-36b	collagen-like protein	No	No	No	Yes	2
CSPLE_09810	[Clostridium] sordellii	Ribonucleases G and E	No	No	No	Yes	2
CSPLE_10989	Candidatus Entotheonella sp. TSY2	hypothetical protein ETSY2_47035	No	No	No	Yes	2
CSPLE_11223	Wolbachia (Drosophila ananassae)	hypothetical protein	No	No	No	No	3
CSPLE_12099	Wolbachia (Cimex lectularius)	aspartate-semialdehyde dehydrogenase	Yes	No	No	Yes	3
CSPLE_12401	Wolbachia (Onchocerca volvulus)	ABC transporter ATP-binding protein	Yes	No	No	Yes	2
CSPLE_12607	Wolbachia (Drosophila simulans)	Thiol-disulfide interchange protein DsbA	No	No	No	Yes	2
CSPLE_13435	Salmonella enterica Typhi	Retron-type reverse transcriptase	No	No	Yes	No	2
CSPLE_15733	Candidatus Entotheonella sp. TSY2	hypothetical protein ETSY2_47035	No	No	No	No	3
CSPLE_17036	Escherichia coli	hypothetical protein	No	No	No	No	3
CSPLE_17069	Escherichia coli	hypothetical protein	No	No	No	Yes	1
CSPLE_04108	Wolbachia (Drosophila melanogaster)	Transposase	No	No	No	Yes	1
CSPLE_04109	Wolbachia	Transposase	No	No	No	Yes	1
CSPLE_17600	Salmonella enterica Typhi	Retron-type reverse transcriptase	No	No	Yes	Yes	6
CSPLE_17847	Escherichia coli	hypothetical protein	Yes	Yes	No	Yes	2
CSPLE_18224	Beggiatoa sp. PS	transposase	Yes	No	No	Yes	2
CSPLE_18786	Solemya velum gill symbiont	hypothetical protein	No	Yes	No	No	2
CSPLE_05115	Escherichia coli	hypothetical protein	No	No	Yes	Yes	1
CSPLE_19937	Solemya velum gill symbiont	hypothetical protein	No	No	No	Yes	1
CSPLE_20234	Wolbachia (Culex pipiens molestus)	hypothetical protein, partial	Yes	No	Yes	Yes	1
CSPLE_20708	Ardenticatena maritima	AMP-dependent synthetase	Yes	No	No	No	6
CSPLE_20973	Wolbachia (Culex quinquefasciatus JHB)	Ankyrin repeat domain protein	No	No	Yes	Yes	2
CSPLE_21033	Solemya velum gill symbiont	hypothetical protein	No	No	No	Yes	1
CSPLE_21365	uncultured gamma proteobacterium	ankyrin 2,3/unc44	No	No	Yes	Yes	7
CSPLE_21691	Streptococcus suis	hypothetical protein	Yes	No	No	No	1
CSPLE_21862	Wolbachia pipientis	hypothetical protein, partial	No	No	Yes	No	3
CSPLE_08120	Wolbachia pipientis	Transposase	No	No	No	Yes	3
CSPLE_08297	Lactobacillus iners LEAF 2053A-b	F5/8 type C domain protein	No	No	Yes	No	7
CSPLE_08801	Escherichia coli	hypothetical protein	No	No	Yes	No	1
CSPLE_09526	Wolbachia	ATPase	No	No	No	Yes	1
CSPLE_09648	Borrelia finlandensis	hypothetical protein BSV1_D13	Yes	No	No	Yes	0
CSPLE_09669	Chlamydia trachomatis	hypothetical protein	No	No	No	Yes	1
CSPLE_02007	Wolbachia (Drosophila ananassae)	Pol protein	No	No	No	Yes	5
CSPLE_14326	Wolbachia (Drosophila simulans)	phosphoribosylglycinamide formyltransferase	No	No	No	Yes	1
CSPLE_15900	Wolbachia (Culex quinquefasciatus JHB)	Transposase	No	No	No	Yes	1
CSPLE_17390	Wolbachia (Nasonia vitripennis)	membrane protein	Yes	No	No	Yes	1
CSPLE_18660	Aeromonas schubertii	AMP-binding protein	No	No	Yes	Yes	3
CSPLE_18828	Ehrlichia ruminantium	hypothetical protein	No	No	No	Yes	1
CSPLE_20038	Wolbachia pipientis	hypothetical protein, partial	Yes	No	No	Yes	0
CSPLE_05977	Wolbachia	Phage baseplate assembly protein V	No	No	No	Yes	2
CSPLE_07601	Paenibacillus terrae	peptidase	No	No	No	Yes	1
CSPLE_22361	Aster yellows phytoplasma	DNA polymerase	No	No	No	Yes	1
CSPLE_08681	Wolbachia (Drosophila simulans)	DNA mismatch repair protein MutL-1	No	No	No	Yes	1

Table S7: Counts of immune-related gene family members in the genome of *Calopteryx splendens* and eight other arthropods, identified by the presence of characteristic InterPro domains.

Immune Phase	Gene Family	Characteristic InterPro Domain(s)	Counts of genes with InterPro domain matches per species (counts with > 75% of PFAM profile matched, if exists)								
			Calopteryx splendens	*Zootermopsis nevadensis*	*Pediculus humanus*	*Acyrthosiphon pisum*	*Apis mellifera*	*Tribolium castaneum*	*Danaus plexippus*	*Drosophila melanogaster*	*Daphnia pulex*
Recognition	GNBPs	IPR000757	1 (0)	6 (3)	0	2 (0)	2 (0)	3 (2)	5 (2)	3 (0)	10 (4)
	PGRPs	IPR006619*	12 (10)	6	1	0	4	7	7	13	0
	FREPs	IPR002181	3	4 (3)	2	2	2	8 (7)	3 (2)	14 (13)	40 (4)
	GALEs	IPR001079	4 (1)	4	3	1	3	3	4	6	5
	MLs	IPR003172	9 (2)	6 (4)	3	19 (13)	4	9	6	9 (8)	10
	SCRAs	IPR001190	4 (3)	7 (3)	7 (5)	6 (4)	5 (3)	5 (3)	4 (2)	5 (4)	8 (4)
	SCRBs	IPR002159	23 (4)	11 (10)	15 (11)	9 (8)	10 (8)	18 (16)	20 (9)	14	9 (7)
	SCRCs	IPR000436	17 (13)	14 (12)	11 (9)	18 (7)	12 (9)	15 13)	12 (9)	15 (11)	24 (19)
	TEPs	IPR001599	3	4	3	4	4	4	4	6	6
		IPR009048	3	5 (4)	3	3	3	4	4	6	8 (7)
		IPR011626	4 (2)	4	3 (2)	3	4 (3)	4 (3)	4	6 (5)	9 (6)
		**SUM**	**77**	**63**	**45**	**61**	**46**	**57**	**65**	**85**	**115**
Modulation	CTLs	IPR001304	25 (17)	30 (24)	14 (11)	13 (11)	13 (12)	16 (11)	19 (15)	42 (36)	54 (40)
	SRPNs	IPR023796	12 (5)	14 (12)	15 (9)	21 (14)	7 (5)	25 (24)	30 (21)	30 (28)	6
	IAPs	IPR001370	9 (5)	7 (6)	3	85 (74)	5	4	4 (2)	4	7 (5)
	CASPs	IPR011600	16 (6)	7	5 (4)	6	6 (4)	7	4	7	18 (9)
		**SUM**	**62**	**58**	**37**	**125**	**31**	**52**	**57**	**83**	**85**
Signalling	RELs	IPR011539	6 (1)	2	4 (2)	2	4	4	3	4	4 (2)
	TOLLs	IPR000157	9 (6)	11 (6)	8 (4)	10 (7)	9 (5)	12 (4)	15 (6)	11 (3)	10 (4)
	SPZs	IPR032104	5 (4)	7 (5)	7 (6)	9	6	12 (11)	8	5	48 (47)
		**SUM**	**20**	**20**	**19**	**21**	**19**	**28**	**26**	**20**	**62**
Effectors	DEFs	IPR001542	1	0	2	0	2	4	0	1	0
	LYSs	IPR023346*	3 (1)	3	1	4 (3)	4 (3)	2	5	15 (13)	4
	CATs	IPR011614	1	3	1	3	3	3	5	2	1
	PPOs	IPR005204	6 (1)	5 (4)	3 (2)	2 (1)	5	9	9 (8)	10	1 (0)
		IPR005203	6 (3)	5 (4)	3	2	5	9 (8)	9	10 (9)	1
	SODs	IPR001424	7 (4)	4	4	4	3	5	6 (5)	5	16 (13)
		IPR001189*	1 (0)	1	1	1	2	1	1	1	1
	HPXs	IPR010255*	31 (12)	11 (10)	11	83 (51)	11 (9)	11	12 (10)	10	62 (49)
	GPXs	IPR000889	3 (2)	2 (1)	3 (1)	4	2	3	3 (2)	2	8 (2)
	TPXs	IPR019479	4 (3)	6	4 (3)	6 (3)	5 (4)	6	4	8 (7)	4 (3)
		**SUM**	**63**	**40**	**33**	**109**	**42**	**53**	**54**	**64**	**98**

* No PFAM profile, used SUPERFAM (LYSs & HPXs), SMART (PGRPs), and PANTHER (SODs).

Table S8: Results of searching UniParc for multi-domain PGRPs.

UniParc ID	Organism	Number of PGRP domains	RefSeq ID (or other ID)	Status	Comments
UPI000533D486	*Saimiri boliviensis* Bolivian squirrel monkey	4	XP_010327963	active	Almost certainly an annotation artefact, i.e. the fusion of two neighbouring genes (2+2 domains) *PGLYRP3* and *PGLYRP4* found in other mammals.
UPI0006961A68	*Lingula unguis* tailed mussel	4	XP_013388318	active	May be the fusion of a one domain gene and a three domains gene, evidence for UTR in within the transcript. http://www.ncbi.nlm.nih.gov/gene?cmd=retrieve&list_uids=106157269
UPI000696F210		4	XP_013388619	active	May be the fusion of a one domain gene and a three domains gene, evidence for UTR in within the transcript. http://www.ncbi.nlm.nih.gov/gene?cmd=retrieve&list_uids=106157492
UPI000698A105		4	XP_013388626	active	Isoform of UPI000696F210
UPI0000079DCF	*Drosophila melanogaster* fruit fly	3	FlyBase CG4432-PA	inactive	Incorrect annotation of the *PGRP-LC* gene with its 3 PGRP domains fused into a single transcript. The annotation was subsequently corrected such that each of the three alternatively spliced domains contains a single PGRP domain.
UPI0000124831		3	Ensembl CG4432-PA	inactive	As for UPI0000079DCF
UPI00001E19BE		3	TROME NT_037436_1082_0	inactive	As for UPI0000079DCF
UPI000177C7CB	*Drosophila ananassae* fruit fly	3	EnsemblMetazoa FBpp0113872	active	As for UPI0000079DCF
UPI00017D8211	*Drosophila willistoni* fruit fly	3	XP_002069134	active	This appears to be a legitimate 3-domain PGRP gene where the normally 2-domain *PGRP-LF* gene has acquired a third domain that duplicated of the nearby third domain of *PGRP-LC*.
UPI0004E482B9	*Rattus norvegicus* rat	3	XP_008759365	inactive	Removed from RefSeq. Corresponds to *PGLYRP4*, whose current UniProt entry has only two domains.
UPI00059686F1	*Bactrocera cucurbitae* melon fruit fly	3	XP_011183865	active	Appears to be an incorrect annotation of the *PGRP-LC* gene with its 3 PGRP domains fused into a single transcript.
UPI0006A19F1E	*Lucilia cuprina* sheep blowfly	3	UniProtKB/TrEMBL A0A0L0C1T7	active	Appears to be a gene with 3 *PGRP-LE*-like domains, however, the last exons with the last domain are separated from the rest by an assembly gap of about 500bp, so it remains unclear.

C) References

Ai M et al. 2010. Acid sensing by the *Drosophila* olfactory system. Nature. 468:691-695.

Bairoch A, Boeckmann B, Ferro S and Gasteiger E. 2004. Swiss-Prot: juggling between evolution and stability. Brief Bioinform. 5:39-55.

Baldwin WS, Marko PB and Nelson DR. 2009. The cytochrome P450 (CYP) gene superfamily in *Daphnia pulex*. BMC Genomics. 10:169-169.

Barribeau SM et al. 2015. A depauperate immune repertoire precedes evolution of sociality in bees. Genome Biol. 16:83-83.

Bartholomay LC et al. 2010. Pathogenomics of *Culex quinquefasciatus* and meta-analysis of infection responses to diverse pathogens. Science. 330:88-90.

Benoit JB et al. 2016. Unique features of a global human ectoparasite identified through sequencing of the bed bug genome. Nat Commun. 7:10165-10165.

Benton R. 2015. Multigene Family Evolution: Perspectives from Insect Chemoreceptors. Trends Ecol Evol. 30:590-600.

Birney E, Clamp M and Durbin R. 2004. GeneWise and Genomewise. Genome Res. 14:988-995.

Boetzer M, Henkel CV, Jansen HJ, Butler D and Pirovano W. 2011. Scaffolding pre-assembled contigs using SSPACE. Bioinformatics. 27:578-579.

Camacho C et al. 2009. BLAST+: architecture and applications. BMC Bioinf. 10:421-421.

Campbell MS, Holt C, Moore B and Yandell M. 2014. Genome Annotation and Curation Using MAKER and MAKER-P. Curr Protoc Bioinformatics. 48:4.11.1-4.1139.

Capella-Gutierrez S, Silla-Martinez JM and Gabaldon T. 2009. trimAl: a tool for automated alignment trimming in large-scale phylogenetic analyses. Bioinformatics. 25:1972-1973.

Chauhan P et al. 2014. De novo transcriptome of *Ischnura elegans* provides insights into sensory biology, colour and vision genes. BMC Genomics. 15:808-808.

Chipman AD et al. 2014. The first myriapod genome sequence reveals conservative arthropod gene content and genome organisation in the centipede *Strigamia maritima*. PLoS Biol. 12:e1002005.

Claudianos C et al. 2006. A deficit of detoxification enzymes: pesticide sensitivity and environmental response in the honeybee. Insect Mol Biol. 15:615-636.

Corbet PS. 1980. Biology of Odonata. Ann Rev Entomol. 25:189-217.

Cordoba-Aguilar A and Cordero-Rivera A. 2005. Evolution and ecology of Calopterygidae (Zygoptera:Odonata): status of knowledge and research perspectives. Neotrop Entomol. 34:861-879.

Corey EA, Bobkov Y, Ukhanov K and Ache BW. 2013. Ionotropic crustacean olfactory receptors. PLoS One. 8:e60551.

Crespo JG. 2011. A review of chemosensation and related behavior in aquatic insects. J Insect Sci. 11:62.

Croset V et al. 2010. Ancient protostome origin of chemosensory ionotropic glutamate receptors and the evolution of insect taste and olfaction. PLos Genet. 6:e1001064-e1001064.

Engsontia P et al. 2008. The red flour beetle's large nose: an expanded odorant receptor gene family in *Tribolium castaneum*. Insect Biochem Mol Biol. 38:387-397.

Enjin A et al. 2016. Humidity Sensing in *Drosophila*. Curr Biol. 26:1352-1358.

Frati F, Piersanti S, Conti E, Rebora M and Salerno G. 2015. Scent of a Dragonfly: Sex Recognition in a Polymorphic Coenagrionid. PLoS One. 10:e0136697.
Frati F, Piersanti S, Rebora M and Salerno G. 2016. Volatile cues can drive the oviposition behavior in Odonata. J Insect Physiol. 91-92:34-38.
Freeman EG, Wisotsky Z and Dahanukar A. 2014. Detection of sweet tastants by a conserved group of insect gustatory receptors. Proc Natl Acad Sci U S A. 111:1598-1603.
Fujii S et al. 2015. *Drosophila* sugar receptors in sweet taste perception, olfaction, and internal nutrient sensing. Curr Biol. 25:621-627.
Futahashi R et al. 2015. Extraordinary diversity of visual opsin genes in dragonflies. Proc Natl Acad Sci USA. 112:E1247-E1256.
Groh-Lunow KC, Getahun MN, Grosse-Wilde E and Hansson BS. 2015. Expression of ionotropic receptors in terrestrial hermit crab's olfactory sensory neurons. Front Cell Neurosci. 8:448.
Gulia-Nuss M et al. 2016. Genomic insights into the *Ixodes scapularis* tick vector of Lyme disease. Nat Commun. 7:10507-10507.
Haas BJ et al. 2013. De novo transcript sequence reconstruction from RNA-seq using the Trinity platform for reference generation and analysis. Nat Protoc. 8:1494-1512.
Han J et al. 2015. Identification of the full 46 cytochrome P450 (CYP) complement and modulation of CYP expression in response to water-accommodated fractions of crude oil in the cyclopoid copepod *Paracyclopina nana*. Environ Sci Technol Lett. 49:6982-6992.
He Z et al. 2016. Evolview v2: an online visualization and management tool for customized and annotated phylogenetic trees. Nucleic Acids Res. 44:W236-241.
Hering L and Mayer G. 2014. Analysis of the opsin repertoire in the tardigrade *Hypsibius dujardini* provides insights into the evolution of opsin genes in panarthropoda. Genome Biol Evol. 6:2380-2391.
Hering L et al. 2012. Opsins in onychophora (velvet worms) suggest a single origin and subsequent diversification of visual pigments in arthropods. Mol Biol Evol. 29:3451-3458.
Hoy MA et al. 2016. Genome Sequencing of the Phytoseiid Predatory Mite *Metaseiulus occidentalis* Reveals Completely Atomized Hox Genes and Superdynamic Intron Evolution. Genome Biol Evol. 8:1762-1775.
Johnston PR and Rolff J. 2013. Immune- and wound-dependent differential gene expression in an ancient insect. Dev Comp Immunol. 40:320-324.
Jones P et al. 2014. InterProScan 5: genome-scale protein function classification. Bioinformatics. 30:1236-1240.
Joseph RM and Carlson JR. 2015. *Drosophila* Chemoreceptors: A Molecular Interface Between the Chemical World and the Brain. Trends Genet. 31:683-695.
Jung JW, Park KW, Ahn YJ and Kwon HW. 2015. Functional characterization of sugar receptors in the western honeybee, *Apis mellifera*. Journal of Asia-Pacific Entomology. 18:19-26.
Katoh K and Standley DM. 2013. MAFFT multiple sequence alignment software version 7: improvements in performance and usability. Mol Biol Evol. 30:772-780.
Kent LB and Robertson HM. 2009. Evolution of the sugar receptors in insects. BMC Evol Biol. 9:41.

Knecht ZA et al. 2016. Distinct combinations of variant ionotropic glutamate receptors mediate thermosensation and hygrosensation in *Drosophila*. Elife. 5:e17879.
Koh TW et al. 2014. The *Drosophila* IR20a clade of ionotropic receptors are candidate taste and pheromone receptors. Neuron. 83:850-865.
Kriventseva EV et al. 2015. OrthoDB v8: update of the hierarchical catalog of orthologs and the underlying free software. Nucleic Acids Res. 43:D250-D256.
Krogh A, Larsson B, von Heijne G and Sonnhammer EL. 2001. Predicting transmembrane protein topology with a hidden Markov model: application to complete genomes. J Mol Biol. 305:567-580.
Kulmuni J and Havukainen H. 2013. Insights into the evolution of the CSP gene family through the integration of evolutionary analysis and comparative protein modeling. PLoS One. 8:e63688-e63688.
Larkin MA et al. 2007. Clustal W and Clustal X version 2.0. Bioinformatics. 23:2947-2948.
Lee E et al. 2013. Web Apollo: a web-based genomic annotation editing platform. Genome Biol. 14:R93-R93.
Li J, Waterhouse RM and Zdobnov EM. 2011. A remarkably stable *TipE* gene cluster: evolution of insect Para sodium channel auxiliary subunits. BMC Evol Biol. 11:337.
Missbach C et al. 2014. Evolution of insect olfactory receptors. Elife. 3:e02115-e02115.
Miyamoto T and Amrein H. 2014. Diverse roles for the *Drosophila* fructose sensor Gr43a. Fly. 8:19-25.
Ni L et al. 2016. The Ionotropic Receptors IR21a and IR25a mediate cool sensing in *Drosophila*. Elife. 5. pii:e13254.
Palczewski K et al. 2000. Crystal structure of rhodopsin: A G protein-coupled receptor. Science. 289:739-745.
Palmer WJ and Jiggins FM. 2015. Comparative genomics reveals the origins and diversity of arthropod immune systems. Mol Biol Evol. 32:2111-2129.
Piersanti S, Frati F, Conti E, Rebora M and Salerno G. 2014a. The sense of smell in Odonata: an electrophysiological screening. J Insect Physiol. 70:49-58.
Piersanti S et al. 2014b. First evidence of the use of olfaction in Odonata behaviour. J Insect Physiol. 62:26-31.
Piersanti S, Frati F, Rebora M and Salerno G. 2016. Carbon dioxide detection in adult Odonata. Zoology (Jena). 119:137-142.
Penalva-Arana DC, Lynch M and Robertson HM. 2009. The chemoreceptor genes of the waterflea *Daphnia pulex*: many Grs but no Ors. BMC Evol Biol. 9:79-79.
Petersen TN, Brunak S, von Heijne G and Nielsen H. 2011. SignalP 4.0: discriminating signal peptides from transmembrane regions. Nat Methods. 8:785-786.
Prieto-Godino LL et al. 2016. Olfactory receptor pseudo-pseudogenes. Nature. 539:93-97.
Ramsey JS et al. 2010. Comparative analysis of detoxification enzymes in *Acyrthosiphon pisum* and *Myzus persicae*. Insect Mol Biol. 19 Suppl 2:155-164.
Rebora M, Piersanti S, Dell'Otto A and Gaino E. 2013. The gustatory sensilla on the endophytic ovipositor of Odonata. Arthropod Struct Dev. 42:127-134.
Rebora M, Gaino E and Piersanti S. 2014. The epipharyngeal sensilla of the damselfly *Ischnura elegans* (Odonata, Coenagrionidae). Micron. 66:31-36.
Rebora M, Salerno G, Piersanti S, Dell'otto A and Gaino E. 2012. Olfaction in dragonflies: electrophysiological evidence. J Insect Physiol. 58:270-277.
Reese MG, Eeckman FH, Kulp D and Haussler D. 1997. Improved splice site detection in Genie. J Comp Biol. 4:311-323.

Robertson HM and Wanner KW. 2006. The chemoreceptor superfamily in the honey bee, *Apis mellifera*: expansion of the odorant, but not gustatory, receptor family. Genome Res. 16:1395-1403.
Robertson HM, Gadau J and Wanner KW. 2010. The insect chemoreceptor superfamily of the parasitoid jewel wasp *Nasonia vitripennis*. Insect Mol Biol. 19 Suppl 1:121-136.
Robertson HM and Kent LB. 2009. Evolution of the gene lineage encoding the carbon dioxide receptor in insects. J Insect Sci. 9:19.
Robertson HM, Warr CG and Carlson JR. 2003. Molecular evolution of the insect chemoreceptor gene superfamily in *Drosophila melanogaster*. Proc Natl Acad Sci USA. 100 Suppl 2:14537-14542.
Robertson HM. 2015. The Insect Chemoreceptor Superfamily Is Ancient in Animals. Chem Senses. 40:609-614.
Robinson KM, Sieber KB and Dunning Hotopp JC. 2013. A review of bacteria-animal lateral gene transfer may inform our understanding of diseases like cancer. PLos Genet. 9:e1003877-e1003877.
Roncalli V, Cieslak MC, Passamaneck Y, Christie AE and Lenz PH. 2015. Glutathione S-transferase (GST) gene diversity in the crustacean *Calanus finmarchicus*--contributors to cellular detoxification. PLoS One. 10:e0123322-e0123322.
Rytz R, Croset V and Benton R. 2013. Ionotropic receptors (IRs): chemosensory ionotropic glutamate receptors in *Drosophila* and beyond. Insect Biochem Mol Biol. 43:888-897.
Saina M et al. 2015. A cnidarian homologue of an insect gustatory receptor functions in developmental body patterning. Nat Commun. 6:6243.
Shi H et al. 2012. Glutathione S-transferase (GST) genes in the red flour beetle, *Tribolium castaneum*, and comparative analysis with five additional insects. Genomics. 100:327-335.
Silbering AF et al. 2011. Complementary function and integrated wiring of the evolutionarily distinct *Drosophila* olfactory subsystems. J Neurosci. 31:13357-13375.
Simao FA, Waterhouse RM, Ioannidis P, Kriventseva EV and Zdobnov EM. 2015. BUSCO: assessing genome assembly and annotation completeness with single-copy orthologs. Bioinformatics. 31:3210-3212.
Smadja C, Shi P, Butlin RK and Robertson HM. 2009. Large gene family expansions and adaptive evolution for odorant and gustatory receptors in the pea aphid, *Acyrthosiphon pisum*. Mol Biol Evol. 26:2073-2086.
Smith CR et al. 2011. Draft genome of the red harvester ant *Pogonomyrmex barbatus*. Proc Natl Acad Sci USA. 108:5667-5672.
Stamatakis A. 2006. RAxML-VI-HPC: maximum likelihood-based phylogenetic analyses with thousands of taxa and mixed models. Bioinformatics. 22:2688-2690.
Stewart S, Koh TW, Ghosh AC and Carlson JR. 2015. Candidate ionotropic taste receptors in the *Drosophila* larva. Proc Natl Acad Sci U S A. 112:4195-4201.
Stoks R and Cordoba-Aguilar A. 2012. Evolutionary ecology of Odonata: a complex life cycle perspective. Annu Rev Entomol. 57:249-265.
Suzek BE et al. 2015. UniRef clusters: a comprehensive and scalable alternative for improving sequence similarity searches. Bioinformatics. 31:926-932.
Terrapon N et al. 2014. Molecular traces of alternative social organization in a termite genome. Nat Commun. 5:3636-3636.
Towb P, Sun H and Wasserman SA. 2009. Tube Is an IRAK-4 homolog in a Toll pathway adapted for development and immunity. J Innate Immun. 1:309-321.

Tribolium Genome Sequencing Consortium. 2008. The genome of the model beetle and pest *Tribolium castaneum*. Nature. 452:949-955.
Uniprot Consortium. 2015. UniProt: a hub for protein information. Nucleic Acids Res. 43:D204-D212.
Wanner KW and Robertson HM. 2008. The gustatory receptor family in the silkworm moth *Bombyx mori* is characterized by a large expansion of a single lineage of putative bitter receptors. Insect Mol Biol. 17:621-629.
Wanner KW et al. 2007. Female-biased expression of odourant receptor genes in the adult antennae of the silkworm, *Bombyx mori*. Insect Mol Biol. 16:107-119.
Waterhouse RM et al. 2007. Evolutionary dynamics of immune-related genes and pathways in disease-vector mosquitoes. Science. 316:1738-1743.
Ye C, Ma ZS, Cannon CH, Pop M and Yu DW. 2012. Exploiting sparseness in de novo genome assembly. BMC Bioinf. 13 Suppl 6:S1-S1.
Yu Q et al. 2008. Identification, genomic organization and expression pattern of glutathione S-transferase in the silkworm, *Bombyx mori*. Insect Biochem Mol Biol. 38:1158-1164.
Yu Q-Y, Lu C, Li W-L, Xiang Z-H and Zhang Z. 2009. Annotation and expression of carboxylesterases in the silkworm, *Bombyx mori*. BMC Genomics. 10:553-553.

D) Curated protein sequences

Gustatory Receptors (GRs)

>CsplGr1
MLLKRVFRMPNFVVRHSPMKISPEPLSLITSTKREKIIDKTHYFEPDQVNTSADEDGSVEWTDTALLPTLKLASCF
GMLPVSVLKSKRQHGKQSITFTFSWSSWRSLATLFLLFCFASIEATALKYMFNGFYHSTKLEHVEHTTAGAAFYG
YSFISTLLHLRLATRWSYLLHSWHNAEAPLLLNKGESIVGKIYKARKWGKLRGNQKTRLWHKIFPQTLKSRFARLT
AFILTIALSTVLRRFHRVMSYFDHVEDKCLDTIFPPNANFALQILENFSRRSHWFVYEETAYATWKGLAIIGISIVATFI
WNFTDVLIMILSMALATRFKLLNTKLSLLQGKAVKPSHWEGLRCQYTSLSQLSRTLDDHINGITFFSIASNLYFTCIQ
LLSGLQTDNNRTLEKTAFYFYSFSFMVGRATGVILLASSINQETLNISKHLYGCPHHSYCKEVQRFMTELTTDFVAL
SGLNLFYITRNFLLGVAGAVVTYEFVLLQLDTGSE
>CsplGr2
MLANWIKKTRIDPFQENTPFASSWKDGNGLFPEGKQKMDDDVQKKRSKRRRRSNVRHARIGTFFNTLEYILRAL
RMASCLPLTVEKEFDTLEFRIRSLSFAFSVITYSAWSVIVWIVVQERLDIIVQSSHSFDDSVIAYVFIIYFGEWPMLPF
IRWFWQTPKLLTFLSKWRNFEDQFQLITGAPLELDQEMSRSLVAAVATTVPLLTLLGAVASHYLMPMMPSMHLAA
YWIIATQMYLNSAFVWATSKALQAASKGLVQGLRRDATASMRADAGSVFIGPGTRRWPQEYMELWRKLSIMVRY
SGNGAIGTAFILNIGKEFPAMLLAAYGVIAWVDGGTQGNESGVLLILIHHLADLILLGNSAHNAAHSAGSDIRDELSL
MMVKPRTSHKIREFLQLMDASPPYITVGGYIILDKELVSRLLGTMVTYLVVLLQFRHAMHTPTIPWIYHPTTSTNTT
TNELDMGIITSTDGYKY
>CsplGr3
MAKSEAKEALGNEQQLNVFTAFLPMVYVSRLLGVFPLSYVSRGARSGDWNEDRKGRRGGKGGRRPSYEPSRV
AMAYSYVFFLSVASAFAATLPRALDAFSVFGNEALDSGQRECVSGLVLLLAALGALADLHFAAVGSRRLGRVFSAV
ARVDRKLAHLTVACGGGPGHRRLRRVAAVQVAAAPGIVLLQLLVDHHESAGAGRPSYHYLMAFYLIRLTSFTLEQ
QFVHVVLILRDRLRLVNEALGAILARPEVPDVLGSRAVLGQREVEGAWGSARRRRPRRESRIVLSAVTLKEMRMA
HFWLCEEANVVNEVYQMPVLATILALFFDIISGGYFLLDDVLSGSTKITAYLLSVLWVSQGTLRIGCLSWVAWSTSD
TAMKAKRYILRMREMGMAPAVEEELKKFLDYLLQGKIQFSLCGFVALDMAMAQAVAQASTTYLLVLLQLRRAE
>CsplGr4a
MDTRDDIYYSFLPFYYTSRFLGVFPFSLERDERGRILGFRPSKPLKLFCVLLFILNFSLFVLFGGSVKGGLTFQDGV
LLVQEFPSLLIQVLQWSRILTVFGFLVRPCDEENSLFRSLQHPTFLSSRSLGSRGPLKRRALMHSAFVLAFPWACF
WIDLDSLRPMERLPYIPVYIAFAQNICALHQFMFLLHAFGDRFDLIRDALRTSSSSERDPGGSADTWARARPLPTV
VGRLQRSREFESSEVMTLDRIEEMRSFYSVLVHERSRMTSRYSGTTLISLIGLYVSMIGNSNNILGSSIRQSDISLG
HSFELGVTIVVLFILCWLPRETIRKARKAGPLIVRLMRRELEAEAKEELNIFLMQLSQQETRFNIMGLFYLEMTVLQ
ALTQAATSYLLVLVQFQSQ
>CsplGr4b
MRRKAMNVYSAFAPMFHVSRALGVLPLVSRKSHHGVTTTYRASRLAQACSFSLIGTLAVVVVCSWEGSSRLFNE
LVQDGKDSHKRLLVNKVEYVFSAISSVGNLFFSMTCGKRLADIFQTIDKVDATLRFPTVVPRDMRARQRLVRLLTTI
QTVAFLCLWALAFAVDLLSFGKGDPRYWCMTPFYFLRLVTFCLEQQFIQVALIFRERFVVINDTLEGASSAPCFPW
TVSSATTAAGAKGSGFPANGGGVRRDRSRVWPLGAVLGKEDRRTCSVEQIEKLRVAHETLCLEVKKVNKAYELP
LLADILMMFFDFIAGGYFFITDLLDEGEFTSWGSSCSWVMNSWFRIAFLSWVAWSTSGKARKAGPLIVRLMRREL
EAEAKEELNIFLMQLSQQETRFNIMGLFYLEMTVLQALTQAATSYLLVLVQFQSQ
>CsplGr5FIX
MIHIRERIQNIWRELIWPLSFGDVDSLHSSFSLLFHLSRIMSVFPLNAVDNGKFHSKCSRYAKSRVLLVYNLGASATI
LALFAYLYNHMLKYFGYILHQEPKLFRKVKFTTTLRLFCFMLVVFSGSFLSARGNAIVNVFNSLSLITWKLMQSDAM
GHIKKRLAKVKICCLLMVCFFVVSRSSLLLAFSNSNKNHIYYLYLISNNTTAAIDSLMEYQFVFIVLIVREMLDWINRA
LDNIIRKHSDTEGARSTIFMVRTMLCPLHRSDDRDGDRAATRLVKDLLEYHRLLCEVHREASALYSVPLLSNLVSA
STELIESAFSMSSGIQKKADETRSLVLRLRRREMDRELQEELEIFILQISVSNPSCSILGLFTLNMELFYLIIRTCASY
LLVLIQFQEV
>CsplGr6
MKEEGFCRTLVPVFVVTRFFGVFPFVLKRNGENTYVLSKTVEMMTLGAQFALLASHLYFYPTAKVGEVAFKNKML
SNAHSMLVLKMEVFLFLIEWLGLVTYTIRVDLVWSIISGLFGVDRFLKQFDRPAVRRANKSMRLQIFSLAVYFGLDS
LIFFGNLNTSDVSVLIHLLSFVAIRMMLTGVRMKFVLIVGLARDRLGIIATLLENDGRKHRGVSRGSWTSQKRDSRR
RGLVNLNDPITRLNGSSHLSQLREEFSRKMKNTQRLRVIQCCRMCYSAVAAEMDKASSLFGLFMLSTVTSVLLELI

FTGYHVFFSYTVRDAIALLMCFNRGFTSLLHIVCICWISGSVVSKAQEIRIHLARKMDRGLDENLKKEINLFIDQLSQ
KKVAFSVLGIFAVDNALLLTMGEVIVSYLILLVQFKP
>CsplGr7FIX
MKNLNFKTSSCKAAMNRKKKVCDIFWTMRPLFLLSRLLGLAPYSISRRNARRDCPLQIYGKVIIALIVAVNLKASIEL
IHEKPRVQNQQSKDMVLLTIMGYIPTICTSITGLSGLMTSVVLRGRFARLHEGINRAILRLGRRTGLRKLRVMIGVC
CIIVISHMANRIVLQLCGIVTTNANVNPEYFVLNYLEDLIGYTTTLHFVSILKLMSYLFRRLNASLDKLFCRKKTLLAF
LDEDLHSTLDTNCSSEPQINKIIHLSEIHRDLRKLAHDLNHIYGVFLLFLMLGNILRTVFHLYVFAVILITGFHIQKTFLF
ILITRVPTRFVVMTYLVYACRDVREAAMETRLYVSKSILRTNDPRMTEELQLFSTHILQHNFKFTAMGLFTLNPELIT
SVISSVIANLIILIQFHSTEGVLTADINLMFNFPSNGILTSKQSLNHSIH
>CsplGr8
MEACNSKTHQRVVKVFRPALLLSEAIGVSFLTADGGRCVRLLHRLYGPALLAVVTLAFCDSLFALWDHIEDIRSRY
GAIFKWNATFFIIVKTFTISVMAIAVHFTRRSRLSDAIRKIVDIMDEMGHAGSQEHPGVKRMVLLAWVISLAQLALLT
TWTAISWPTALGDTFSASICLLYASLGLCNICSQYYVFLLLASRCLFLINNEMDGVVFPREELDAPEEGVRWFETG
AGRSRIGMIRHRRANSGSSSKSEACKRVRFAYLKLYEAIEVMNSVFGPQMLGLVIVTLSNLTFFTYWMCNEGVWN
DVFIIFTLRKFASNICYVATLCQIVATCQRIIEQTDETWKHLHEGLLRNSDPETRQELEYFESQLLQLKLRFSACGFF
KLDLRLMTTMASALLAYIVILYQFDRNSKR
>CsplGr9
MKQRQAIRARRESALLGINRVFQPISWTSRILGLSLATIEKKQVTKLASRVNKLTAPFVLVVLLLLQCTIAFMSCTRL
NKYKWLAFNEFKMRTRYILSHLIITIDLASIAIFIPRRFRAGKLISDLVNIMAISWRCQGNEAHGMRLERRLVISLALL
NPIVHLGWLGVWIPFQLPFLLYGLTVTMCYGSAAICTLNLQHYAFLLLYNSCFATIHKRLATLDPCTQRAHRSRSRR
GLRVIRDLRKAHIALFEGIGEVNSLYGPQILAMWGSNLVLFTFFFYWFLNLRKTSKTFFPLNFQIAAVCGLQTLIQIS
AILLTKMINDKKTRLWILIDHISAMEADRGLRYELQLFGKQLINLKIKFSLCGFLNIKVKLITAMIAEMMTYLIILFQLND
AIPRPRVDDTRHVNSARSNSTDTFKE
>CsplGr10
MEIPSSHPKFRQCPEIIGVSHILKPILLTSNVLFVSIDRYVSGLTKSSSVCKSAICHFPAPLIFTIILVNFSVSMLVSVVD
YTRPESPEVKSEETPLQATSAVVAVAFSTVGGPLALEFYAFRRARLSTAMKLINQVLSGRSREEPAGGSPPSAWE
RRAVHAMLAYYCSIVLGSPVIVFCLQKLSVFLVLQYATQCFLIAVECAVCSQYFGLLLVVGGLCSRINGDLRRLLDR
ELPPELPKRASQKMVPPSKMRKRVQEVKESCVWNSGRWNRRRRGGAESFGRGGFKRLATAHLRIHGVLRELS
AVYGPLLLFVVLSLGMYLTLTVFWANVTRTSIMKSPLMVFNRVCIVLVQAGTLIALIIASQRVRNEVRLLGKVLTIITM
ESQNILLKDQANLFSRQIVHLKLVFPVCGYFDLDMELIRKMATSFLTFLFVLLQLNEENRIDYNE
>CsplGr11
MPTKAKTLLWAMSPVLFVGRALGLTPVPLGGNRDREAKDFLLSCFLAHSALTLVVFNVAFVYVTSKNFQVAIDEYS
EQRKVITIAWVSADTLVFTIYFPCVAFRQRALHDLLERFDSDGSALGLPPNASHARRLRRQCLAGASLLVVFALAY
LASQEFATDNVSLAMKLMMFYRNVTMMFAELIYAVVLCSIRNRLAELNGMVRELAMESGSDFKTVNRSDYVDIKP
LHHTTDYRISSKHQLGRGGEEGSEKWRERGQLVVRKLARHYLSIYQTGRSIDAIFGFPSLLYAIGAFLGAAFQSFFI
IIYLLEVYANVNAGVIILFPIPWKVAVLVVFAVLVYSSQSAEDEGRKTSEVVALYQLRTKDHRVRKELQIFSAQLVHSE
IKFSAYGFFDLNYGLFTSIFTALLMNLVIMVQFHLANF
>CsplGr12
MTMTRGPQRHLKMDRGGPLEELRPIFAASRVVGLAPIPFWSRRALRGPMGSATLIYSVVVMVSFAGSLALCFPTI
YRRRMHAASSAAERMNVLISLLWTFVPCFFAVVVMATYVSRRGRLRGVLVEMDRIHFMPQPWRIVQGYKTPWY
SLVLQVRSRRGGMTHDELSNRASCVSPPTRSALMMMESPSFDGVRSHWSSTTCRGYASPGGGRRHPDPRDR
DVPMADRIRMLAEMYFSIHGVAVELSKIYGPSLVVHSAMTLMELAFQLFNFAVGIHRGADLWMRVIAGLFWTCPFV
AVFLAVLATCENTSREADLTRSLVTEFYYLSENNGVRRWLSTFSDQLRHMHLEFSACDFFLVDFSLLLSIISAVMTN
LVILLQFHLSQNI
>CsplGr13
MSRKRSRMKNQEWSVYSLIRPLFLFGLIPILPPFIAEMRKETEKTMIRKRRAYPVPMSMMRAQAYSIILFYGLGNVI
MLVNLSYSPRRSLGAFVHHVWPVFDVTLLLVSSTVLLINQTRIGDALRRIQGTVHTIRGSKGVHRRLKASIWAQGL
GLWTILLMGCAHLGTTNAFRERVFGHVLKFASHLAVTAITLQFSTLLRIIHLNLKCLNGHVSDLLGETTADAVAERR
RRAGGNGKRGDWSHPVASLESPVKGVSVARRLRRLALLHLSLCGMARELNGIYGLNTLVHSLQILMDLAYHSLTI
AERIGGIAEDFSTWRYLVNLCIWNGPNMGSMIYSVRQCNSTSREIEFTMKTAIDFSLKITKNRVLREIRRFCNQIHH
SKSKFSGAGFIHLDYSLFVKIGAAAFSNMIILAQFQSYVGD
>CsplGr14
MQAWVTVAPGETSTEAYRFGTRDKCSTKQSKRKHSGGNRTDHSLADQDVLRTLRPILITGRVFGMTYYPLQEDV
KRGRYTPRFYGALTLLSYGVLVVSIGTFAYIIPQYYYYGSRLVRGVDGSTSNGINMTVLWITTTTLGLVPLISSVFLR
TQINELIADLRNVHAELKAVGALEIRRDMLILGIIIHMATEFVTTAILMYSAFIYVEGEQLSRPILNCMFMYYMVSASV
LSRTLFLSILRLIGSGFRSINDILEVFRDQHESGTRLTRRPEAVDSLRRMVDGNRRRKKLRSSPKRFGESYGTKLR

RLAFLHLKLSHICEKFMSVFGPHLLSILLATIVCSLCYGYFLFQWAMGIFEVEHSVIIFVVFMKVKLLTISMAIAWLCG
DVSSKGENTGKVLSSIMIKVNDGPVKDELRTFANQLLHSKIEFECCGLVKIKPSILTSMTATIVTHLVFIIQFQLSALQ
TGNGPQNVTREGNSVTTPSYDSSSNVL
>CsplGr15
MAAWITVTPGDGSREAYEFRPRDQHCKKEFKRIRFDGSTPGDPYTGGDVLCALLPILIIGRVFGMPYYPLQEAKR
GSCTPRLYGVLNFISYLKVLLSFGMLAYIGPHYYHFGLGLVRGLDRSYSNAINITVAWISTTTLGFVPLMASVILCTE
TKELILELCNIHAQLKSVKAYSMRRSRLKLRIVISLTVELISNAILMFFVSIFIQDRQSSRSLVDIVFMFFMLSVSMSSR
ALFMSMLQLIGSGFCTISEIVLDAFGDPCQGGNRPRRLLDKAVDRSKRTARGTREGRKQAKDPKNFGEREATKL
RQIASLHLNLCHVCGKFMSVFGPHILSIFTATIVCSLCYSYFLFQWALGLIEMDNVITLVILLVFMKSKLFIVSMAIAW
SCGGVSRKGENVGRVVSSVMVKMNESPIQDELRNFANQLLYTKIEFECCGIMKIKPSVITSMTATIVTHLVIIVQFQL
SASQTLSSYQSKPNEANVSTTPSYNASCTVL
>CsplGr16
MLNPSREKPKGGEGVKWKPFFLEVIGRPIHRDWQTVQVSVSRCGNSMSHSRVVLVSERAGRTSVARNPLIVFS
QLLGVCHYPSGARSLREASRLHSAYRVSVLLATAATTSAFLYYRIFYLYGQFKTNSSFTRVFSTFCWWLVFIVSGA
VGFFLMLSRSFLVVERFRGLQDAGGISLGSPSPDPASGATGRRRRWNFVLQATFILSFPVVELLRQALIAAFMIRK
YPNNLHYSPTVWLATNMTIDFQLATLMWMVRRNYARMNSAVARLIDEAKSRAGNEEFAAGAPSKGPHYGGDWG
GIPRDSCPRRALVRRMAMLDLKLHNEASLTNDVFGLYLLVRIPMILILICFHTYYYLEVVIGKAQIETAYVAAMDGYS
WLLSNVVGLVLPVLSCQSASRQAALTRWLVAECILKTKRRLVRRELRLLSAQLHHTKHRYTALSFFSLNCKMMTS
VLATLVTYLAILVQFWQINPN
>CsplGr17
MTAPPRNALGALTPILIISRIFGIAYYPLDGGGKGLGGGLQRTSIVPVAHSLLVLFANSYLATLVIPNFSQVGQMLTAA
GHHSIVNRLVMIAFWCCSWVMSSEAIVTSLLRKDKFRELIDGLCAVESRLTSTRRHRYTRLIIIVLLVFEFTVTALLTY
LLKYSRDVEPLPLTNLDLIFFTYDICVSIMSLIMPTAFLVLFLVDFTSVSEGLRAAVVASPPAEGASAGRWPGPPRRV
CRRARVEASASPGERRRGGRRRLGSPPRGRRVRSLAEQHFLLCELTLAFTSLFGLSMLLFTLGTFSTMMITSYFI
FEWFVEMVHVRNVSLLLYGVGGVVSMGFVLVTLTILCSKVCEKARNTGRCLPEAIIETNNVTFRDELELFSRQILLT
KVKFKPLGLIHFNIRLLITIVATFVTHLSIVLQFHFSALNK
>CsplGr18
MDSHSSVFIFSPILCLPLPQGEGNSAPHRLHRALSWLLFISATIATLVSLPAYASGVAVEQESQALPIKEGINTIWWV
LVSLLGFAAFSLLHFGRSRIASVREDLESLPVLLGQGEPEKGDLILREKRHLKVWILCQWVFLAAVFSLAAVLHIYAI
NAEHKTFNGVLNVAGIYWLMTTLVVDLQFSNLSLLLVRNLRLLGECARGALRDGGHRPRGKGGGGGAGDAGAQ
ASSPRTRKQSPSKLIGDLTEHCLRQHRLADEVNRIFGPFLLVQVPSNLIQFTFHLYNYAEILIGVAKLENVLFSLTEV
LVWAVSYGFGLCAVISACHAVTEQEERMHDLVADNLAKVEELELLRKLRLFSSQLYLTPLKFNACGFFTLDYPLLVS
VAAAAITQLVVLVQFKQSSFTRI
>CsplGr19
MVRDAQHEIDRAFQPLTRISQALGLVPLHTTGRTHKFLTNRSRHLIGVSRKRFTHRSIMSKVYSITTSSFFLGISAY
SSHTSIVFLLSLDVSSLPHISEVVNFLILNICTIAKYIFFLFIGYPSLAEFIAALQRLDVKYEGTGQIDLQEITTFVYTCV
KYYIVITLILSLLTFSIHQEYLISIVLIIGKTVSYLINYSIDLYYVSFVILIQQRFLLVNNLLSCFINDNESHNVKGRQKPNK
FITEIDSIHDSLCDIVDAFESTMGPQVLFHSISMFFYGTITLYYLLATAFGYINETKKYSEILILVVELAVTVFKYTGTVF
ECYKTRKEAERSADLVTHALTHEKWDKRSERELQLFSTQLLQKRVNFTACGFFPMDPTILTSMAAAVATNLVILVQ
FQMALSESKRSNVETTSISGGENDTNVIIASLAH
>CsplGr20a
MISKEQNPKYNVYEVFRPVFIIGRSIGIAAFPISKTEAHQGVRIGPVIYSAAAVTLCNLFFGFLMLQCRALSNLSGNE
FFESWSVVILCVVERLKVCEAVITPFLRRKELHKFNLEMNAVMAFLKTDCAQIKRAVVWEVVIIFIDIFQRIFGIIREA
WIYNHALKVEFMMLVIIRRIVVLPQQTYMWNMVLILRRLFESINKEILSIANEECRVLRDSKRRESRKCSLTKRSKL
NSRKLNRLISLHFRVTRLCEKWNSIFGPKNFLIGFNHLLSGISVLCQNFTQTTGHSTSNFGTRLIEREIEELISITVVT
YACSWTKRNADRTGKAVSEALLKIRDHEVQAQLKDFSIQLLHTKVNFTACDLFQLEISLFTSMFSSILTYLVIILQFQ
QSNMETLHS
>CsplGr20b
MRTGSTDNAFDVYYLMRPLIIVGKAIGVWTVPAKGGKGSSKFMLSYSTLIFFATVALLFPLLKNSQYNSSRSTENA
KQDSINEIFTTIVVIINFITTWVHMVRSLFRRRKIETLVDGVAKLASDLECNCSFLKRRIIFYVLFLIIAVPLNLTVMYLS
NFRYISLHYSIMLGISLILLICNINFFYEYTLLKLFHTLFKCINSHIHTNLDSENINQGPIVVYGWESKTLFLTSKHNIFTI
RIQTLAEMHYRLTELCSQWSTMFGIPNMLFTINNFLITIYGIYLSLQIILSLIVVKNPLSLLIHVLIETSSEMLKLFLIISS
CTATSKEADRTGKAVSEALLKIRDHEVQAQLKDFSIQLLHTKVNFTACDLFQLEISLFTSMFSSILTYLVIILQFQQSN
METLHS
>CsplGr21
MKCTQELNMKTKSKRSESIYESMKPLLFVGRIFGFGNIPFRVTKSNKEVTKYLLLYSFLLFFLSIYFSIAVIPINVERS

LSVSHYKNQQYIQVIFLNVVTIKSSIIGVGGLSVFIFQRKKIRRIFDEISRANHILKGDCFPLKRRVLIEMFFAAVTTIIF
GITQCKAYRKPVKLISSSINWSFCTVYYMYISTMIILIRDRFECLNQRINSVVEGLRVESGGTTTGWDGVRSGPGR
GWKDRNVVKSLDQFSDVHLRLTDLCSQWNARFGFLNVMMSINCFLEVISFVYLVVRIDQKLLIVQDSLCTITNGLT
WASSAIFWFTMIVVACTSTVNAAENTRKVVSKALVKTTDQEIRAKLKDFSIQLLHTRVAFTACDLFTLDTSLLKSMS
AAIVTYLVIIIQFQLAPTSTT
>CsplGr22aJOI
MDSKHSTSSEFFHSIEPLFFVGRIFGFGNIQFRVNKTTKEVPRCQLFYSVSLFILTTLFSIAILPINIQKSIFMSRTKN
QKIVQVIFTAISTLESTIAGIGGLAVFIFRRDKIHGMFAEIARVNHDLKGDCFPLRKRILLRVFFVAVATTLFGIIHVREQ
KQVVKFVAAVMDWILSAVYYMHIFCMLFLIRDQFKRLNQRIQSVADGPQVDSVGTSTGRYVRDGESGSNAHGLK
DSSVVASLGQLSVVHIRLTELCTQWNETFGFLNVMMSTNGFLQVISFVYLMVRIELKILIVENPLRTMWRALSWST
SKAIWFTTIVVACSATAEEANDTAKAVLRALVTANDEQLQMKLKDFSVQLLHTKVNFTACDFFEIKKSLFTSMGATIV
TYFIIIIQFQMAAEPPLLETKSSENDTADY
>CsplGr22bPSE
VRRLAYEHFQLTEYCSQWSSLFGPNCVMISVNTFAAVVSFGYTTVGKPLNLVHLKNFEIEIMCAFSWTVATVVAFIT
IVQTCSSASSEANDTAKAVLRALVTANDEQLQMKLKDFSVQLLHTKVNFTACDFFEIKKSLFTSMGATIVTYFIIIIQF
QMAAEPPLLETKSSENDTADY
>CsplGr23FIX
MCQERANGVVWAMRPVLTLSRISAVATFPFDPDLKGLRMRIISAVGSIQRALSVVAYVTLQSVLLSTPDAGGSMTL
LTRTTSILWLAFNALTLLVTFSTTAFRQRALQGLFTRLSSLANGDPMTNIPAGHLVSIRRGCWLQVALISTVVFPSLM
FCLLVWANIPSLISFFISTVITMNVNALHITALHVLLKSYSWYNEALGRSAHGWNAARFHRSRRRRPIDAGRSSSG
VSGVVIRLARQHLRLFKLSKDINKFFGISMLFYSSTVLVELAFRIFYIIELFASSYYLNGMSSICIPILMYSIPYSSSFFV
IVIFCQAIQEESRKASTHLTRHILETNSESLIRKLRLFGVQLRHTKINFTAGGFFALDKTILVSGLAVLISNLVILLQFSIS
YEA
>CsplGr24aFIX
MGKEYHASKFHGLLGAMEPLWYLSLLAGVAPVPQGGKKSALLSFACVAHSSLSIAVIIVLSCAYLPEKITYVLYRKV
NGAQLSALAYAMWYLLCCGTGVASHALALRMGPLQNDALRMLAEAGRRAADRSEISKSLRRTRGLCAASTGLTA
LHICALVIHGTLSGDLGNGGAGSLAAFTWATISTLVTNLRFFTLAFAFCQRVAMLERRLEGLAPRGAGGARPPPSP
PHLGHLSDPRRRGDDAAASVVRRVTEERLQLISATKVINRTFGHFVLAELCSVSLMLTLNLHLMLMAYLWLNMRD
SVALLLLDFFLWTGMHALGMFAILIISEIVLRHDERTRSLAMELLIKSTDHHVRGELRLLLSQMQHGKIYFTACGVFR
LDCSVIKSMVAAVITYLCILIQFMPDSESDFYHNQKES
>CsplGr24bFIX
MAREGTTTSKKDLLWAVRPLYYVSRLVGIVPCYHKKRKKEALSRGLIVHNSISLIVLLLPIAVYMTNQLIHVLLYSKQ
ESPDFFVFLDVLWYSACLSTGLFSYAISFRRRRLYFKIFRRLEKLERGEVGSVAWETHDGTRNACSSATALAGAQL
VALFLNESLDPSGEFHRSHPCLCFAGLWSTVAAVVTALHFFTLAFALAQMVDVLNRRLAEMSARGPTGVLNPCVA
ASPDLRRRRSTLEHQLRISGVNGEASEVMRVASWRLELLSIVRDVNRLYGGFFLVFSLTTMMLTTMTTHLFTDNV
FGVTRVGIRYLVVDFLTWAGSYLAVTIGVLKQSERDERTRSLAMELLIKSTDHHVRGELRLLLSQMQHGKIYFTAC
GVFRLDCSVIKSMVAAVITYLCILIQFMPDSESDFYHNQKES
>CsplGr25FIX
MSAEALSLNLWMRLTHQINQQIAGFQMKEGKRDFIWATAPVMLTSQILGIAPIFARPRSLKRERRHSAYAIASLVTA
FSVSVVGLRLVYTTWMTLLNPFMDTMNSVMLSFRMIAMHALALSIVLSLVAKRGLHRDALRRFSTTVAKDASLRR
GNLSRLRLLSWLHFSLLSAVSVASVYRISGFGFFHRVAPDFLPIGAFYYLYPLIVSMLVDMQLVCILWPMSKCYRRI
NSLLDGACERWRLLARRNGRRRRRDAVVRGRPGVEPDAAGSRVACFREASAVRDLAARHLALCQLFRDLNDVY
GVSVLIQSLAGLMEVASEGYFLVEDFIGSNEAVAVMEGLQFSRMASWTFPCVGMFLAILGTCHAVSDEAGKTKNL
VISHLSRTKNYELRLELRLLSIQLEHTELKFSASGFFTVDCSFLTAISSAVVTHLVILVQFQTAVSHEKQLASWSNNS
LEFNFTATP
>CsplGr26aJF
METTAIDSRLLSLFWATRTVYLPSRLVGVAPFAQKANRKEALARGAYAYSIACCLVMSSLAAFCFPKQVLTMAYYF
NVDIIDFNVYICMAWYASLLHSGLSCCALPLRRRSLHFEIFRSLAEADVSMGLGDDSAFLRRSRSVCTAAATALGV
GHFSAMLANESIDPSGDGRTSPLGMFLLGVWCTAITLLTDLQIFTLAYLLRQRVGILNGCLEGLLRIGQGTPPGTM
TTAPGDSPSLGGRDSGGRLSPKDVVRLVASVRQKLVSVSRDVNALFGGSFLTATAATLIMSTMNGQLLVGNVFGG
QTFAPMRYLVLDCVTWTGSLFTGMIAVLVSSEGLIKGDERSRSLAMELLLKSTDRNARREIRLLISQMHHGKIYLTA
CGVFRLDCSVIKSMVAAIITYLCILIQFMPIKVEDDPKNQEQENLIILNGKL
>CsplGr26bJF
MTRRKSSSPQLNELYDAIRPLLILGRLVGVDPGFREERKGEVLRMIYIAHSLVSLATLTALTGLYLPANIVQILSFYED
RAEFDINSFTVILWYCLTVSTGFASFGLRFGRGGLQNRILRTMAKVDRAVGCPETLRRARRICAASTTLVSLQIAAL
LLYETLDPTGEAIVNGAGIFIAYAWFTATTIVTNLRCFTLVLMLKQRVSILNQHLERLVRAEGGHTSAIDLSFAAREAR

RANRLVTNVRRVSKVWLKLLSAAKDINKTFGDYLLLELCSTCLMATANIKIMIEIVFGMHQTFEMEHTIVDFILWIGA
HTTGTMGILMLSEIVLRCDERSRSLAMELLLKSTDRNARREIRLLISQMHHGKIYLTACGVFRLDCSVIKSMVAAIIT
YLCILIQFMPIKVEDDPKNQEQENLIILNGKL
>CsplGr26cFIX
MRDEAKTTNCKDFRWAIRPLYYASRMVGVAPCNQKKKKNEALSKSLLAQNSISPVVVLVSFSVYLADHFNYTNFF
PREPFSEPVLYLIVLWYSACLSTGLFSYAIPLRRRRLYFKIVRRLGKLERGGVGSAAWETHDGTRNACSSATALAG
AQLVALFLSESLDPSGEFHLSHPCLCFAGLWSTVAAVVTALHLFTLAFALAQMVDVLNRRLAEMSARGPTGVLNP
CVADSPVLRRRRSTLEHQLRISGVNGEASEVMRVASWRLELLSIVRDVNRLYGGFFLVFSLTTMMLTTLMAHVFT
ENTFATTKFIDRGYLLLDFMTWPGSYLVVTIAVLVQSGRVLKCDERSRSLAMELLLKSTDRNARREIRLLISQMHH
GKIYLTACGVFRLDCSVIKSMVAAIITYLCILIQFMPIKVEDDPKNQEQENLIILNGKL
>CsplGr27FIX
MEKRREAFNRAIAPVMIMSRVLGIAPAFARPRSRMLGRLHSAYAVATLVATFAVSLIGIPLVFTNWMNKISSRMTVM
DISLLVTRTFAMQALALCSVLSLVVKRGLHRDALQRFSDCMAKEARPRQGDLCRLRLLSLLQVLLLVAFAVVSTDR
FIRIRKPTTAYTKLSISILFFLYPLIVSMLVDMQLVCILWPMSKCYRRINSLLDGACERWRLLARRNGRRRRRDAVVR
GRPGVEPDAAGSRVACFREASAVRDLAARHLALFQLFRDLNDVYGFSVLIQSLAVLLEVAFQGYFLVRRVVSQET
PLAQIYGFSGRVVLWTFPCLALFFAMLWSCHAVSTEAGKTRDLVTGGLSRTKNYALRLELLLLSTQLQHTPLKFSA
AGFFSVDYRFLISVSAATVTHLLILIQFQTSDGPAC
>CsplGr28
MTGTRNIFWALQLHAVVSRILGVFPLKNAPLFSSSFLYTGSSLIIVAFQSYYVLFVFPALFVLKSDSPSNFNGFLATS
WLILSIFLSISANIFSLRYRRTIHSILLTIYKMDTDEFGHRVQPSLARERFNSWLHMAAFSVGVPSVILMEKYNPSAR
LHNPLLTIVVWVSVWTTLSMDLQFRAFLSAIKDRLRLTNESIWRLSWASSGFRRTRLAGVGERWAAVDLLFKRPS
LGDRKYARSMCKLHHDLRRLAAGVNEVYGSFILMQTAANLFQFSSHIYFFLTVIVPEALADSRGVWYEFFVLLVW
SAPYIVGLTSILVASDGVVREAEFTGVVVSEVLLNVREQRLRKELFLFSNHLVNEKIRFTAKGFFPLELSLMTSVTAT
IVSHVVVLIQFQRS
>CsplGr29
MPDPATKMHPMSRTLFGSVREQLGDLNRSLRPLNLVSKAFGLVRLTSDPSDQKFGWVNLVILFATLTISAWYLPRF
IHNHLREKDCSIMALVNCAWWSSIHLVGLINMLVPAARKDHLEKIIFFLSGHCPGAIGASQSDAETLEGARRWAWL
LALLVPALYVILLYNQLFCDRNPFNELENVHFLAVDYFIVSAFVHCTSYFFSYHTISSRSPPRFAALNRSLVVTMVLG
SALGRSSTVREGIEASESSPRMAKRSEFGPGTTQSTGGPLHPPPPRATVFGLAYSHSRLSRAAKHVNKAYGVQI
LSTSAFRLFNMTVGLYYCLNVFTGDEKVERVWLVFIQATMWLGFYMFVLFVTVMMFEAVSEQARLTAMLVTEAIIN
VQDHRTRRELHHFSDQLFHTNIKFTACGFFELNFGMVASMLATVVSYLVILVQFHQATK
>CsplGr30
MDIFKALFPLLWFNKILGIFPARIELYKPEKYKSGYRKALSILYSLSIFSTLVFLSFFVDESHLMVLENSLTNSREALVL
KVIKFKVRCNVVTCWISYFIILLKSNSLLRLLSRLAKFDLELSLCRNSYTTTRRVVVFQMSAVVMNVLILTVPFQNAP
EKGNGGGLVVFDNIVCLVVKLSLASLVFNLFFIFRSRYHALNERIRLTVNQSRSNSWWVRGSNLLRGDKMVRVSD
SISRLSAMQRTLFTLLTETNAVLSEVILLHFAVTFVEITSLSYISVVFFDDGVQTYRKIRMFCRLLFVAAWTSAITITCG
CVRRKADQTGVIVSEAIIKIRNRKAKRELRLFSHQLLHTKVEFTARGFFTLDFGLLTSMTAAIITNLVILVQFQYSDGI
PGDNSSNCTTYYTIKSFDNVTDMPF
>CsplGr31FJ
MDVFQALFPLLWFSRILGALPVPFENLKSGKSYGCKMQMFPIFHSVTAFAVLAALALYTDVTYFRRVATTLKTRRE
DYIMKAIFFKVICSMAASFISFIIIACRSKRLFRLLKGLADLDLSLFRDKNANRVTRIFVGLHMSILCLCLLVLGTLLDE
QPTAHDRKHWLEVHDNITCKVIKITILSQIFQISLLFHTXYDALNMNLEKLVKMNPSHVLYDSKFFFSKGDRSRHNS
KDIRRLCYLQHRLFTLLSELNAVFSEVILLQFIVAYVEFTLLAYTLIFVVDKRVGVRSMIRIYIHLILHAGWVASITASC
GCIRKKVRWTDQTGVLVSEAIMIVKNNRARREIRIFSHQLLHTRVQFTACGFFTLDFELLTSVTASVITHIVILLQFHI
YTKNSSSIFNETFSKQP
>CsplGr32FP
MNIYEVLWPILYPSRILGLAPFSLRDPESVRSRRKLYFTYGTLVFVASVFLTVYADTKFSASLWFNDQGERNMLTNV
AIDWLANKKICGAACFTSFNLRSGCLLAVLRRLEAFDAEVRAAGSGZPARSKARSVALHAACLSAYMAAFCFFWT
RNSNDRIRLSFCGNVIWMSCMLLLQCQATSVFSSLDLRLSLVSDCVRRVNGWRGGEZRGGRSSRVSGWYNELF
KIMWEANRAYGEVILLQVLAFNLDSSLLIFHFLQYLHRTFLWETPCSCWFERFTICSEYFQSCRSVRKSKQRYKDL
ILNIAIKZVIFYFRNZARHTGVLVSEAILKSKNNKVRRELRLLSHQLLHTRVQFTASGFFTMDFRLLTSMTAAIVTNIVI
LVQVHYSGITSDETSYDCDKQCPSIDIAMAAQNQTLIFE
>CsplGr33FIX
MDVFEELRSLLWICRLLCLAPFPFHRGLGDDVLTRMKGSLCCSVAFFLVAVALTLYEESYLIPNFFHHVPIFDTFLKK
LSYLIWEKHFKVMAVICVTIILVNSGKIYCLLNKISLLDSEFCKFIRTESHFDSSKRETIIHVLMLMTLLPCFYYIVWWR
SAEKDLVKFAMNSYWSFVLLASQSQLINVYRIVVGRISLLNNFLSGIQELLAASRQSPKEKHLAKQKLRISNCNSS

HLESLVNLSACINVKLRFILKDINSVYGLLVLNQVAIVLSGSAVGIFLLLILPDFISDTFDYIYVSMRQSLFMVGVVLMA
AKGEEATRQARHTGVLVSEAILKSKNSKIRRELRLFSHQLLHTRVQFTASGFFNMDFRLLTSMTAAIVTNIVILVQV
QYSGITSDETSCDCDKNCPSIDTAMAAQNQTLIFE
>CsplGr34FIC
MVVVPLKSTPMVSQIEPLLRVNSLIGLLSWNITKNHSKHCRFSWASFIYLILILLSLCTSLHMFYTTLFPDLHSYANR
FVFLFLVSMQINLGSICCTALVFRQVHVLQTFAMLSKLENVLEEREAIGLYFPRRHIIMRLIFILFGSGQYSLNIFLRG
VFTGFFSFQFLYFYFCIFWTFVLWMQEWQFYIIVILIDRYLMTFNSEIREIYRNHDSEVRLQSAAVGSISKYWEGRV
KRWKTLQMLIYQLRKRTNTLYGVSNLCFGTYCFLNFTIFLYMLCDSVISSEEEKISFFDLAGSLAWLSPYIVTIKVVT
AACENLNKKLHLFSYQLLHTKFRFSACGFFSIDYNLLTS
>CsplGr35NFJ
CYIVSFRFTRSRQSLRHIFEDLTACKVLLRCGQQTEFSGVRAHANFQILFLILLSIVCVLNLVLNYQVFNPTNLGFTY
ILFTNIWSIICACQWGQMGTTVHMLFVLLRILNRHICNSNASHSLVEKTSIYNAEYGDFILPFLKNWKKIEWIIYRIFR
NVNRVYGFTVLILGSINFINITLLLYYFIDRLIDSQALTFMGQEIFIFILLRAIFYLTPFLSIVEVCKRTQNEADQTVVFVS
EALLKVNGSKARRELHLFSYQLLHTKLRFSACGFFPIDYTLLTSMAAAVVTHLVVLVQFQLSSKDRSPCICPFTYGT
EVPTPPPTAHS
>CsplGr36FP
MSDNAQDGILRNFGHLLILNKAIGVAPFSQSGRTTNHYCIGLIYPILFGFSIIIVLLQLASKNFLGSTLRGTLMVVWDS
YHMTMCCVVSFHFSRSHQSLRHIFEDLTACKDILLRRGQQTEFSGVRAHANFQTLLLILLSIVCVLNLIFYYQEFNN
TKFGFTYILFSNIWPIICACQWGQMRTTVHMLFVFLRILNKHICNANASHSWLEZSSVYNAEYEDFILXFLENWKKI
ESIIYRIFRNVNRVYGFTVLTLGTINLINITLPFYYLSDRIIDSKQAVTFMGZEIFILILLWTIFYLNPLLFIVEVCKRTQNE
SGRPVVFVSEALCKVNSKKARRE
>CsplGr37FIX
MTISVNSIIGSCNVVHRSFGALLSLNRALGLAPSRPNIKRRQGCNISFQKYIHRILFSLSIFAQISGLNKAKYPPSSLK
EVLQVLWLNLQIFISSIAVYQVASSHQTSKSIFGSLYKCYVDFKKAGYMIKFNRIRAFTYFQLLLIILNIILYVIVVKREF
RLLKTDNFKLYLLMFSAFWSISCGCVWQLVVTVFAVTNNLLFVFGEYIREVIDSWQGVMYQPSNVSSIPTLQVVST
AVERWREIQINIYRVSKGVNSAYGISVLVLGILNLFNITFYLLFNLGKFHKEGLLKYEILLFSLLWTSVYLIPLVLIVGG
CDGVQNKADQTGILVSEALLKIHNHEIRRELHLFSHQLHHAKIRFTACGFFPLDYSILTSMSAAVVTYLVILIQFQISG
NGPPSCSSFFCNSTDESMSSTTPLK
>CsplGr38FIX
MPLNKYDCASNDVDRSLGALLSLSRAMGLAPCRTHGRYQKGYTNSLYYYRYNILFGITFLCHTLMLTNDGVISFT
SSLNTILSLIWTVAQVNIISLTVLKFSRSKDTLGYILEDLDRCCRILQKAGSRISCGKVRSVVNILMLLFFIVVINCLFN
FRKELRKHNDLINLFYTILFLVWSISYVCVWILMNTIIFMTYVLLILLGENIGDLSDSGMFFVIDLPVIPKHKVHVTPRI
VKRWREIQINVYRVSKNLNGVFGLAIVGLVLLNFCNLTLILYFIIAEITVARDTSLEYELLVYGLPWAWGCFMPLILVV
AGCNAVHAMADRTGFLASESLLKIQNHEIRRELHLFSNQLLHAKIRFTACGFFQLDYRIFTSKTAAIVTNIVILVQVQ
YSGITSDETSCD
>CsplGr39aFIX
MTMSMNSRLSSWNGVHRSFGALLSLNRALGLAPGRPNIKRRQGCNNSFHKYIHRFLLSLSIFVQISTLNNDNSPT
SSFNKVLEFVWFNLQIFISSIAVYQVASSHQTSKSIFGCLYKCYCEFKKSGYTIEFNRIRAFTYFQLLFIILNTILCVVT
VEREFRLLKTNFYKLFLIMFSSFWSISCVCVWHLVYTVVAVTNNLLFVFGEYIRDVIDAWQGVMYQPSNVSSISAV
QVVSTAVERWREIQINIYRVSKGVNSAYGISVLVLGILNLFNITFYLLFNLGKFHKEGLLKYEILLFSLLWTSVYLIPLV
LIVGGCDGVQNKADNIGTLVSEALLKIQNHGIRRELHLFSHQLHHAKIRFTACGFFPLDYSILTSMSAAVVTYLVILIQ
FQISGNDPPSCSCFVFNSTDKSMSSTTPLK
>CsplGr39bFIX
MSVNPSDSLCNDVERSFGALLSVNRALGLVPSRPNTKRCLGFNSTFCKCIYRILFSLSLFIQVFIFKSEIAIPLISSLN
GILEIIWIEIQVFITAFSVYQAASSHQTLKSIFDDLYKCYDTLMRASYTIECNRNKMFANFQVLLIIFTTIQNLFNWERE
FQQTNTDFLKIAIMIFSTFWSITCVCVWHLIYNILVVINTLHLVFGEYIRDVIASWHGVKHQPSNVSSITVVKVLSNVK
RWKEMQINMYRVSRILNNVCGISLIVLVVLNLFNLTFYLYFILVGVDKKELGKNEFLVFGLFWISGFLIPLFLIVGGCH
GVRYRADNIGTLVSEALLKIQNHGIRRELHLFSHQLHHAKIRFTACGFFPLDYSILTSMSAAVVTYLVILIQFQISGND
PPSCSCFVFNSTDKSMSSTTPLK
>CsplGr40FIX
MPLNEYDCASNDVDRSLGALLSLSSALGLAPWRTHGRYQKRYTNSSYDYLYNILFGITFLFHTLMLTSDGVISSTS
SLNTILPVIWTVAEVNIISFTVLKVSRSKDTVGCILEDLDRCCRILKKTGYRISCGKVRTVVNILMLLILMVVIIILLNFIK
EFPKHNDLINLFYTILYLVWSINCVCVWILMYSIIFMTYVLLFVLGENIRKLSDSCPFFVMDVPVISTHKEHVTPRIVK
RWREIQINVYRVSKNLNGVFGLAIVGLALLNFYILTLILYFIIAEITVARDTSLEYELLVYDLPWAWGCFMPLILVVAGC
NGVHAMADRTGVLASESLLKIQNHEIRRELHLFCNQLLHAKIRFTACGFFQLDYCIFTSMFAAVVTHLVILIQFQFS
GKNHSPGTCLVRSSTHKAEFTANSSL

>CsplGr41FIX
MSFHSSYSSYEDIDRYFGALLSINRALGIAPGRASSHNRKGSNHSFISLIRVIFFTFTVLIEILLLFRNEAILSCSSLNS
TLPIIWNELEMIVTSFAWLRVSRSQDRLKCIFKDLAKSFGILRKAGYGIKCGKIRTVANIQLLLIILVIIQSAFILVREYR
KSEIDFLNLTFLAVLSVWSIICVSLWLLMRTIIFTANLLLFDLGEYIKDLSASRKRRPSCELSNFTVKGRRLARVVKQ
WREVQKTIYGVLKNVNSAFGFTVVVLAALNHLNITLFLYFFFDGFIHGYSYCLGYAGCIVETMLWTCWFLIPLVLIA
GDCDEAYIMTNQTGVLISKALLETKDIEVIRELHLFSCQLLHTKIRFTAGGFFPLGNRLLTSMLAAMVTHLVILLQFSI
TAQNRR
>CsplGr42aFP
MRNTSFENRALKPMLGINAVIDIAPPFLYTRDKLLYRKRRVATYFGLEFTSALFVIVSINTFVTVATISMISKMVMFLW
VFVYISVGGVSVCVIALNLKAVEDIFREFIKLEZKLDGYKIIPMSDARKSVNFQIMLVIFNCIQLFLNLGYIGVEIEFMTI
DLFRCRLVAFWAFIIYVHSWQFSNKVVLLFRSLTNLNDMLVSLAGVNDYEYRKFCTDTSGTAAVLNQIKHLKKLHL
RTYQIFKKMRSIYGIPNFXIVNILDITFLLFYIFATIIIPRTPPWSSTQVTYVSLWVZSLFFLFCRVISSCHRTZEKAEKI
GILVSEALLKIQNHEIRRELHLFSHQLHHAKIRFTACGFFPLDYSILTSMTAAVMTHLVVLVQFQLSSKENSLCVCPS
TNVTEVPASPTTTLS
>CsplGr42bFIX
MTMSVNSRLDSCNGVHRSFGALFSLNRALGLVPGRTNIKRRQGCNSSSHKYIHRILLTLSIFAQIFALNNDKSPTS
SFNEVLEVVWFNLQIFISSIAVYKVASSHQTSKSTFGSLYKCYDEFKKGGYTIEFNRIRVFTYFQLLLIILNTILCVLTV
KREFRLSKTDTFKLYPMLFSAFWSMSCVCVWHLVYTVIAVTNNLLSVFGECIRDVIDSWQGVMYQPSNVSSISAL
QVVSTAVKRMREIQINIYRVSKGVNSAYGISVLVLGILNLFNITFFLLFNMGVFHKEGLLKYEIMVFSFLWTSAYLIPL
LLIVGGCDGVQNKAEKIGILVSEALLKIQNHEIRRELHLFSHQLHHAKIRFTACGFFPLDYSILTSMTAAVMTHLVVLV
QFQLSSKENSLCVCPSTNVTEVPASPTTTLS
>CsplGr43FIX
MPQNKNDCTSNDIDRSLGALLPLSRAMGLAPCRTHGRYQKSYTNSLYYYLYSFLFGITFLCHILMLTNDGVISSTS
SLNTMLPVIWTIAEVNIISITVLKVSRSKETLGCILEDLDKCCRILKKTGYRISCGKVKTVVNILMLLFLIVVITCLFNFR
KEFGKDNYHMNLIYSILFLVWFVNCVCVWILMNTIIFMTYVLLIVLGENIRDLSDSGLFFVMDVPVISTHKEHVTPRI
VKRWREIQINVYRVSKNLNGVFGLAIVGLVLLNFCNLTLILYFIIAEITVARDTSLEYELLVYGLPWTWGCFIHLILVVA
GCNGVHAMADRTGVLASELLLKIQNHEIRRELHLFSNQLLHAKIRFTACGFFQLDYSILTSMFAAVVTHLVILIQFQF
SGKNHSPGTCLTRNSTHKAEFTANSSL
>CsplGr44FIX
MRDNAHDGVLKNFGHLLMLNKAIGVAPFSQSGRMTNHYCIGLIYPILFGLTIIIVLLQLASKNFLGSPLRGTLMVVW
DSYHMTMCCVVSFHFSRSHQSLRHIFEDLNACKDILLRRGQQTEFSGVRANANFQILFLILLSIVCVLNLVLSYQVF
YPTNLGFTYILFSNIWSIICASQWGQMRTTVHMLFVLIKILNKHICNANASHSWLEKSSVYNAEYEEFILSFLENWK
KIESIIYRIFKNVNRVYGFTVLILGSINLINITLLLYYFIDRLIDSKQAVTFMGQEIFTFILLWTIFYLTPLLSIVEVCKRTQ
NESGRPVVFVSEALCKVNSKKARRELHLFSYQLLHTKIQFSACGFFPIDYSLLTSMAAAVVTHLVVLVQFQLSTKD
RSQCICPFTYGTEVPLLPPTPRS
>CsplGr45PSE
MGDRQLLWALKPMLWLNTTIGIAPPLLSSESEDKLLRRHRRRSIAVAFSMAFLTLLLTISSLIDIETFSLNSVISQMIT
VAWMLFYVTFGATSGCNYVVRLTAVTKIFKMLKNFEAILQSCPTAPLKTVRQSIECQLILVVFTTVQFFLNIWIVGYT
HGYDANFLITCVISIWAFINFMQNWQFCNKVLLLLKCFTSINIRIASLEIVDGFDGHRPHCDELGETVVSRIMYMKKL
QLNAYHVVEKMCGVYGISNFFFVALNFFNGTFELYYLIDSMVYVDRGAAWDSYFRTSTFLWVSAFFIQFYRTISAC
HRTQGETDQTGLVVFEALLKTMSNEVRTELHLLSLLHTKIRFTASGFFFLDRSLLTSISAAVLTYVVILVQFQISRKA
PCICT
>CsplGr46aJOI
MKLERSLVTLLFMFKISGMSLAYQLTPGKDSRNLERKLAVIYRATLGAAGVLLSFTSVTISCYHWQQMANEKLIPPF
WPTFEAFWMSSHFALGALTFAWFQLRCSQLSKLIQNLSRLGGEIDVRDKFLSRLKIWGLCLASGFSFFTALIVCVY
SIILSKRPWEKYNVITNTICSVVILTFQLYYLLVLYILAYKITVLNKNIRQFGCERESAAKDSTFRRDAKRVFQGSFLK
HTSHLKVISYLACFNLKLHSAFKITNEIFGLVILCQFAYSILNATFQLFDMLMNEGILHISLSSLFADSFVFFIYMFGFT
SIIALGQITEKQADRTSQLVTEAILKVKDDRLRRELRLFSHQLLFTRIRFTACGFFSLDFSLLTSMTAAVVTHLVILVQ
FQVADKQAACLC
>CsplGr46bPJ
MDVTRCEQIPPRSQSKRVDVKIYRPIAPLLLMFQACGMSLKDLLTSRRDETGFRRKLNSALVVFVAAVGVLFSSAS
MAVSFHEFRKVAAEGKIPVFWAVFEFIWLLIHHVLGZAVYAWFQLRRCQIARLMRILARTCCEIGLKEKFTAKLTILG
LCQLAYFSLFLICSVFAQYLTIMKLPRGATNAVAITLWTVVNQIIQLYLILVLCSFLCNIIILNNGIEKLGCNRGSIEMES
YVSHDANRKILFRGDGLENMGHFKEIMYWLSFHLELHSLFRSTNSIFGLVTLFQLSYSLLHTTFQLFDLINFILTDEP
ISTYFVSIYIILSFSLGSIAIIALCQDIKYKADRTSQLVTEAILKVKDDRLRRELRLFSHQLLFTRIRFTACGFFSLDFSLL
TSMTAAVVTHLVILVQFQVADKQAACLC

>CsplGr46cJOI
MVVISFPCKRAQRKFSLKARSSSNIFFSQNVIFPPRDGTMAGESFSLEKMDVFWVLSPIMRISKALGLAPFRLSVS
RKMRKSESKADCSVYYSSLKFSLFTIITFSMIISDFVMIEKYPGSWLVNKYLNIVWDSVDNLLSAGSVLVLLLRREK
SRDLLVRIAEYDRETDGSDYFLEERKVVEGQIAYVIVAFTIFGAYVYYGLHVLEVYGYLESVKVLIIVQWICSDLMM
HLQLYNVLLLLRRRLFRLNSRLKSLQYVRAGDWPEIVGGHPEISVGIIRRCRSRFYQIFQMCNRANHIYGITVSFSI
FYNFLDSTIILYFLLTMEVQDKVDIDNLHDLLYNGMLVVILSITLVLIISVCESILKEADRTSQLVTEAILKVKDDRLRRE
LRLFSHQLLFTRIRFTACGFFSLDFSLLTSMTAAVVTHLVILVQFQVADKQAACLC
>CsplGr47aJOI
MGKDVYYAISSLLLVSRIFGVAPFHTTYQRGRGKGSLPKWPLIASVSVITVTSLITVSALPSMESRRMKRFSNQFLT
VVSTTSTVLSGSAGIVSVYLFLLRSATAGKVLRGLRKYDDAYRVKGNADFRELRRKVRTTMFFVYLVYSLLSLNLIF
TFLKFSGHFNLFAQCILTYWSFIVVLLKTQFLSMLISLRQRISRLNEDVRLRLTSLPLPEYQHQWRGPRNTGGLPG
KARWWTMMQIRIFYLSKMTSEVYGPSWVPLMIYHFLNTTSVSYYSILYLFNESDFPNATGNPLAPGVWVCHQLA
GVFIVVVTCAGVSNEADQTGVLVSEALLKVKNHKARRELHLFSHQLLHTKIRFTACGFFPLDYSLLTSMTAAVVTH
LVILVQFQLSGKEKPTCHCPYFNTSEIPPPSTTAMY
>CsplGr47bJOI
MVLSEERERTKEDLILAMGPFLRFNRALGILFLDPENKRGENNFHKSQELSLLIHSLLMMGNFLLPVYMISSNDQF
NNTVGLNRAVNVFWIMLENYLNAISAFTLIPRLGKVRRIWMELFSFEEKVMADVKETQWSRASIRLRSFFAAYVYT
HFGVNWIVRVYVNGPLSLELLAFSHVIFWTTNLVMQGWQCYNAVALLHRCLFGLNENLRQQHQFRKGSGNSEIL
RELRNRVLVKKIRHLKALQVRVFHIFRSVSSVYAIPCLTLAALTLVNLTFTAYYALELAGITDVPGMPPFHFTCALAW
GVSYTTDLVLIASAGDGINKEADQTGVLVSEALLKVKNHKARRELHLFSHQLLHTKIRFTACGFFPLDYSLLTSMTA
AVVTHLVILVQFQLSGKEKPTCHCPYFNTSEIPPPSTTAMY
>CsplGr47cJOI
MTEGENFPEGVLWALKPVLAFNWALGISPFYSDRTLDPKDSRSHYLVAIAMYSILVMLEFFMLPYGIFLFSKFDPN
ALVSDAVMTMWQITMIILNTSAGYTFVLRYSASRETIIKLLDFEETLVKPSLISLRKTRLWICSMAFYVAFAIVQFGIH
MRETVGPYGIFSIPSFLNFRMMFWSFSNLMINLQCFCLVLLLSWCILNTNASIRQTWEYFSSRNPGAFSDQEKRF
LFGRIKHLRKLQMQTYRIFRSLTNVYGLSTFAIGVSVLFCVTFMLYCILDVFLSKEPVLSVAMLIISFLWVVAIVQIFILI
LSACEKTQLQADQTGVLVSEALLKVKNHKARRELHLFSHQLLHTKIRFTACGFFPLDYSLLTSMTAAVVTHLVILVQ
FQLSGKEKPTCHCPYFNTSEIPPPSTTAMY
>CsplGr47dFJ
MKNTLRTANVIWAFNHLLRVNQFIGVAPFCLDKGGSRLAGKSRHRLEYGVIVYAVVSFLSFSFSTHVTVRLDHYLT
NSATSQTVLILWTILGELIGAISGFTFSLKLHVVQRVFRHFARYEKLSFEQSGIKLQRVRRSVKGQLIYVACALAILSS
NIGHTLSKRGFGVPFCNYLLVAFWAFVNMIQGLQFYSMMILLQSNFVDINDKLSSNIPPSSAVPNEAFNSSRFHAE
EGSAHRFKICRKLRMRCYHTHRDLNYLYGVSSLAMGGLNLVNVTFNLYCLTDLFINGERATMMPLQLTLSSAWVM
VALAPLVLVIFVCEATLMEADQTGVLVSEALLKVKNHKARRELHLFSHQLLHTKIRFTACGFFPLDYSLLTSMTAAVV
THLVILVQFQLSGKEKPTCHCPYFNTSEIPPPSTTAMY
>CsplGr47e
MYRLGLINTHWDVYLILRYLIGLTRILGVIPCLKQRGGRKWAFYNSWHRLIQNVLSFLTILSLTRTALGKSAISSTTAL
SNSLMNFWCAIETFVVLLAAQHFSLSRQAVQCIIKDLSTCNKLLAPSASQVASSKLTCSVRIQILFLTMAIAQSLSSFI
TGYKSGWKRIDFISLFLMNIWFAVSACQWSHLYATVFLLHLYLCTLNGFLRNLRSSFPGEKGSDATTSCTIDLRKN
FVAGFTRRERAAQLRAFRVFKNTSKAHSIAIIALGTQNLINITLICYFAINGMMNKEEVIMSGGDMFSTGFWTLNNF
TQMFLVVKVCNRTQNEADQTGVLVSEALLKVKNHKARRELHLFSHQLLHTKIRFTACGFFPLDYSLLTSMTAAVVT
HLVILVQFQLSGKEKPTCHCPYFNTSEIPPPSTTAMY
>CsplGr47f
MLMLSNLIGLAPSLIHLNGGNQSFRHYLRKIIISNLILATLSGVLLIQTLMNNWLFQLNSQVSQVVMITWLSSYISLG
AISGCVFTTKLANVKDIFIRLNEFEQELSRCSMVTLREIRKSILIQRALVYFLCLELLANIWSMGNSYGFSTYVFTMS
AVVIMWSFVNVIQQWQFCINAYLILRCFSNLNNEIYSWGKSNVIATSSYGSGRTSSECDSEAGKLRILRKLQLNAY
KTFKKICIVYGTSTFLCATLNFFNATFILYYLLDLISYQSHAAWSASFLLTSSTWVTTYFILFYCVLSTCVRAEKMAD
QTGVLVSEALLKVKNHKARRELHLFSHQLLHTKIRFTACGFFPLDYSLLTSMTAAVVTHLVILVQFQLSGKEKPTCH
CPYFNTSEIPPPSTTAMY
>CsplGr47g
MAEKKFSREEEVHHWLPKPLFLLHRAFGIAPWTLHSSTQRDPIQICVKTIYTAAFLCSLALNSCAWVIEIKYVFHTF
VTSLVIGLRCYDAFFGSFSVYIFALRYYAIKNIFFGLISIINIERNSNSRKTSLLSRILKFIYVLIVLFFICITFEGIFKSKIP
HLSKLTTLAIKTSLIWRFLNVLQIWQFISKVDTVRNVINYWNSKIKNLKNGAKNNQTSGAEFNQDCREPSAKEIRR
MRRVHLKIFIVTKEIQSVYGISAFTFIVRNFVTITFSIYMPLIIYLKLNNSVQNNEVTHRFWILWAANFLASSLLVIYCE
EFFNEADQTGVLVSEALLKVKNHKARRELHLFSHQLLHTKIRFTACGFFPLDYSLLTSMTAAVVTHLVILVQFQLSG
KEKPTCHCPYFNTSEIPPPSTTAMY

>CsplGr48
MARTQYSREKELDHRFSEPGLIYRFFGIAPWAIHSNGQSDASLKYVKAFYTVALIFSLALAACESYAKSRKKSLFH
SLTVSIVYYDIVVGCINGYVFVLRHNAVKNIFVDLMSIGKILNIHRDKRNNSLRSYLAGLFRLLILLFFILLTLERLFIFTL
PHLNSTTTISIKLAFIWNTVTFLQEWQLLNKMTVIRNFINNLNLRTRNLANGSYDMRASGVPLHREHGIGRLMMEI
RRLRQINLKIFAVIKDIQCVYGVPTLVFITRSLMGITLQYYLLVSDYLEKIMGAKNNDRTRYFWISGTIQLLATWIIAAS
CESTLSKADQTGLLVSEALLKVESHKARRELHLFSHQLFHTKVNLTACGFFPLGHSLLKSMAASVLIYFVILIQFQL
SVK
>CsplGr49aFJ
MRGGSRSRNNPREDMLWSLQPILIHGRIIGLPPYSSNRDEKKESHRYWPYLCHACVILTTWTVVTTVCISMIVSGY
DHFTRDSSLSLYLMLTWTLLENGLRIAAVCSLVHKRHACQNFFANLIKYDDSLQDTKTLRHRCTRKTVVNIMVFFV
CLWSFPLLLAVVRSNDAINKLQAVLSSFNSAMILVSGLQFKAVLIVLHLRVSSLNKEIQNLYGNERSVVKIPRSLVKL
RTRRCIKGPIREIEARQLYLFRLCKMLNNIYQVSNLFHNIANLITFVFTLYYILVYLVIESFPQLVTEVTLYTTWKVTVT
LLSTAMSVNSCEEISMTADQTGVLVSEALLKVKDHQARRELRLFSHQLLHTKVRFTACGFFSLDFSLLTSMTAAVV
THLVILVQFQLAGRDTPTTCNCTQENSSMTMTGLVTTPLP
>CsplGr49bFPJ
MRGGSSSRKNPRENMLWZLQPILIHGRIIGLPPYSCINGEMKESHRELPYLFHASRIZTTWAVGTIVSSANTFSAN
DHYTRDSIIGLYVLLSWVLLVNGLAIVAVFSLVRLCHVYETFFADLIKYYDCLHDTKTLRNSYTRMTVVNXLIFVVCLL
CFPLFLEIVRSYDSISMLRAVLINSAMVLVSGLQLKAVLTVLQLPVSSLNLEIQNLYGYERSVVELPRSLVKLRTRRC
IIGAIRELDDRQLNLFRLCRRLNSIYQISNLFLNIANPMPFVFTLYYILVYPVIESYPRZINEIALPSTWQVAITFLSTAM
SINSCEEXMTADQTGVLVSEALLKVKDHQARRELRLFSHQLLHTKVRFTACGFFSLDFSLLTSMTAAVVTHLVILVQ
FQLAGRDTPTTCNCTQENSSMTMTGLVTTPLP
>CsplGr49cFJ
MRGRSSSRKNLREGMLWSLQPILTLGRIIGLPTYSCISGEMKESLRDWPYLFHAYGILTTWTVVTIVSIANFLSGND
HFIRGISLSQFVMLFWELLGNGLPIAAVFSLVHQRHACENFFADLIKYDDCLYYTKSLRHRCTRKTVVNIMVFVVCL
WPFPLFLAVVRSYNAQGMLHAALSSCRSAMALVSGLQFKAVLIVLHQRVSSLNQEIQNLYGYERTVVELQRPLIKL
KSRRCILGTIREIKARQLHLFSLCRKLNNIYQVSNLFFNMANLMTFVFTLYSILVYLVIESFPQYVIEITVYYTWQVTIT
LLSTAMSVNSCEETSTSADQTGVLVSEALLKVKDHQARRELRLFSHQLLHTKVRFTACGFFSLDFSLLTSMTAAVV
THLVILVQFQLAGRDTPTTCNCTQENSSMTMTGLVTTPLP
>CsplGr49dFJ
MRGRSSSRNNPREDLLWSLQPILIHGKIIGLPPYSNINGEMKKSHRDWPYLCHACGILITWTLVAIGSIANILLGNNH
FTRDSSLSLYVMVSWVLLENGLAIVAVCSLLHQRHACEMFFADLIKYDDCLQDNKTLRHSCTRKTVVNITAFIMCL
WCCPLILAVVLSYDALDMLKTALSVFNSAMVLVSGIQFKAILIVLHLRVSSLNQEIHNLYGYERTVVEHPRSLVKMR
SRICILDTIRELEARQLNLFRLCRELNNIYQVSNLFFNIANLMTLVFALYYLLVYLVIEPFPQYLTEVALYCTWQVTIILL
STAMSVYSCEETSMTADQTGVLVSEALLKVKDHQARRELRLFSHQLLHTKVRFTACGFFSLDFSLLTSMTAAVVT
HLVILVQFQLAGRDTPTTCNCTQENSSMTMTGLVTTPLP
>CsplGr49eFJ
MREENSSKNNPREDMLWSLKPILIHGRIIGLPPYSCFKDDNKESIRDWPYLCHASGILTTWTVGTIVSSANIFSGND
QYTRDSIISLYVLWSWVLLGNSLAILAVCSLFHQRHAYENFFADLIKYHDFLHDTKTVRHRCTRKIVVNITAFIVCLW
PLPLFLAVVHSAEALGILNAALASFNSAMVLVSGFQFKAVLIVLHLQVSSLNQEIQNLYGYERSVVELPEPLVKMRT
RRCIIDTIRELEARQLNLFRLCRKLNNIYQISNLFLSIGNLMTFVFALYYILVYLVIESFPQCVTEIALYSTWQVTVALLS
TAMSVNSCEETSMTADQTGVLVSEALLKVKDHQARRELRLFSHQLLHTKVRFTACGFFSLDFSLLTSMTAAVVTH
LVILVQFQLAGRDTPTTCNCTQENSSMTMTGLVTTPLP
>CsplGr49fFJ
MGGGSSSRNNPRENMLWSLQPILIHGRIIGLPLYSCIRDEKKESHRDWSYLCHASGILTAWTVVTIVSIALILSGYD
HFTRDSIISQIVAFTWALLGNGLRIAAVDSLVHQRHACQNFIADLIKYDDCLQDTKTLRHRCTRKNVVNIMVFVVCL
WCFPLFLEIVRSYDTLGMLKAVLASFNSVMVLVSGLQFKGILIVLHLRVSSLNQEIQNLYCYERSVVEPPRSLVKLR
TRRCIIDTIREIEACQLYLFRLCKMLNNIYQISNFISQYCKSDYFCFYALLYAGLPSDRIVSAIRNRKRTSLCLASHHHI
AKYGHECADQTGVLVSEALLKVKDHQARRELRLFSHQLLHTKVRFTACGFFSLDFSLLTSMTAAVVTHLVILVQFQ
LAGRDTPTTCNCTQENSSMTMTGLVTTPLP
>CsplGr49gPJ
IRGRRSSRNNSREDMLWSMKPILILZRIIGLPPYSYINGEMKKVHRDWPYLCLSYZILTTSKVGTIMSTANILSGND
QYTRDYIIYLYVLLSWFLLGNSFDIAAVCPLVHLRHACDNFFADMIKYDDCLHDTKTLGHRCTSKTVVNIMVYIVFM
WSCPLFLTIVRSYDAVGKLNSALSLCRSAMVLVSGLQFKAVLIVLQLRVSSLNQEIHNLYGYERSVVELPRSLVKLR
TRRCLIGTNGEIEHRQLYTVTRFCLCRKLNNIYFKFGRADQTGVLVSEALLKVKDHQARRELRLFSHQLLHTKVRF
TACGFFSLDFSLLTSMTAAVVTHLVILVQFQLAGRDTPTTCNCTQENSSMTMTGLVTTPLP
>CsplGr49hPJ

REGCNVQHEFRKGVSWTLHSIPTLGRLMGLPPYCFIGNERKKPIRDGRLTFQAVGILATCIVGTIYSLETKZRGTTA
QFTYTSLYVTFSWSLMGNVLVFMAICSLVHRRHSCEKFLRDLIKYEESQRDTSTLKHSHTRKTVVSMMILCLGAW
CFPLAIVTCFDDIVSLTALSMIEVAMYLCSNAMVLVPGLQFKAFLIVLPLRVSSLNQGTRILVGXQTSILEHPMPSSK
LKIGRYMRDAIREARQLNLHILGRTLNDIYQVANLVHADQTGVLVSEALLKVKDHQARRELRLFSHQLLHTKVRFT
ACGFFSLDFSLLTSMTAAVVTHLVILVQFQLAGRDTPTTCNCTQENSSMTMTGLVTTPLP
>CsplGr49iFP
MSLVFRAPKVETAPIVCGGSSGKNKHLKDQFWSMHPMVAFGRIIGLPTNPFDGDGSREPRRDWLFPYHAAGLLT
KWGVGTIVSLASIASGEDPFTQDSPTSLIVMLLLTLMGNGIDMGAVVSLVHHRRTSEKFFGDLVKYDDGLRDTNTF
KHSHDWKDVVTMMALIVSSWCFLLALSXFGVSHSIHAZDMLKAELYLCSSAMVVVSGLQFKAFLIVLRLRFSRLH
RKYVPWRETKAQRRHIXCRMHERMTRDIEERQLNVHRLCRALYDIYQVPNLFYSIVNLTNSDFAFYSZFVFLVGD
FLFWYGDVISLYPNGQATLSLFNVPLDVSGCKEISDEADQTGVLVSEALLKVKDHQARRELRLFSHQLLHTKVRFT
ACGFFSLDFSLLTSMTAAVVTHLVILVQFQLAGRDTPTTCNCTQENSSMTMTGLVTTPLP
>CsplGr49jFP
MRGMSSSRNNPRKDMLWSLQPILIHGRMIGLPPYSSNSGEVKEAHRDWPYLCLAYGILITWTVGTIVSTVNILSRN
NQYTRDSSLSLYVMLSWVLLGNGLAIVAVCSMVRQRHDCENFFADLIKCDNCLQDTKTLRHSCTRKTVVNMMVFI
VFLWSFPLFLAVVRSYDALGMLNAALSLCRSAMDLVSGLQFKAVLTVLHLRVSNLNQEIQNLYRYESSVVELPRSL
VKLRTRRCIIDPIRDIEARQLNLYHLCRKLNNIYQFPNLFFSIGNLMNLVFTLZNIVYFLSDSFLRIISPFTLYNVWNIT
VIVLITTLDLNSCEETSIVADQTGVLVSEALLKVKDHQARRELRLFSHQLLHTKVRFTACGFFSLDFSLLTSMTAAVV
THLVILVQFQLAGRDTPTTCNCTQENSSMTMTGLVTTPLP
>CsplGr49kFIX
MRRRSSSRKNPIEDLLWSLQPILILARIIGLPPYSSIRGEMKEAHRDWPYLCIAYGILTTWTVGTIVSIASILSGNAHYI
YYSSTSLCVIFFSKLLGNGLVMLAVFSLVRQRHACESFFADLIKYDDCLHYTKTLRHRCTRKTVLNITAFILGMWSF
PLFLAVVHSTDVQEIVRAALFLCRSAMVLVFGLQFKAVLIVLHLRVSSLNQEIRNLYGYESSVVELPRSLVKLRTRR
CIMDTIREIKARQLNLYHLCRKLNNIYQVPNLLFSIANLMTLVFTFYSLIVYFLSDSFLRIISPFTLYNVWNITVIVLITTL
DLNSCEETSIVADQTGVLVSEALLKVKDHQARRELRLFSHQLLHTKVRFTACGFFSLDFSLLTSMTAAVVTHLVILV
QFQLAGRDTPTTCNCTQENSSMTMTGLVTTPLP
>CsplGr49lFP
MLLSPNNFSPNTLSTHYHIPLKCEGSNVQHDPRKGTWTLHSIPTFGKLMGLPPYCFIGDERKKPIRDGRFIFQAVE
MLATWIVLHDIFPRRNSNTVFAKVTYTSRYVIFSWSLLGNGLIIMAVSSLVHRQHSVEKFLRDLIKYDDNZQDTSTL
KHSHTRKTFVSMMILDLGAWCFPLAIATCFHVLVSLTALSLIILAISLCSNAMVLVHGLQFKAFLIVLHLRFSSLNYGI
RTFVGFETSVLEHPIPSSKSRIGRCMRDAIRDMEARQLNLHILGRTLNDLYQVANLCZNILHLVNIMFEFYFLYVCIG
GEPPMMHGYTFSVYSSWHIFPAILLMVTDMSSCVDISKXADQTGVLVSEALLKVKDHQARRELRLFSHQLLHTKV
RFTACGFFSLDFSLLTSMTAAVVTHLVILVQFQLAGRDTPTTCNCTQENSSMTMTGLVTTPLP
>CsplGr49m
MELSHHRSEKNTASETSVTFNRQCTDNKSAKDVFWSLGPLLKLGKIMGLPPYTSERDGKSQPHSEGYFMYQTLI
VLMAWIAGVLVSIANMISGNDEFTRDSSTTLYIMVFSMLVVNILVMTAVCSMVLHGEACEDFLRDLFKYDGGLRDIK
TLKYSSTRRTIVSMMASFAFGWCFPLVLTAYFILVYSVDRGIILKGALTSGRSVMIIVPGLQFRALLIVLHQRMSCLN
QEIQSLVNFNSERPTRSNQVRNGRCLRDTICDAKARQLNLYRLCKVLNDIYQVPNLFYNIVNLTQTIFVLYFLFIYIK
GDLSVGFPNAATFYMTCVFMLNLAVTVLDMSSCAALSKEADQTGVLVSEALLKVKDHQARRELRLFSHQLLHTK
VRFTACGFFSLDFSLLTSMTAAVVTHLVILVQFQLAGRDTPTTCNCTQENSSMTMTGLVTTPLP
>CsplGr49n
MGTQDVYWAIEPMAKTCGYLSLLPLSTFRDRIHPHDWKERKFCVVTVGLSLFSAIFTLVMINMKNGFGMVNDGLS
ASANYAWIVIENSMGIICTLSLLLGRYSFRRIVEECVAFDKVYRGDISLELVKSRAYVRKQVAHSMFSAALLLIILLIRS
QEGIRRTDVVMAFCAVFSLLHGQLAQGIFQTALHVTKIRTTRLNHSIRRSNHRISLSVKYHKGLLLKVHEWKSLEIIL
YRLRCSINRVFGIPCLMWVLYNMLSVIFLLYFWITKLLDFSTYQFGYMRTTTGTLWIGNFLAFTVVSTMICESTVKQ
ADQTGVLVSEALLKVKDHQARRELRLFSHQLLHTKVRFTACGFFSLDFSLLTSMTAAVVTHLVILVQFQLAGRDTP
TTCNCTQENSSMTMTGLVTTPLP
>CsplGr49oFIX
MNPGGKWRYPHTKQIKKHDGSHCKSCSPPCQERKDTRDVYWAIEPITKTCGYLGLLPLSILHDESHLHDWREW
KYYALALVLSSSSAIFPLVVITLVFFGVGHLTSDAAGYAWIVLENMLGFICILTLLRGRYSVKRILRECFTFDLEYKGD
ASLEHSQTRTCTQKLVIYGIFTATIPLVYIPFRYSLDESIEETLFTTGATISLTQSHFTQVIFQTVLYATKCRMTRLNENI
RRSIQRTTFSVKTLNRKEMVLKIEEWKWLEIILYRMQCCINYVFGIPCVFWAFYNLMNVTFFLYYFTITMTGYDNYY
HGYLRITLDLLWIGNDLVLMAISVKICDSAAKEADQTGVLVSEALLKVKDHQARRELRLFSHQLLHTKVRFTACGFF
SLDFSLLTSMTAAVVTHLVILVQFQLAGRDTPTTCNCTQENSSMTMTGLVTTPLP
>CsplGr49pFIX
MNTGGKWRYPHVKQIKKHVGRQFKSSRPPCLAKKDTRDVYWAIEPITKTCGYLGLLPLSILHDESHPHDWREWK

YCAVAVVLTASSAMFPLMINTLKFFGVGHNTLATVALCAWIMIENMLGFICILSLLFWRYSLKRILEECVSFDLAYKG
DASLELTRTRSYARKRVFCGMFSASILFTYILISYHSETSLQDTILIYGSILSVMYSHFTQMIFQIAAHVITARVIRLND
SIRRSSRGVSLSVKYSDHKGFRLKIQEWKSLEINLYRSRYCMNRVFGIPFLFWTFYNAFSVTCFLYFTVTTILDFGS
YKFGYLKSTFEILWNGISLVFMALSMMTCNSATKEADQTGVLVSEALLKVKDHQARRELRLFSHQLLHTKVRFTAC
GFFSLDFSLLTSMTAAVVTHLVILVQFQLAGRDTPTTCNCTQENSSMTMTGLVTTPLP
>CsplGr49q
MAATFRLGSKKVGAVADIKSQPSASGGNEGIAWATRPFVRLCVALGLVPFRTDNLEGRTQADGGGTSKLHLSYSV
AVFAAVAALFCAGAQRRIADREKSGEPAIQSAVQLVWSLLSGLVAVVSFGVLIAKRSELQEAMRDIAVIGTRIIPEET
VLFGKVRTLLIAEVSTVLALAVASVGLEYYSISSGLTHPREYEIIFHLFAFVATLLLSLQFINQLLLLQQLLAMVNERIT
GIRDSFTFILASRDKIGREKRSPLDAMESKDPVAVCVRRYAGFHFSLCHLGPRLGNVYGMLALLLSMFDLLDVAFQ
FYMLINSAINGTSQQEIIRRLGEIFLWSCPKVVTLVFLVAACQSVIEEADQTGVLVSEALLKVKDHQARRELRLFSH
QLLHTKVRFTACGFFSLDFSLLTSMTAAVVTHLVILVQFQLAGRDTPTTCNCTQENSSMTMTGLVTTPLP
>CsplGr49r
MMPAVNHRSAKTSAQDAAVRYSKPRRRQRRRMNFSRRRAKRSPCDVFWATWPLFLTSQVLGVAPFPLKYGRG
KVGTTRVLLNYSRFAFLFALLSSIYSLPEMVQYQINESAQMEIPLNAYVNASWWVMCILVGTASSLCFYSRRERVQ
QFLLDMVRFDAISKLKKSFHYRKTRRLLILNFFGLCSLAILALLHQVYSALTEALQDSNLLTLLGLYWLLVVLVLDSQ
FSTLTWMIKDRFRMVNEGVSHILHSGSSDSLGPLIVSAIGIGEDVSGLSTKFYPRVNGKNALSSYIRVLATLNMSLF
HLVRTLCQAYGPLVLVQTCSNLVQITFHLYYYIEEMMGLLESDDPVYIVGDLAVWCCPYIAGISMMLASSHKTAKEA
DQTGVLVSEALLKVKDHQARRELRLFSHQLLHTKVRFTACGFFSLDFSLLTSMTAAVVTHLVILVQFQLAGRDTPT
TCNCTQENSSMTMTGLVTTPLP
>CsplGr50aJOI
MLLRHVIDRDIFFALMPFLRICKLAGMTPFPLHNYRGHKPPGKCSVYISWSIFSFLAFVTTIMVPWLMTRGAMHST
GTTGLKMIVLKVWVASRDVVGISTTAMFLLKYNKVDRIFQVMADFDSNVSRTSFLKLKETRRHCIKLVVFLWISLSL
FCFFVTYAILIHYSAEESANVTINLCWTGFIVLLQMQFYIILYSIRIRISCLSEAVYSSIGETSDPHFFDRRVNYLKQYA
PYKSVKSPVSWMGTLANQQVKVYRICVDAHEVYGIHSLMLALVSYLDAISLLFFVILRLHHEGSFSDFAFFLSSLW
TVYAVFLLLLTIIACESVHKEADKTGTLVSEALLKVQDHQTRRELRLFSHQLLHTKVRFTACGFFTLDFSLLTSMTAA
VVTHLVILVQFQLSGKDADCCCALYNSTEVVESLTTPNP
>CsplGr50bJP
MKSTVNGFGENIFHALAPLLKVSRALVIPPCGTRLLPNGNRTFNKGALALSILVLSVLSLITPVSCPEVVNVKTDFR
AGCHKLTIVLWCTWLLTNAAVGIATVALALKNNRKIDSVYQALAEYDFKTSRKLSAENNGSVQRRVQLQLIFIFTYA
AILVANTFRTCVFNNNEPFRAVNELLMVTWNLHKLILSLQFGHLISLIKMRFRRZNDSILLFSTPPKADIVWELRETC
TEQSCRLFRVGNSLKIVNRWSDLQMKLYCSSCEVKTVCGMHFLFFALQDLLDATFLSLFELSFEHCASLFVSTLL
WIFHNTCFLVWVISECVAVHEEADKTGTLVSEALLKVQDHQTRRELRLFSHQLLHTKVRFTACGFFTLDFSLLTSM
TAAVVTHLVILVQFQLSGKDADCCCALYNSTEVVESLTTPNP
>CsplGr50cFJ
MTSYGQGAHEDILAVMAPLLKICRIIGAPPYTLHYIGEGKCASKSSHLLFGIVFWALTTFTSVIVYAIRINVGGGLRN
STRLLNTAIYIWLLVHQLFGMASSAFALNYHFRIDILFREIGACYSRIGNKTLKRLGLVEVKTKVRWQTVFIIAFNVSS
IMDRLIKYLITYTFYDKAANALLMIFWNFYNVIFLLQLGDFFILMKHSFQYLNEQIIISAENNIKNKCKGKKIESLFSEY
CLFTNSKVAELMYLQMKFYKCFRMINSAYGFQPLLFALLIFIDATVVIFFDIGTMSSIYDLHWSPFWLTYKLSILIWLL
LEGSRLCREADKTGTLVSEALLKVQDHQTRRELRLFSHQLLHTKVRFTACGFFTLDFSLLTSMTAAVVTHLVILVQF
QLSGKDADCCCALYNSTEVVESLTTPNP
>CsplGr50dJP
MSPRVKLSGEVNIFTETAPFLRFCRVLGVDPYPLHFHVDLKAPSETQVVRNSMALWGILSILTVVCCHSGMSTFVT
YHDNPSKFRNYVIYSWVVICEVIGITMVGFCIFNHKMIGEILQNIARRDSETKSYHPHMFVQVRKVVTVKLWYLSM
TSAPFVVKAIYDAYLRDNVGKFLIFLSTLIWDQYSQSIAIQFCHFLLWIRIRLRCLSDDIRSLHLSSKDTIDNKCDRGL
IGVHLTKDSNSQKLVAYRSNLQGQLLRRCSSINIAYGVPCLLLVLLNFFNVTYTLFLKRMTKIADZILNISMHLYWVM
SKLGILTWLILLZDFLSKEADKTGTLVSEALLKVQDHQTRRELRLFSHQLLHTKVRFTACGFFTLDFSLLTSMTAAV
VTHLVILVQFQLSGKDADCCCALYNSTEVVESLTTPNP
>CsplGr50e
MALWRNGVAEDIFTVMAPFLKICRVLCIPPFTQHHCGVGNYVPNRKGVRLAVAVWTVLIFSSVIIFLIRLNRERGLG
SNNSRNFVQTTMYAWIMVIQCFGMVSVGFALKSQLLIDVLFQEFAECHLKIGRKRASLGLEEIRKGVRWHVVLVFV
GNACFVMDVLYEYLIKDISYENVANGILCIIWNMCTLIFTLQLGFFFSAMRHRYHYLNKKIMSFHESTSEKTSLRKK
CETIAFILRPFPNKKPAECLSQWSEMQIEFYHLFRVVNLAYGLQPFSLVLAAFFDATVMTFFQSSLRSSFFDSMCT
LFWIFYKLSILVWLIHEGDGFYKEADKTGTLVSEALLKVQDHQTRRELRLFSHQLLHTKVRFTACGFFTLDFSLLTS
MTAAVVTHLVILVQFQLSGKDADCCCALYNSTEVVESLTTPNP
>CsplGr50f

MKPSETLSDGEDIFAVMEPLMKICRILRVPPYAQCYFGKRNYAQNRRVVHFGIAYVTILLFSSVISDFIRVYLEVGFL
KSSSHLFTTTRYAWIVVHQVFGTTGVAFGLKHHLLIDVIFRELTESCIKMRGNTTSFGLREVKKNVRWQVTHIIVYN
AIFLMMLVDEHIITKSKYERAANALLNIIWNFYNVVFILQLGNIFILMRYSYRLINDKIISIDKSIFLKNRRWRKKENVLG
YSTSYNVSPVDMTRQLCKLEIKLYNIFHMVNSAYGVQPLLLALTAFFDAIFMTFFDIVEISSINDLISFLFWISYNLSIFI
WLLLTGNSFYNEADKTGTLVSEALLKVQDHQTRRELRLFSHQLLHTKVRFTACGFFTLDFSLLTSMTAAVVTHLVIL
VQFQLSGKDADCCCALYNSTEVVESLTTPNP
>CsplGr50g
MPLIERDGGEYIFHAMSPLLKVSRAFAIPPYTVKFLRNGNRVTNKRAQAVGILVWGILSFATLAIFPEIFQAKAEFRA
GYQGLRIMAWYASTLTHEASGIVLVALALKDRRRIEDVYQALAEYDVMTRRKTSIGNDDGIRRRVQLQLAFLFSCV
VCFQMNTFRNWALNNSDLFRVANESLVLMWNFYYLILNLQFGNMIGLIKSGFRRINVEIPLLYTMTKTCVIWKSRT
TDQSYRQILDLNPLKKVILWSDHQMELYYISRKVNTVCGIHFLFFSLVNFLEATLSAFFESSLSDAAVLSWSLIWVL
HNLCVLFWLLAECMAVQKEADKTGTLVSEALLKVQDHQTRRELRLFSHQLLHTKVRFTACGFFTLDFSLLTSMTA
AVVTHLVILVQFQLSGKDADCCCALYNSTEVVESLTTPNP
>CsplGr50h
MISSGPIITEDIFAVMSPLLKVCRVLRVPPYTLHYHSERKYAYNKKNVLMGTVLWTAVTLISAIFYEIRFNMEIGFRTS
SRGLVTTTVYVWILVHQIIGMTASAFAFKDHLRIDIIFQKLGGRHLKNGSMMSFLDLGEVRNKVRLQVALVILCNIYFI
ANRLYEHLIKGGHYDKAANAMMTIIWNFYTILFVLQLGNIFIFMKLRFRYLNGEIKSLGANKNDRLSSCRKNTVVGL
ASDTPENVTHVIFVKKLSILHIKFYHICRIVNHSYGIQTVLLYLTVFFDATFMPFFDTWTLTSLYDLHWFLSWTSYPL
GIFIWLILEGDKLCKEADKTGTLVSEALLKVQDHQTRRELRLFSHQLLHTKVRFTACGFFTLDFSLLTSMTAAVVTH
LVILVQFQLSGKDADCCCALYNSTEVVESLTTPNP
>CsplGr50i
MVSMERGCDDIFTAMAPLLKISRVLSLPPYTQHYIRKGNYAPNKGRVHVGTAIMMVLHLTSVISYLIMLKIDFELLNE
GQAFVQAAKYAWIIVHQVFGFASVAYSLKFHIIIDVLYQKINECYLQLGGRKTSFVLLEVRKKARLQLAFLITCNTLFL
MDCINEHIIKKTELVRVANELPSMIWNVYNVVFTMHLGNVLILMRYCYQYINDKITILGKTRHEETSRRKINNSNSLS
ISFNPSVKQLKLVWHTSEIQIQLYHIFREVNIAYGIQPLLLTLITIFATTLMIFYDGWSDSTFYDLAWTFSSIFFTLSVLV
WLLLECDHFCGEADKTGTLVSEALLKVQDHQTRRELRLFSHQLLHTKVRFTACGFFTLDFSLLTSMTAAVVTHLVI
LVQFQLSGKDADCCCALYNSTEVVESLTTPNP
>CsplGr50j
MNVAFRIILIKLLFYCPSAERTLFLPYKMTSIQGIRLGESIFTAMAPFWKLCRFFGAAPYPLYFLGQKKAPSHFLFLR
YSWTLWVVLLLLTIGWFRRVLYMRDEIFSYTSTLTNIGLFAWLGASDCVGATGMAFYLFHHSRFNAVLQSIAFQDY
NIDTMYSLNTVGARRVVTIELSFFLLSTLIFLIREIYEGYVVKSFDRVVISLFTIMWYQYCQIISMQFCNLLIWIRRRFG
CLNDEIRYRGNFIQVKTEVELQLFQNHPALYDEFLIRINYWSNLYSELFCICRNMSPIYGIQSLLHVLLNIINATITIFVL
TSQDDKGGNAFLFSFFWIFCKFGTLVWLIVECESLYAEADKTGTLVSEALLKVQDHQTRRELRLFSHQLLHTKVRF
TACGFFTLDFSLLTSMTAAVVTHLVILVQFQLSGKDADCCCALYNSTEVVESLTTPNP
>CsplGr50k
MESRGMERGEDIFRAMAPFLRVSRTLGIPPYAVRFLQDGIQVSEKRVLVRSFLFWGILSLVSIAVYPEIFLVESDFG
VSYRDLVLITWYAWTFINHLTGSAELALALHNHRRIDAVYQDFAEYDVMTRRKMSINYTNAIRKRVWFELSSVFVC
AMSFLMSTLSKLSFTKSYIFESANAVLSVIWNIYKQILGIHFGNLLFLINIHLRGLNDQILLLLTGKGARFKKFDKICRF
NPPTTDVNSWKIVKRWSDLQIKLYCYSRAVNTLWGIHILFFSLLNLVDATTLMLFQLTMYYGRRRFMRALFWVLQN
MSILVWILAECEAVSKEADKTGTLVSEALLKVQDHQTRRELRLFSHQLLHTKVRFTACGFFTLDFSLLTSMTAAVVT
HLVILVQFQLSGKDADCCCALYNSTEVVESLTTPNP
>CsplGr50l
MHQDEDMFFAIAPLMKPCQLVGLAPITLRKCLQKDNPNSKRFPWISLSSTIIAFTATILCLPFTVTEEVEEEFLILRM
WFIGEDFLGMFSMASFFRNLSKMETLFQGIAMYAEGIAEKADLKLRQTRRRILFHTLLIGAFFTYNAWGIISSVLSR
HRAGNYVNSAFNLLWMMVVVILNFQLYGVLILIKTCAFSLNGKILTLKEASESVRYRECPFVKTVLPSPKFNCASVS
NIRYLAEKHLSLRRLCYETTRVYGTHGFFLTLILFLDSTFLSFYATLRFQFGSSTDYLNLPISLTWVAFLLYLFTGVIN
SCEDICHEADKTGTLVSEALLKVQDHQTRRELRLFSHQLLHTKVRFTACGFFTLDFSLLTSMTAAVVTHLVILVQFQ
LSGKDADCCCALYNSTEVVESLTTPNP
>CsplGr51aJOI
MALALAGSRAPRSTDLSIRHHKVPQCDTTSVQSPGGEEKDVFWSLSMLLRLSRAVGCAAYPLSARDRGHKSRC
NLALPYSVGVFAIVTAFMSYTAMKVSSNQFVGKYRMSEAVEDSWVVFSYILVILRLALCLRNRRVIYAMLLKIRRMD
EKLNASGLVENDAGHEVLRRTTLKQLAYIVLYGSLSLIQVVLIDGDSYHVLAAFFSATWSTTITMQHLSLLLQFRRIL
NQLISDVDSFCVSPMTLRGSRKYFPALRRKKPTRSPRTHRRTLNSLSVRFRAWRSTFLSLHHWSVKMNNVYGKL
ILFHALINFLNLTLTFYFILSFIMDYGDLFFNLYEMSISIFWSFIDVVGVCAVAGIGYYATKEADSTGVLVSEVLLKVKD
HQARRELRLFSHQLLHTKIRFTACGFFTLDFSLLTSMTAAVVTHLVILVQFQLAGKDPSSSCSCNGTNTTLSPLDST
TPWANLTGE

>CsplGr51bJOI
MSPQLKTIRKPFIGQSQRNSVGCILRSNAPYLWLGIIFGLAPVPVGNRRGGRTPRWMLLHSAIIQLVGFTLTLYQNL
VAVERLRTDLSSAVSMIWMACAYSIIAFNSMLLIIRRGKYYEVFLSFVRLDERLKVTGNRVKNDEIRQGVRRQVCV
FCSCIGFLILLINPAINKAMRKAGYSYYFSMLYITISCFLLQLLQNNLLNNMCHRLDNLNSDIKQFCGSEYWRFGNN
ATNLKGYVRIWGSIQIDLFKLNSTIGNVFGVTMLLQIMLCLMNLTLYSYFTIIKLVIEKNFKETIALLCFGYIQMLLLIGE
VLVIRTCHTSKDKADSTGVLVSEVLLKVKDHQARRELRLFSHQLLHTKIRFTACGFFTLDFSLLTSMTAAVVTHLVIL
VQFQLAGKDPSSSCSCNGTNTTLSPLDSTTPWANLTGE
>CsplGr51cJOI
MVHQQQEIQNCSLSRSSDCTGGIFRTSGPFLWLGIIIGLTPIPVDHRRSGEYRTPRWMLLHSMIIHALGIMLILAQN
LDGLKSLRTDLPSAIGMLWLASSHSMVAFISLLLIIRRRKYYEILLSFARLDERLKVMGNKVEYDEIRLQVRRQMVV
FCSSIGFLIVIFHPLVNEAVRHSGYGLYATVIYITISCFSVQLLQNNLINNMCYRLDYFNSEVKNICIYEYWRFENNPM
SLKGYIRTLESIQIDLFKLSSTIGNVFGLIVLLQIVLSFVNVTVSSYFIIAYLEIIGNMGEVITLICTEYVNKLLLTGMILIIR
NCHSSKYKADSTGVLVSEVLLKVKDHQARRELRLFSHQLLHTKIRFTACGFFTLDFSLLTSMTAAVVTHLVILVQFQ
LAGKDPSSSCSCNGTNTTLSPLDSTTPWANLTGE
>CsplGr51dJOI
MATKLSSPVAPCAPVRPLDRRGERASVGRTSLTQERRREMHRAPKSRANVLARHSSRKETNGEERDIFWSLSP
LLFLSCIIGYGTDPRRPHDGTRGGILFDWPVLHSVVVLILFGALTADAVTIMCTSEYTGKGRLSEAVAHAWVALSYM
LLYIRFLCHLGHRRSLRKILLEVKGLDESMKLSLGRFTISADLRKLAIKQVSYIMSSFFYSIFVLDKIGSGPTMMIFSA
FLTLTTTIINMEHHNFLHQFGARLSLLNSDVKRFSASISGIPRASIIVSIESRRKAASVLKKDLSRFEFPLHHLRNWQ
SIYIALYQCIRSMNDLYGKLFVYQCILTFENATMVLYFTFLFLLNYNSRASSRDLHSILKPLGWMLFDMVQVVAIARS
GYCTTKKADSTGVLVSEVLLKVKDHQARRELRLFSHQLLHTKIRFTACGFFTLDFSLLTSMTAAVVTHLVILVQFQL
AGKDPSSSCSCNGTNTTLSPLDSTTPWANLTGE
>CsplGr51eFJ
MNSLKGRAKESRTSQYLPEAKVEKRDVFWSLSPLLYFGWVFGQASSHGMEGARGFDWPTLYSVFVVIATGTATA
VAASNLVYRLTERGLLSDAVAHIWASLSYLVMYARFLSFIGNRRRINEMLVELKKLDELINASLGCCVASVEMRNFAI
KQVTFALLFSIYSITLQDFTGSKLSSSACAAVLIGSNTVTNMKQLNLLRQFRARLFLLNSDVKIFGTPRTYKAGVPR
SPPVNLNGLRTYDDLSKASINPLVFSAQRSRAWREIQSSLYHCIHPMNKVFGGLFVYHMIFSLVNTTMVFFFNIIFL
LNYKSTVRGFSDTSNTMVWVLLDMSQIFAIAWSGYFATKEADSTGVLVSEVLLKVKDHQARRELRLFSHQLLHTKI
RFTACGFFTLDFSLLTSMTAAVVTHLVILVQFQLAGKDPSSSCSCNGTNTTLSPLDSTTPWANLTGE
>CsplGr51fJOI
MTSQTEGIPKQLSGKTRKCIGSIFWTSAPLIWLGVVFGVAPIPVGGRKGGGNRSPGWVILYSVAFQSVGICMMFL
QFFSIVGELKADLQSAVIMMWLACSYSMIAWNCTLLIIRRSQYYELFLRFSRLDKRLKDMGSEVDYGAIRQEVFRQ
VLAVSASVVPLFVLIFPAVNPFVRIEGYCSYLSGTHIIVSCSMIQLHQNNLLNNMCHRFNSLNSEIKRFYCRGNGGG
EGTKFWVYRSYDNDAMDKKKIDSVKIWKATHLDLFKLSSDINHFFGNSNILQGMFCLINATIFLYFCAVISEAHMNT
KEVYAYYYLGYFHFIPVAEWIVVIGNYHAAKDKADSTGVLVSEVLLKVKDHQARRELRLFSHQLLHTKIRFTACGFF
TLDFSLLTSMTAAVVTHLVILVQFQLAGKDPSSSCSCNGTNTTLSPLDSTTPWANLTGE
>CsplGr51gFJ
MTSNPMGIHYPSFGMSRKRNGNIFWTSAPLIWLGIVLGVAPIPVDHRKGGEYRTPRRVLLYSVAIQVLAIILMSVNV
IDSLTALSMDLPSAIGALWAVCSYYTTVRNSTSFIMRRCEYYEIFLRFTRLDKRVRAMNVRLKYAVTRKEVLRRVFA
FFAGIGVLTVIINPAFTEVMQVSGYTLYFSSLYETISSLLIQLLQNNLLNNLCHRFSCLNSEIKRLGYFETEGVENKTE
RVPKYESYGKNFGHKKNMPIAHEFLGDIKTWVKIQLDLFKLSSIINNFFGEAILLQAIFLPINATFYLYFWIVFSAVEN
DKKYLYAFIHYGCKPFIMLAGWVYTILACYTAKEKADSTGVLVSEVLLKVKDHQARRELRLFSHQLLHTKIRFTACG
FFTLDFSLLTSMTAAVVTHLVILVQFQLAGKDPSSSCSCNGTNTTLSPLDSTTPWANLTGE
>CsplGr51h
MSKQLSERETPKFFWAHAPLLKLSTALGVAPYPLRHDFTGQNEAPLWLLVYTSALLVATTSLKVFHVISMHIRWWP
IELGNALILIWTTFSYSVITWSVAAMLVGRKDVYGIFIRIACLEDKLESMTMDLDHPRLRRVLRRHVYVVMAYWAFL
AAIFNPELVPRMRRGGYVTYVATLHSSVIGSLTQIQLNALLINVRHRYSDLNEGIRNLSNREAGRKKRGRLTDLSG
RKPISSNYFCKGLRAWKLVHLELWRIISKINDVFGVFMLFHGMYCIMNGVIVFYFIMVDITSLHISPGEPLQLFSYST
LLMFEVYGVFLIIRSCRLLKFKADSTGVLVSEVLLKVKDHQARRELRLFSHQLLHTKIRFTACGFFTLDFSLLTSMTA
AVVTHLVILVQFQLAGKDPSSSCSCNGTNTTLSPLDSTTPWANLTGE
>CsplGr51iPSE
MSTEMDELSHAARVNDSRDPPGGGRDIFWTSAPLFRLSYLIGVAPIPTSYTRNRDNNTPRWVLVHTSTMIIAGMA
LMSAEVALLFLLIIPHRLYEAILVAWVACSLSLTSRNVVVLTVRRRKLCRHFARFIGLDERLQQMETKVDYLKIRREV
RRQVCVIVVVLVFLIVLLNPWMNRLMASPGNLFYFTTIQSAISSSLMQMLKNNLLMNNIXHRYRCFNSDIKDFKGF
ETGKCRMRSSVGGIACDPPIVPTAPKESEKLNKIRFWASMQLELFKTSIKLNNFFGSLIVFHVINCVMKMTADSTG
VLVSEVLLKVKDHQARRELRLFSHQLLHTKIRFTACGFFTLDFSLLTSMTAAVVTHLVILVQFQLAGKDPSSSCSCN

GTNTTLSPLDSTTPWANLTGE
>CsplGr51jFI
MPTTLQSRTRLRDAKLSMPLATNYGNLKDIYWAHAPVLYLSRMLGVAPFPLSKQSPESERTRGLFQTLWTMSFSI
NLSVFVSYAIVRLGWNNVTGLFSDAVDSDLMKYWNLLLYSLQARNIFALIIRRSSVSNVYQMFILVDEDMSSLGTF
VDFAEVRKSTTKLSSVLFLSAIIFTLFSYTEYAKAMFEGMQLVYLASLLTSTATFLLQAIQMSLLKDLSQRFALMNAG
IRATAKSTLRGSGAVKRYNGGLHKALDELSVEILSSQFDEGRRFHRWASIQMELFALSNYMNRLADSTGVLVSEV
LLKVKDHQARRELRLFSHQLLHTKIRFTACGFFTLDFSLLTSMTAAVVTHLVILVQFQLAGKDPSSSCSCNGTNTTL
SPLDSTTPWANLTGE
>CsplGr51kFIX
MTATLNSREHVHETKLSMPLAANDGRQKDIYWAQAPVLYMSRIFGVAPFPLSKQSPESERKGGIFRTLWIMSSSIS
LSVSVSYVLVHFLRTNITTISFDMVDLMKYWNILLYSLQARNIFALVVRRSNVSNVYRMFNLVDEDMSSLGTGVDF
AEVRKSTAKLASVLFLSAIILALFSNTEYTTAMFEGIQLVYLASLHISTATFLLQAIQMSLLKDLSQRYALMNAGIRATA
KSTLRGSSAVKTYHGGLHKALDELSVEILSSQFDEGRRFHRWASIQMELFALSNYMNRLFGSFILFDVVMFLIDMT
MSLYIFIVEMVFMVSVDTSRLVRVFLSSFWALFESGGILFVVANCHAVSNEADSTGVLVSEVLLKVKDHQARRELR
LFSHQLLHTKIRFTACGFFTLDFSLLTSMTAAVVTHLVILVQFQLAGKDPSSSCSCNGTNTTLSPLDSTTPWANLTG
E
>CsplGr51l
MTRPPKARKEREAERGDRGSEIFRGSAPLLRLCAVFGLAPFPLTYRGRKSSHDPPWARRYALLFMSVIAAAMVH
VTRKRYIRLKSWKLHQTVVLAGRTLIFALDFGIRVMIYFRRKQIYGIFIRFVHLYEKLGALEAAKLDNSKMRKIVIKHV
CMASASIVVLSIVFNPLFSELITLKGYCLYFLSIEVAVTACLELLLNNNIMANLYMSFRVLNLNIKQSMALLDNGIKGN
VSNDDDGNSICEVTIPSKSILKRLFQWKSVHLDLHKLSAAINSCYGPTILLYGTMGLSNTTITLYAIFNTFMGKVLPG
TSINYSSIFFIISRLYETVAFFLIMRTCHSTKKEADSTGVLVSEVLLKVKDHQARRELRLFSHQLLHTKIRFTACGFFT
LDFSLLTSMTAAVVTHLVILVQFQLAGKDPSSSCSCNGTNTTLSPLDSTTPWANLTGE
>CsplGr51m
MTTELKAEKKSEEEHGGQSVDIFWELSPLLRLCAVFGLAPFPLTSRDRKASHDSPWASRYTLLVMWVTAAGMVH
VTRYRLTCLVNWDLHRVLVTTWLTSIYAFNFCIRVTIYSHRKHIYGIFLRFAHLHEELHLLDAAKLDHSKMRKTVVKQ
VCMVLASFLLLSVVFRPSYVALVRKAGNCYYLLLLEVAMTSCLALLQQNIIMMSLCMNYSILNLNIKETISLFVYRRK
ATSSLYKEDDSARSNKIILQRLINWKSIHMDLFKLSVAINDTCGSTILVNGILVILTVIMMLYIALASFTSVMPGVNIDYP
AIFYLMLHLFETFGFFLVIRTCNSIRKEADSTGVLVSEVLLKVKDHQARRELRLFSHQLLHTKIRFTACGFFTLDFSL
LTSMTAAVVTHLVILVQFQLAGKDPSSSCSCNGTNTTLSPLDSTTPWANLTGE
>CsplGr51n
MVILGETGNPPLLWAHAPLLYLCRVFGVAPFPLARTGSARDGVNCSDYLWIILCGLSGLVSSAYIISIYLRTKYNDY
GSLMDMSFTMSYVSVIYGIIAWNIIALLTKGEGMHGIYYEFHDVDEKMSSLGAAFDFTGRRRSTARESAVIALLTAG
LWVWGQTGFPADLYGGFFGRCAATYASGSNLLIQMNQNALLRDLALRLARVNCEITKLGEAMCDAVEPSGHTGE
NERKHASRAPDEENGADSATFHGAQPWLLERWRAVHLQLSVLGTSMGKLFGPFFLLNMLILGVDGTGILYFIVD
WVIWSSKDLDLFLKIIFGSLLAAYESCGMIFITASCKAISNEADSTGVLVSEVLLKVKDHQARRELRLFSHQLLHTKI
RFTACGFFTLDFSLLTSMTAAVVTHLVILVQFQLAGKDPSSSCSCNGTNTTLSPLDSTTPWANLTGE
>CsplGr51o
MHSTPHAASFDKLERKVTPAARSEPFFWANAPLLRLCRLLGLAPFPIAYEKDGLDGTPVWLLIYCFILELSSLIITVT
VLLAHYSYGEFSKMSDVVLTAWITIALFTSARNRIVFLLRRKDMYSIFLLFKRMDDAFEELQGKVDLAVVRRSSERE
TYFAAIYCIIMVIFFNPYVAVLDNGDAGRALATFFSSTSTFIILLQNYNLLINMLHRYRFLNLDMKESIALIVSETKNLC
TGDEVIRGGDSSQGFTNVLYLWRSIQLDLNKVCTMINDFYGTFILLKGGYEIINITFTFYYILIQLLAVDVGSHRSTIP
TLTSVLLESLPFFFIIYSCQKIIKEADSTGVLVSEVLLKVKDHQARRELRLFSHQLLHTKIRFTACGFFTLDFSLLTSM
TAAVVTHLVILVQFQLAGKDPSSSCSCNGTNTTLSPLDSTTPWANLTGE
>CsplGr51p
MVEKREEEMYSKPNADTCAKREGKISAAARSEPFFWANAPLLRLCRLLGFAPFPIAYEKDGTEEITVWLLIYSFILV
FSSITLMGTALVFQYSYGEYSKMYDAVLTAWITIAYITSARNGIVLLCRRKDIYKICLFFKRIDDACEELVIKVDHVNLR
RSAERQAYFIVTYCLIMVLVHNPYFASIDYGYGLNGSATIISVTSSCVIQLLNYNLLKNVLHRYHFLNMYMREFIAVID
SRSNVCRNGGVDRSEGFTNVLYLWRSINLDLKKLCTKINDFYGSFILLKGGYEIMNITFVLYYTVTQLITIDFFFHSF
QKILKQLTSVSLESTPMIFIICSCHKIVKEADSTGVLVSEVLLKVKDHQARRELRLFSHQLLHTKIRFTACGFFTLDFS
LLTSMTAAVVTHLVILVQFQLAGKDPSSSCSCNGTNTTLSPLDSTTPWANLTGE
>CsplGr51q
MSQCSQRSDNGGSADVKGLKDKGEDFFWDNAPFFRMSRIFGVAPFPLKNEDGPNGSKAPLWLLLYTFLLLTTVT
ALVLIEGVQELMRISGDGLNNAVRSVWVASAHTIIVRNAVMVFTHRSEIYGILLSFVHLDGRLEKLEIKLNHSKIRKS
VERQVSLVTAFIIIQFITMNPLVSTLFSNTPTIFFVKMFYLAICSCMIQLQNNNLITNLLERYRCLNLNIKSLTRRNEAR
RGRMVIMPSDAKRRRGNITRHVILSHALLDGIRLWQSLHLNLYKLSFCVNDEYGSLILLHGIFCIMDVVVVLFFYWD

TITRLNVTLNEIVSMLNCSMLLILEISGVILTIRSCHMTAKEADSTGVLVSEVLLKVKDHQARRELRLFSHQLLHTKIR
FTACGFFTLDFSLLTSMTAAVVTHLVILVQFQLAGKDPSSSCSCNGTNTTLSPLDSTTPWANLTGE
>CsplGr51r
MSKIRGNTRKRKNRPGNINGAKTAKSAEDIVWANAPVLHLSRIFGVAPFALKKENFVRGNRGAWTSRLVMSYTAII
VLCVASKLLYERWKNLRGFFYQEMDSALSKLWIFITSFLTILNILIVVARRSEVCDIYSKYFHLDEKLSDLAVEMDFR
KSRKRAYGQLVWILFGVALLSFTRLPERITFLYNVSISSFFSALFLSISCVLMQFHENNLVKDLAIRFSLLNSEIKGCG
YGRGPESERTLMVPFEAAKKGRCNLNSEPITLLKRINCLKSMHLQLFKIARSINKLFGVLFLLQSLAFFVNVTVVLY
FHLEYMILRESDTRWFLGLLLFLFWAWYENCGVVFIAVACQSVSNEADSTGVLVSEVLLKVKDHQARRELRLFSH
QLLHTKIRFTACGFFTLDFSLLTSMTAAVVTHLVILVQFQLAGKDPSSSCSCNGTNTTLSPLDSTTPWANLTGE
>CsplGr51s
MTLDSRKKDIFWVQAPLLHLSRIFGVAPFPLSHHDFRRDAKVVDCSSFWIISSTAAVFLSSIYFITMFFWYTKDSFY
DTVMDKTMIRYWVALIYSLMLSNVLMLFVRRRDIYDGYTQFVLLDEEITNLRTKVDYDKFRKHVQKQVFLVFILVAF
WVLTSMFGIPFHPEEGGQLLLYLPIHISLANLLIQLNEVNLMKNLGLRFSILNEEIRSFFRPKSSTRRSAIPQMRILE
WGFQMQANDGDDSFELRVGLTMKEYFKRWSSLHLQLWGLGTSLNALLGTFVLVDVISFLLNVTVALYFTVELAIT
DEAIFDKLDKLFTLLVWSAIESYGVVYITGTCHFISQEADSTGVLVSEVLLKVKDHQARRELRLFSHQLLHTKIRFTA
CGFFTLDFSLLTSMTAAVVTHLVILVQFQLAGKDPSSSCSCNGTNTTLSPLDSTTPWANLTGE
>CsplGr51t
MIKKVTFHLEAREIRGFLWSSMPFLKICRILGTAPFPIKNSTAEETPLWQLLYSAALFSSSVCLATQEISMFLAHTPT
FGITTCVILAHYISMEFVFVSNFLVLWTHRSDIYDIFLDFENLDLRFEELSREIEFLKHRKYIGWRMAAFSSGAVVLF
LMRSVGHLSLTASITAFESVYHLGKLYSILAVILIQGHLENLLLCLGDRFSCLNSEIRAGGNSLRVTGKRCRNSEES
MIISDGPSEFPMLAGRIKLWSSLHLDLFNLCSIVNHVFGVNILLQGVHFMVSLLTFCYAFWFFGKSYEHIPENHAKL
VWHVFWVLFEISGVLFTIQSNQSLINEADSTGVLVSEVLLKVKDHQARRELRLFSHQLLHTKIRFTACGFFTLDFSL
LTSMTAAVVTHLVILVQFQLAGKDPSSSCSCNGTNTTLSPLDSTTPWANLTGE
>CsplGr51u
MPTTLDQVVMASFLREGHKVGPYGAPTHRRKEREEFRWDIAWATKPFVRFCSVMGLVPFGPDPLETGTDVNQS
SKLHLSYSVVVFSAFTALFCAVVQSRVSSKKSEVGANIKSTVDMVWWLLSGIVSVASFSMLIARRKELKESLREM
AEIASKLMPKDKNFLNKVRYLLYFEVGTVVSIVILSIWLLHHGGMTRKMPTTYYDVFFQLFSLGATFIMSLQFINQLL
MLYQLLSLVNGEMTSIKSSFKSTNAERRKTGWDDVHMKDFYEKEELSSRIKRHAGYHFSLCHLGPRLNEVYGVL
ALLLAMFVLLDVAYQFYVLINSAIFGATRDELILQLGTILLWSCPKVLTLVLLVAACEFVIDEADSTGVLVSEVLLKVKD
HQARRELRLFSHQLLHTKIRFTACGFFTLDFSLLTSMTAAVVTHLVILVQFQLAGKDPSSSCSCNGTNTTLSPLDST
TPWANLTGE

Odorant Receptors (ORs)

>CsplOrCo
MVKSAKRPSKKKVEPFRDGDKHFKKTMSSMTLDLSVHVKLMFLAGYFLPDFTADLRHSSKFQFSKAIYSIIHMSL
MSLQFTAMAVELAFNFDDVAALTTNAITLLFFIHGPTKVFYFAFFRHKFYKTICAWDTALTEWDGEDELAGKQVSDI
VEKRVAWPSEDNEKPPILIGVDDTNSRVPLFIASSNFFKKDALRKMRSLLVSIMCGSTVAGVFWSIRPFLGLGHYG
RTSTRFIPRDNASITYAENSVFTPNDGLMKDANSTGQWNFIVDATYPWNTSTNPAYILTLLYQLYWLVFCLAQINLL
DLLFCSWLIFACEQIRHLKDILHPLMELSRIPRKKLFSSIGKTQGANSGMNGVFFRPTSLGTENFPRAPCSVLQMS
SLQGQNANRGLIEGGILGHQGGILSPFQGKDVADGPTGALQAIGKGMFRANEADMVIQKATKYWVEKHKHIVRF
VECIGDAYGMALLIHMLTSTITLTLLSYEATKISSIDMHALTVITYLFYTLGQVFLFCIYGNRLIEESTSVMQAAYDSP
WYECTEEAKAFIQIVCQQSQRPMSVSGAKFFTVSLDLFASVLGAVVTYFMVLIQLK
>CsplOr1
MEGHQLKFLKFHLDTFKPFLLFPFQSDGPVSSKLVVIFHFVLTVMNMFRLVGEAMALAVLIDNREEWTRMLPLATA
SLTCAIRMVDVIIKKDRLLSLFTTLDAIFVQCVQLTRTSDEALRVISKGKKATMAYVSVTTLVFLHWLLNPILKGLLNQ
SGRLRRMQPSNVWFPFHYLESPAYEMVYFTLGIGSASSGYILIMTDSLIASLCYYTCFLMRVLGEKIRVLEESKGT
EVVARTDEINWKELDDVMVNCIQLHRAIMRYVKQFENFFSATFATTFLIEAISLCFIAIEAGSSDVSPATAFNLMEIFV
IFAVQLLFFSWSGHAVTQQSLSIHTATAVSWSERTPEHIKWKLRMIMQQSQTPLLIKGGNLIILNMETFKSLIGICISS
FLLLRYLKLNSQA
>CsplOr2FIX
MGKHGIFYLTFLHDVLNVFCRFSDGKKNCRFFFTAVYQLMILISMAFRLAGEIMDFMRNLGRKDNWLEITSLAITSP
GVIFRCSVLMFRGDKFLNLIRKWEELFIRNDAIKPNVERTHKKIKTIERFVKILIIFAAIFTVHWFLHPILIPYFSEEKKR
VLPLRTWYPFDFYESPYYELIYIHQTAMALLTMYSSVALDGSFFMFCYLIKFQASELKANLYRLGETEEQPAMAKG
PQRINRRKQERRLFRCIEHHKAIKRFADEMQNVFGEVLLVELLIYTALICCTTIEGTTAQSADSKSLTWIEYFVVSIQ
QLFLLCWSGNEVTAELASIKMAASASMSGHITNEMKWKLKFMMFHCDEEFRFTGGFFTLSNETFKKFMELFLTN
YLVLKQLKETNSA
>CsplOr3FIX
MGRHGIEHLSFFYDMLNIFCMFPLEKKGCARSIIVPIFHWTVVLALSVRLIGEAMAFYVHIYRMDAWLEITCLLVNTL
SSITRISMLILKRGGILNILRNWEDAFVRNRALRPNIGRTRKKIRVMELYMKIFISLCTLSFLHWFLNPKFASESGDG
QGRKLPLRAWYPYNMYASPYYEITYAYQAVVSFVSMYSMFAVQGTFITLCVLAKFQLMELQSKLVMIGATGEHRL
STEGPRGRRKRRMERKLMGCIEHHRAIKRFVSEIESLFGLIILIAFLQLAVVMCMTTVEATTNMNESRSLAWLEFLL
VSIQQLFLLTWSANEVTIQMKRIHEAVSESLCGYITNDMRWQLKFMSLHSSRVLLTGRGFFVFSINTFTKMIGIVLS
NYVFLRRIKQSRNQ
>CsplOr4FIX
MAKHGVEYLTFLHDLFNIFCLFPPEKKNWMKSILVAFYHWTVVICLIIRFAGVIMGFRMSISKMDIWLEMLSLAANT
GATIIRMIMLLLKKDRILSLIRTWEKLFIFNDSIKPNIELTRKEIRTLEIYAKLLLTPILLTAAYWFVYPIFRAEFSDDQKR
KLFLRTWYPFNIYASPYYELLYIHQTAMALVSMYSLLVVEISFSTFCLLTKFQISELKMKLLRLGKTGSKSLTMGRPR
AVRRRKIEGSFMRIIQHDRVIKRFVNEIENFFSFILIVDVLQLMLLLCVTTIEATVTPLSDSRSLAWIQYFIVCIQVLFIM
CWSANEVTTQLMSIQNAVNEGLCGNLTDGMRGQLKFMILHSNRRFDLSGGGVFVLSIGTFRKILGLAFSNYVVLR
KLKEGKYP

Ionotropic Receptors (IRs)

>CsplIr25aNF
VFVGDSAENSVAERALDAALNYARRNRGLGGGARVDSVRRVVNAGSTLSANDLLDAVCKTYNDSLLEEKPPHVV
IDTTMTGMASETVKYFTSALGLPTVSASYGQQGDIRQWQNLGAEKLKYLLQIMPPGDIVPEVVRSLVQEQNITSA
AILYDNSVVMGHKYKALLQNVPTRHVISPVGGAAKTPQSIRDQLTRLRDLDIANFFVIGRLDTAKAVLDAANTNKLF
GRQFAWHVITKEKGALKCGCSNATVLLVKPEPEQTSRLRLADLKTTYSLTAEPELDAAFYFDLVLRTIVAVNAMLQ
DIDWKTAVQYVTCDDYEETEPPIRINLNLWKYMEEAEISPSYAPISIEGNGQSHMEFSMKLEKVSIVNSMAVSAES
VGSWKAGINSPVNVKNAKSLTNFSSVKVYRVVTVVQHPFIMLNEDGDGPADDQSGKPSGEEGKPAVGQRKYKG
YCIDLLEEIRAIVGFEYEIYEAPDKKFGFMDEQGQWNGMVKELVEKRADIALSSMAVMAERENAVDFTVPYYDQV
GITILMKKPQSETSLFKFLTVLEKDVWLCILAAYFFTSFLMWIFDRWSPYSYQNNKDKYKDDEEKRMFDLKECLWF
CMTSLTPQGGGEAPKNLSGRLVAATWWLFGFIIIASYTANLAAFLTVSRLDTPIESLDDLAKQYKIQYAPVNGSAAM
TYFQRMADIENRFYEIWKEMSLNDSLSDVERAKLAVWDYPVSDKYTKMWQAMEEATLPSSTVEAISRVRGSKSS
SEGFAFLGDATDIRYLTMTNCDLLMVGEEFSRKPYAIAVQQGSPLKDQFNSAILQLLNKRKLEKLKELWWNQNPE
RKVCEKQDEQSEGISVQNIGGVFIVIFVGIGLACLTLAFEYWWYRRKKSDDTDDGGLEMPEVEENSTQRKERRE
NRRKKRRSPGENRRRVAPEILFRHAREFGGIEAASNTASFRFISSILFIFICLFSISSIPADLFTNEFSDGT
>CsplIr8aINT
MKLPNAFVCGLRQAESSLSGVSLSESVIFLGRTDKWTEGELSGVEEDAMQSLSRELTQWNDTRVSAVIDFTWTG
WGRGRKAAISAGVPYLRVESVLASFLRATEAVIAKKEASDAALIFASEEDVDEAVFTMLGRSLLRVLVLNGLGGDTI
PKLLRMRPTPSYFVIFSDSIKLMQLFREMAAASTIVAASEVPAEVWDNSSSSALCLFPRFTVDVEVFSGQTSPANK
VATWLADGPAALGPWGDSGLTVETRAYLSPQRPYLRVGIVEMVPWAYWEDDPVNGGRWTGYCVDFLKTIAERM
EVDYELIAYRDSYFGERRPDGTWDGAIGDLAKGETDMLIASTVMTSEREEVIDFVAPYFEHTGISIVIRKPVRKTSL
FKFMTVLRTEVWLTIVGALTVTGIMIWFLDKYSPYSAQNNKKMYPFPCRKFTLKESFWFALTSFTPQGGGEAPKA
LSSRTLVASYWLFVVLMLATFTANLAAFLTVERMQSPVQSLEQLARQSRIHYTVVKNSTTHQYFKNMKNAEETLY
RVWKELTLNSTGDLSRYRVWDYPIKEQYGHILLAIEQTGPVSSAAEGIRKVLASETGEFAFIHDAAQVTYEISRNC
NLTEVGELFAEQPFAIAVQTGSHLQEEISRRILDLQKDRFFETLTRKYWNKSAKGDCPNVDDTEGITLESLGGVFIA
TLFGLALAMVTLAGEIFYYKRKKMIAQRTLETVGWMKKPPDTKKGKIKNDKGGGLKIKALGQLWKERAAMRHRK
RKVSKCNYIKRGKSS
>CsplIr93aJF
MYPLFSLTLILILSKPLIAEEKSDIIFVTVDVDFSSMWDPEIFPAVRSFAHLASKKYLQGGGLTVTFVDDESFIFRKGE
NELIILLTIARCNDTWGMYHQLNEDNVLQVAVTEPNYILEPDCPRLPIMEALTIPVMDIEEQISQIILDLRSGDVLHWK
EALIIYDDSIGSYLTDRIVKVLSEEIAFGGDKAMPAVSTGIFKLDDSERDYTMQIQIMQFFKNFKASRAHANFIGLLK
PENMVIVLEMAQHMGMVHTQNQWLFYLKDLKTNITNYAPLVNEGGNVAFLYSTANVPPLSGRLVFTLAKSLKQLK
DAEMELFSRVSEEEWEAMKPTARDHRNALLSYLKGSVPCCPGLEWHAAAVESWGLAAAEAPRVFDVASWNPR
VGLVSWDLLFPHVHQGFRRKRILVATFHHPPWNTLYLNDSGDIVDRKGLAFEILDELARTLNFSYTLIHEKTGGNGI
YFDAKKQAFPTFSEKPENHSLLADHTVEVVRRGEALLGAGALVASPRLEALVNVTAPVAIERHAFITSRPQVLSRA
LIFMAPFSADTWLCIALSILVMGPVLYWVHRLSPYYEFHGLRQEGKRITSRAKRKFSIAIRLTAFDKIWKCLWYVYG
ALLQQGGIQVPMADSGRIVIGTWWLVVIVVMTTYCGNLVAFLTFPTVSSTISNLNELKAALMGGDAISGEQVSLGII
DEAVDKGAADSRLRFLSSVAEVHELSAEGGRRAEERRILSRVRRGKHVLVARLSHLMLLAEGDYLSTKRCDFHL
GSEMFMEENLVMIVRQNSPYLKLINEEINKMHHGGLIQKWTSDSLPRKGLCKEKFHERARGDSTEDGGDEGAKN
REVKLDDMQGSFFVLFIGCFLALITISLEYLWHRRNAICEKFNIKPLIF
>CsplIr76b
MQSQCIGYPNEFLWNRNFSFNEALRAFQNWPLSDVKINKTTNKTYGIGIAFRFVELLRKEFGFEYTIIPAKENTAR
GVVNMIIGGLCVDRREEKVPSNIAAKLLCCKQTTQWQKADMAAVFLPMFYTKKLSYSKPLAKEEWVVLLRRPQE
SASGSSLLAPFDSRVWILILLSVSIVGPIMYAIMVVRVRLCRGSSRLSKVFSLTSCVWFVYGALMKQGSTLNPISDS
SRLLFATWWIFITVVTAFYTANLTAFLTLSRFTLPVNGPLDIAIKSFRWIGLSGSPIEQIIKDDIKYSLLQKSINKGLGKF
MNSSDETLMELVRKEGLMFIREKRVVERLLFKDYLKKIEEGVEESQRCTFAITPESFNPLPMAFFFPANSTLPTLF
NPLLTSLVEAGIVDHLMQSDLPHSEICPLNLGNKDRQLRNSDLMTTYKAVATGLLIATVAFAAEQALRLCLRWKNIK
SKRLFKDDMKKAHFKRFSFYSWFMYKWMHAMKNQKKSKSTEVRKGKRKTVSKRKVAVQDSENSVGNLCIHNG
REYIKVYEDTLDGLGITKLIPVRSPSAALFERVHRGGESQLFSRKNDWKGAINCRINCGEYRN
>CsplIr68aJF
MPTQLPYLFRVKKGAVGLPVDGGGERRGRLNGVNTTQGDSDERAGEEENLQRPSGETRTMLRAVSRDGCRLLI
ILMEDGARVGDLLRYGDREREVDTRANYVLLYDRALFLPEVLYLWRKVVNVLFVKAHHGGGAPWFELRTVRFPA
WGEEAEGSGLVDVWSRGAFRLPAPRARPLFSDKTDSLRGKTLGVSTFDHVPSAVRDDVRTGERRASFHGVEIEI
LDTLAEFMDFRVQLSEVEGQWGGGGGSNLTGLAGAVARGEADIALGNLFYAPASLGRFDLSIPYTVQCQTFLTPE

SRLDNAWLTLVLPFQGPSWAALASALLAGGLLFRAVAALHRRARGGSGAGRPFATLGDAVLYTWGMLLQVPVPW
MPAPWPLRALSAAWWAFCLLAAATYRSSMTAALSAPPPKLSFDTLADLATAPAGLTCGGWGDEGRRFFATALDE
PSRRLSERFEALEGERALPTTVAARVAGGHFAYYENEYWLREARAKYLEGPGGEGPPPEAHYGLHVMRECAVN
LPVSVGLATNSPLKARVDRLLRRMVESGLVAKWLADVMAPTLEAERRLQGRGGDQGAPLMDLRRMFGAVLALL
AGYALAGLALAAEVALGRRVGGPKVRPPPRRRPRPAGPRRSGGAARKRAGRRRPLAETIRVTLMGFFK
>CspIIr21aNTE
GLDMHLKVIAMEQPPFTLKRTDGSWTGVEVRFLEILSKPLNFTFHVTTAGKMLAAPTTAWTSTDAAVVVELAAGV
ADLGIGGVHLLPETVEPGAEVRAVFTHSQDCGVFVTQASLALPRHRAIMGPFHWKVWLALTIAYLFGSIPLANILW
TIKEFVRKTIFLTLCLHDAFWDIFGTFTNAFTLSKGISSLKGIMMSTIVGTEGLRLLVAFYWAFTVIVSALYTGSIIAFIT
LPVFPKPYDTGKELLRAGFTWGTLAICKHSRYPSCATFPIIEVYRTQLLGAVFEEPPLIMFMKTMKTINFDAVIMPD
VVGKKRRGPVLAMSNECFVPFRVGFLVKGGPEGNRISEALKMGILQAEQSGLLDKARKDAEQEVGEGEEQEVN
VKGDVGIFLLLGAGFSIAFFALFMEIVVFKIKGKYCPDMTIKSDTEAESDWDVEFSEDDMEEEERESVLLRRLEGS
GRRRFGRPGTASHVRIESQSRISAPDIGMVVYEDSLCEPRAARRAKSAYTYVQ
>CspIIr40aJOI
MLMTSVDEFNIGLATGEGEISYTMPFAVRDIIFGIPTTQITLAYDGSSDSDQLIFTIELFQKSNISLIIYQISTDEEQGKF
FEYMKSAHSTYQTTTNIIISSPPIAELMLAKIQEYNLISRNILYIFHWLRFPVSENFKNTLLEAMRIAVITEHHLGTYRI
YYSQAKSNGENELILVNWWNQNKGLFRFPVLPSAVSTFHDFGGRIFFVPVLHKPPWNFVTYENGTFFTDGGRDD
QLLKLLASKLNFRFEYIDPPERIQGLGVAVNGTFRGVLGLIERREAPFFLGDVTITHDRFQIVDFSSPTLADCGTFAT
HSPRLLNEALALVRPFHWQVWLPVAATAILAGPVLFGIIEASTVWRRQKYKAKASKLLQDCIWFSFGMFLRQSVK
EPSKMHKARLLMILMSIIATYVIGGLYSATLTSLLARPAREKPIRSLFDLEEAIRNKGYQLLVERHSSSLGILQNGTGV
YGKLWESMRGYDVLVSSVEEGMRRVQDVTSMAAIFAGRETLFFDSRRFGAHHFHLGDCLFTRYSAIAMQIGSPF
RENFDQLIIRLFEAGILTKMTRDEYERLREKVDIDHSLRSYSNDEASGMKKNSKSIQGSTGGVEVERKLMKPVNV
KMLQGAFYMLLIGYSISCLVLYGERVIAGRKKYHDGRNNYHLFDKFGFLSSHFFKKLPCWKWKRAHDKLHEISVR
SDFCTNDLCHNCREKSFSSSDEIIYSYKE
>CspIIr75aFIX
MLLSLSCYTIMVLLTLPCIARHNYEKKIGTEIKVIKVYFMTKRISRVTGFVCWGKEDISLLVKELSNSGIQSNFIEAQY
KSDIALRAILRLGDVRERWPLGMLIDTTCHYTQVLLELAFQCKTFDSMHFWLILHDHWVLRNGNERKGNRTECTL
DPYTLRAISRTFSNVQMLINSEVTFAIKRNSTSGYQLCFFDIYRVRINDSLIVTPTSVDKNNPIMDMPQCQPRAVLD
HLVEAMHEITKPLLFTEMHDMKMRHIDTWTKINYILVEHMSMDLNFKVILQQFDSWGYQTNGSFDGLVGALQRQE
LDLGATGMMFKEDRLLVMDYIGETYKFRASILFRQPSLASITNIFLQPFSTGVWLCALLMFAIKVVALTVEMKVEGK
YLQKVRNGKRKDTKSKKDAVHFGEVVMMVVGAACQQGFYSPPSSLSARTTFLALWMASLFLFTSYAANVVALLQ
TPSHLVQSLADLLKAPMDVGIQDVIYNHVYFRETTDSLAMELYHKKIEPNHDRAYYNENDGMKKVKKGLFAFQVE
HTIGYKIISETFTEEEKCGLGNVQLIRTPMLGIPIVRDSPYKEILSQKLHHLREVGVLDRTWKQWIPQKPSSCTNSH
NSASRQFVSIGLEELFSSFSLLRAGAITAVSLLIFECLIHKYVRWRKRHPKFRN
>CspIIr75bFIX
MLSYRLKMNFMLIFVILTIFSLTQGDKTVVHFLSDFCKNRLSRAMAYLCSEKDDLKFAHDIYRSNVYMAVTKLDNPE
GGFRELENSDGNNHILNLDCFESRALLEMLKVLVHKLNTMEWVWNFEVRFLFLTTQSKFKIWTISSVLFLRNEYLK
IANGKHLFRPPNRWVLLTYRLIQDKYLENIIGHLDILIDSDVIIAQRVTETGMNTFALSEVYKPGIHEPLTIKPMGHWN
VNYTFPDVYYPMSLRRKNLNGLTIPGVMVVTKNESLKLLDDLRDKYVDTTSKYNYHLMNHAMDFVNGSVNYSVE
NTWGHNINGTYNGMLGKLQSGKAEIAATALFITAKKLPILEYVSMTTPTKYEQLRFIFRQPRLSSIRNIFILPFSAPV
WALLYGVLLITAVSLFYAVKYENQLEKHDFVKKKKRRPCKYLRASWSDAILLTFGASCQQGFFAEAQGMVGRFVTI
VLYGAVILLHTSYAANIVALIQSSGMLIQTVSDLLYSPMKIGVHDIVYNQHFFQHYSHDPITNQLYQKKIAPPGKKPA
FYGMEEGIEKVRTDLFAFHVERGSGYNLIRETFKKEEICNLHEIPLLQPEGAWVAVKRNSSYSKLIKISFRRIQESG
LQNRGYLRWFAKKPECDGRQSSFVQVGLTDICLSMVALAFGMVSSISILLCELASFKRKKGMAKPGLYKKKKVNK
FAYKW
>CspIIr75cNTE
TDMNTLNETSTTYNMYELFKVGYKTPLKWVSIGNWSSDRRSVTHPETVDKLDSLDHKYDDTSTKMNYHLLRHV
YDVINASTKFVTVDSWGYEENGTYDGMVGQVLRGEVEIGGTPLLINRERVEILEYLGITSHSKFRFIFRQPRLPSV
GNIFLLPFGTSVWISSFAMLVLTAIFLYVTIKMEKVFYENNMDLALELAKHGHHSLMKDGQTKGWVRKMAKVKFIQ
DSWSDITLLTLGALCQQGFPSETRGAAGRVVTIFLFMTIIILYTSYAASIVVLLQLPGATIKTLRDLYESPIKLGLDEIIA
QRHIFQEMTEPLKMKVFQKIAPPGRPPAFYPMEEGIAKIRNGLFAFHVELGSGYEVIFETFTEDEMCSLKEINIMPK
TSVWLVMKKNAPFEKVFIYGLRKVLEFGLSNREETRWVSKKPECNKRHLNFEGVGLIDIRHALLMYGVGFSLSLIF
LVSEIVIHKRQKRKNFQKKETESKDRT
>CspIIr101INT
MPLDFRRERKTTKCSDTVATPVANPMIGRGCVLPLSLLILSATAVEIAEDERLLDRVLDQIYSLHFSSLKCLVVVDSR
QELGWLPPLPEAPNIPTPLISVSLDSEDVSALSQGPLSQDAAASILSEDADADWAPPPPSSSTPALRALLAARRAS

CDSYVVYLPATAPDTGLSLVKDGPRSGLLHGTARLLLVHDSAGSTDLERALPPSHTLTRVAAVEVGPAGLSVRKRL
PDGRWEEWRAGEALFGSASVTRDLGGRRLVMSTFPYPPFSFVKYGVERVPMPGSVECGEDGDVPLSSLEGTE
VSSVVGVGEREYVIPRYGGVLADVSNNFADLGFAGFYQDAREAPLVDYSAPFTSAGLSFLVPRPTSPAVPRWMS
LVKPFNPWSWAAVGGTLAAAALIVHLLASVATRVLPPPAPAAYARYVHLPDCLLHVASVLAQVGVPMPTGNEGRW
PLQWLLPGLNLITYFYSGCLAAYLTLPSPEAPIDTLSELREAGLPWGARTSYWSLLLLHNWENPDAQVLGRRFRLI
LDPMALRRGLLSAEMAAAVQTVQGSYVTNAEYLDARGFSQVHLMKETAFEGYVSILLPKGSPLTRPSDEIIGRLLA
AGLVQRWERDLXDPVVLSLEHILGAFFLLIGGLITATATFAAELLAARHPTA
>CspIIr102NTE
ASLTNHFRGRYKWLILTSGKKFEFLGALLQELDANVDSDVTLAVANPEGTIEMYAVVSYLNSKNCLTFFTQFFFIPV
KINWERSIQHNHTYVVFSLSVCMSPRKCEVNTLRNVMPKFHVCTKCNVKFQLMEIYILQSTVHQSMIGNWVPKYL
DGTESPSTNTSLATPLLEFGSFTLNNQVGKLFRRSDLNGFNLRVVTVNVSSFYTLVLLDPVSQLPKISGGYFGEIF
NMLSEAMNFTHSINVLEGYAYGVILSISYNVPNWSGVVGELQHKHADVTACEVSLMPQRHIVMDYSMPLQIIRRRI
YIHRPMRSGVGAGGSKSETTWYKGGGFVRPFAMQVWICITASLLLFLAASRLHAFYTFPSKDKAYDHDGIVEEAIL
IKGRKKIKNISQLEENLTSPPTLSSDLFRFTATLVQQGLPDDPDPDESKGEVNSLQSRRLILWTASVTYLIVYTSYGA
KLASILASESEPQPLFTDLKGLLQIPNWKLGFLEKGLGRVSFENAEPDSVMGRLWAEKIENEPEVLVPNIETGLQR
VLKEEYAFFGFHTGVQAVLVESLSKKDTEEPVFTTSEFCRLMELPNDYLKGGIAFGFQKESPFRPYFDNWLLLIRS
CGLLDRLAKVWLPEALPCLEDQIVPTVGLIDIAPALSALLFGLVLSVSILLFELLYDAYKRRPGHSFRFQS
>CspIIr103NI
SSDNREFRSRYNWLILTPVSKQNDLEELFQQIGVYTDSNVNFGIIYSSHVVKGLVLTFLSTLLNQKMEIELPSSKAG
QKKHLIFLVMVSSEVQLKITNLNYDLQIFRYSLKYIPGYSYGKIKELGNNWTGVVGHLASKRADIALCGISESKWRS
SVIDLTAPLIFQRNSPKNSSLGILWVQKIQEKPSNIFKYPKIGLLKVSQNQKFVYLGDSLECRLILSGMKKNQGEVF
KLHSYNEGEKMLPRNVGCKIAELPGDVLKIGSSFGLQKYSPIKDIFNYHLMKLWSTGVLQILRKRFIEPPGKLCDA
PSVFQPASLRDTFFSLILFGLGILFSLFILVTEFTVFKMKKRSQKTKRKNKKEISYQE
>CspIIr104NTE
YSVYSSEEDKLIRNHSKCCQLNEGKIECTASDCHTWNDMVGQLMKGEADVVATSLTPTTGRLQTIHFSSPILFTS
EQLFIRLSSKPPISYNFYLQPFSSHMWLMIVFIFMCILPMMHSMIFWLSPSHFYLNNPNDVQGGLEYHIKSDFPMS
SKIVDTLSHWLTESFGIFCQQGIQFVPQSQAVRLSLYVTMLSSSVMYNVYSGSLTSFLAVDRQIKPQYDTMENLLQ
SVEPSGLSIGVLENSSLISVLSENSSGAYKELWSTISDSKQGIVPTRFEGFHRACVENFVFFTMKASYEGYRKFLQ
PCELYGIPESNLNTYVGISMAKGYQYAEILDDLLRMNEAGMLQRQRLKWWPTYYPEEYTYAAAQKDAVETGTSD
PIMKITLGHITFAIKLLFGGMCLSIILLLCEKLSA
>CspIIr105
MVEFRTASRGAHGKTASPWRGVGPAAMAFIALALRPATAALAPPGPHSVQSDLDTFLEDLVTRYSGDGFRFVFH
VDKRPERPPPLSAYFAPGVEVGRLVVSHPEGAAGARRPVGYQFIHVVLLQKPSGFLDFVAGKGSVRPPDRVLFV
LSREALEEEEARGQGAFWRAPAGTRIRRFASTAFAVTGGRGLATYDVCHYCGPLSGALRPTGAWGPALPRLDVF
PDNWNDLRGHQLRVLFVPFPPIIWCMQSAELRTGCRAGCGERVCVGAASGPEGMLLQEASRRLNFSVHLVDFG
WAEEGWSEDAAWLHQQQDGGMTNASPWDAMVGAMASPAPPGDLAVGDISITASRTQQVYFTTVFNREPHTFA
FLRSEGRAAGVSGPFSAPLWFCLAAAWAATSLILAALQASCRFLPEGLWVTGAMLLGQGAPTTPPALRGSRPAR
PLLLCWCWLCFVLASLYESKLVSSLVNPAAPEEPSTLKDLLVHRYEMLTPRSSLFAVRGLLGSTDAVYRAAGRRM
RVLPTVEDCVERLVRASRRGPLVALIAEHSFLRWGLPLMIPEGEQGLASSLRLGSAEILAYGQAWALRRDAMALE
PALSRALTSAIASGLPRYWLDRSLKNGREAVGQVRRGGGPHPGAAQGLPLQLFSASLCVWAGGLAAATAVLLAE
VARPPSPRAGRERPAKPLGVKSRNASAPDLTQKQRHVNAPECGAFD
>CspIIr106
MNGYVSGSLTMGIVILHFLAFSILQPSVNASIAPDKMSLTEIDTDSTNLKYFLGGLLAFYSHERNMFIFHVEFRSKET
RPNFIDYEKLFPSLFSPRDAIEMMVIDYSNYQIPMSRPTGLIVINVILLPKPHRFHRFFDAAKNVGPYDKAVFLLTKK
YLNQQDSMGDLAFWRKYVSLSHFTNVVFLVPTKLGVEVYDTCYYCGTQVGRLRFSGVWKVMQSTGESQRKIFS
EIFQDNFKDFNGHELSVLFLKFPPSIWCERMKVAPRKKLNSTESICMGKLSGTEGSSLSLISRSRNFTTRVTDFRS
RPESEATVGRCEWITDLLLKQLLANECDMVIGAISITSNRAKYVHFSQLFAREPVNFVYIHRAGTATGLAGPFSTNV
WISLLVSWLTTAFIFSAIRKTALGEVLWATGALLLDQGSSMPPHLLRNRATRLFLLCWSFSSFILFSIYQSNLVSSLIN
PVPSHEPSSLQELLNQNFKLVASKRSMFALRALSESTDPEYRIAAERTLVYNTLEECTELLIRSWHEKSYTALMGE
DSELHWGLAGQFEQQDQGYLGLQLGKMEILATGHAWALENLALMKTLDSALQSIVASGLPGYWLRHQSRINKGP
MPYRYDNDRQIARSFVPLPLQLFSVSLSIWAGGNIVAILVFGMEYLIFHNGKKIILTFSPGSIGL
>CspIIr107
MPKLKRIFPILLFIFSLSTAHTKFSMLTTDEGTIHVKTFLKKAISCFINEGHRLIFHVEKTDSKDITTDFIDFELLFPGIF
SQGDSVERLVIDYTNYPTSSRRPSGYRYINIVLLLRPHKFQYFIECSKNVRPHDKVLFVVTKAFLQRQESLRENAF
WKRYAELSRFTNVAFIAPTKSDVELYDTCYYCGSKSRILRFIGISRGNHAIKERCGFFAQLFPNNFKDFNGHELIVP
YLSLPPMLECNMLEEVPRPSSNGKIKVCLSKPQGPEGNTLTEISRSLNFTTLLVDFMSTEESKAVLRADLGMWDIL

IARISDGLGDMTLGAISVTPERTKLVHFTQIFNREPVNFVYFHKEGAATGLTGPFSPLLWVCLAVAWVITTALIYAFA
KCGGLHTLAGGMWATGALLLGQGSSIPSYLQRNRAARLLLLSWCWFSFVLISIYDSKLVSSLVNLAPSRNPSSLE
DLLLQKFEIVAAKSSIFAIRGLLESSDPVYRTTAERIRVFGTLEECVEVMMRAWQHGPPTAILGETSGLRWGLERP
FAEGGYEDATPHLRMGDAEILATGHAWVLRNRVFVPAFDRALGEIAASGLPRFWLDRQLRGRSGPVPYGVAVGR
QEPRSFIALPLQLFFVSLSIWAGGLLVSVIAFGIEFFSVMHGAKKCLPAH
>CspIIr108
MPTKLEILNFFAFILFFSSFVECREGKIFRPILSNLSTYNQCRSVREYFPLGCVNNQENENKFIGNNSKKLLKNIFLE
RSSEEPSILLHPIIYSIVDMIQRHFKNCNNIAVVTDIATKEVMEALLAQIQQDLTLAVSVYGYPCISECRHINSTSQCQ
KESVEAGNWKFELVLFLTMDPRNYQSLFAIFSQNPSWSISIPVIVGIATELPNKYASGIVNDILHEIWKYWIFKVVVV
VKTKGIPYATVYNIKPYSGGNSCGRDINEDTYLGYWKTEHPLKLGIFSRSSKFMDLHGCPMNIIAINFPPSVIFPTP
TTNPENLTKLSGLEGKLLEAIASVLNFRPHFHLPKRGISHGFHLPNGTFTGILGDLAAGDGDVGMAGILTTPTRLAV
VDSGVPHSRDCFTWAVPVKEEEAGLLFVLISIASKEVWLGLLLVLATSSVAAWILTALSPPIQRGRRGRALGQSFIV
MMSGFLGVTFHQPPSFAPVRILYWVICVVGVTVSTVFQSQLVSVLTAPGTPSWPRNTRELLESNQKLGGSAYLLQ
VIADLNHPSGSSVARNFVVEKEGENFLKEGAWAVPGAKQNLIHRVSESKILRVQSECLFYFHRSLIFRKRFPFLDD
FNTITSFAAEGGLILKWDRDIDKSGLNKGIHSKHQDQRKVITVHELLAAFVILLIGLSIAIVSFAFECFIKLK
>CspIIr109
MDKNFLYKKVMTVGILYLLRFLTLTECSVPLPPHNTRQNDNGIEDSFKNYKDLSSEVRKRESGIQSGIKYVFFKHN
FNSPTNSNILLSFLADMTEEYYKSKMKIAIVTDSSLNDATELLIHIQQKFKSSVSIFRYPWKELCVEENRMKFPVNW
HATCDERKFQHVLFLVSQPGKFGNLLSTISKNPSWCISAPVIVVITASLKSKVLEGVIRSTLSETWKYWIKNVAVFV
KKRGLPSVYTIRPYSGGKACGKDPGEPIFLGDWESIQSASQKFELFSNLSKFRELHGCAVDIVTHQEPPMVIIPRG
KKLENLSKVGGIEGRLLQTIATKLNFRPHFRSPRNGNSSGLRFSNGSYNGVIGDLADRVADVGMGGFLVTNKRLV
VVDSGFSHSSECITWAVPMRSKDALLGNTFLTVASEAFWAVIFVMMVGSSAVFWVLSKWSPFSEDKGEKNFGGA
CVTITAGFVGMSIKRSIYSPSSRLLHCCICLTGFALSTALQSQFVSVLSKPDSLYWPKNALELLKSDQKFGGSAYFL
QIITDINHPLLGVVKQRYVVEGPKDNFLNDGDWAMPGDRKNLLYLTAGSKTLRIQSQCVFHYPSCLLFRKTFPLLS
NFDSITTFAVEAGLVNRWSSDIFFRFRRKKLLLEHKDKKISLSFNDILPVFDTLLINLSIAFIMFIFEIIYFHLNIFR
>CspIIr110
MFVHGMPSPTGARFLAIGVIILRASALSSFPEPIASLSPSIGGGGDGRVDDDVAEPNSFDSSIVDCVVEILCAHRSG
PPTVAVVFGDEGGPLLSRLVPALQRPPVETPVLLLAFGTRTRGDPVTSAPSSHPKNMSHAVFLPSPKAPFHRLFE
QATLDPRWSPTTMHLVLASEAGESDEGAEVHAVLSLAWRHWVANIAVAVPAKEADEQLDPRISIVTHFPYSSGGI
CGRDSTKIQEVAVWQKGRFTLGSPKGVYPEKFGDLHGCVVKATVFDAPPACIPKVGRETTLITQSGKGDQEDEV
VHVGGFHGLLLAEAAKRMNFTPRINQFFPERYGNSSTPFWPLFPPLIEHQVDMLLCEVVLVPKRYYLLGVGFHH
QWACTTFAVPISHPRNSWRLIGLATPPAWLALLASVILSCTASWTIDKLQPYPLPVHQHHSSKVVTDALVTFAGDV
LLRPPTTPSSRVLFPAVSLGGLITTTAIRAHLLSLLSAPDTTTWMPLNLEEMVGSGMRVGGPMEFKFLTKDMKNEA
WETVLKHYEIHDEGTEALDIDGVAVAGMGRNLLYRLSRKRRDKGVLKECPLPLPTVIFLNKGSPFQMELKKIDRGL
VSGGIITKWMHDHTSMKATFRAKSVKKGEKRKINVSEMKGVFIFYSIGLTISLVVFIAEVSMVKLNSYEVI

Odorant binding proteins (OBPs)

Calopteryx splendens

>CsplOBP1F
MSASQSGMLVFWVALAVLEVVSSQHYPEQQHQDLSHTNQRCAQNPASSQKLEKVIDECQDEIKLAILQEALESL
QESSPAALKRRERRAIDFSSDEKTIAGCLLQCVYRKVGAADAVGFPDPEGLVKFYVDGVEDRGYLGATKIAVHLC
TRQASLKRGFAGHGAQACGEAFDVFQCVTERLAAYCES
>CsplOPB2C
MQHYVSLVCAALVMLLGCTDGMPEDPMLKIKEKYSTCKTRLNPPDDVVSAMMSTGLLNDESSEAGTCFVQCVM
EELGIVKNGVFNTEDKLKEVKETFKDFKKPDGSTVDVEEITKGITECAAAGVGDTACAKNYRIWVCIKSKRQK
>CsplOBP3N
KELEISKKCLETFQISQESEEYFNKEALLKNEKDATELCYASCVIEEYFGKENGGIKKEGILNLLLTISDSSPTPGEV
TTVTTKWNPIIDSCLKEGGTTTCEKNYNLWKCILKNMQG
>CsplOPB4N
LSINEESAENIEKLMDDCRIKFPISDETVKVLHSMGSLKDETDETAMCYVNCVMEAMGIVREGIFQVDEQMKIAES
LLKGVKKADGLNYDINAVKKDIADCSKLEGNGVCRTTYRITQCIETKRKEAGIVAPN

Ischnura elegans

>IeleOBP1
MSSCRLEFAFAVVLVLLEVVLSQHYPEQQHQDISHTNQKCAQNSASSQKLEKVIDECQDEIKLAILQEALESLQET
SPSALKRRERRAIDFSNDEKTIAGCLLQCVYRKVGAADATGFPDPDGLVKFYVDGVEDRGYQGATKIAVHLCTRQ
ASLKRSFNIAAVQQESTDRQRRRLDAEVVSTAGINKPIPDDQAEGIGAQACEEAFDVFQCVTERLAAYCES
>IeleOBP2
MQLCAYLVVAAVVLLYSPTEGLPQRIVDKMESAIKECQGRFKISADDLETFRTSGMLKDEKAPDGTCFVQCVMEE
MGMVKDGIFSTERKMQVSEEAFKDFKTPDGAVIDIEKMRNGVTECAAAATGDTTCAKNYSIWVCLGAKRQEVGL
>IeleOBP3
MVKVLSVAALVILAQFAAGEPDRDEISKKCLAKFPIPKDSQEYFLREMLIKNEKDASELCFASCILEEVFGKENEGL
KKEGVMEVLLKYPESSESEDVEARKKNLKPIIESCLNEGGSTTCEKNYNAWKCMEKKLDQGSEESKS
>IeleOBP4
MMKISTIFIAVAACLSPLQVLSAAQGPEKDLDMIIKNCRSTFAISDEAVSFLHSTGSLKDETDDKAMCYFNCVMEG
MGLVKDGAFQVSEQLKVTEKILNGKKKPDGTMYDLVGLKTDIENCSKLQGNNVCHTTYVIVRCIEKKQSDLGITKP
NN

Coenagrion puella

>CpueOBP1
MSSCRNTFSFVVILVILEVALSQHYPEQQHQDISHTNQKCAQNAASSQKLEKVIDECQDEIKLAILQEALESLQETS
PGALKRRERRAVDFSNDEKTIAGCLLQCVYRKVGAADAVGFPDPEGLVKFYVDGVEDRGYLGATKIAVHLCTRQ
ASLKRSINIPGVQQENTDRQRRRLDAEVVSTAGINRPIPDDQAEGIGAQACDEAFDVFQCVTERLASYCES
>CpueOBP2
MQLYASFVIAALALMLSPTDGMPQRLIEKMDAATDVCISRFKISSDDIETFKASAKLKDENSSEGTCFVQCVMEEL
GMIKDGVFSIEQKIALAKDIFKDYKLPDGTAVDYEKMRKGLAECVSAATGESTCAKNYSIWTCMGNKRGEVGL
>CpueOBP3
MIKFITIFIVAAAYLSPMVMSEAQMDEKDLDTIVKNCKTNFPITDETISFLHATGSLKDETDDKAMCYFNCVMEGMG
VVKDGVFQLNEQVKAAEKLLDRVKKPDGTMHDISAVKMDMENCSKLQGENKCRTTYAIVRCIEKKRRELGITKPN
N
>CpueOBP4
MVNTLPLAALLLILAQLIAGEIDRDEEIMKKCVVKFPIPKESQDYFVEEMMIKNEKDVNEQCFANCVLVEAFGMENE

ELKKEGILEVLTSYIDSSDKEDIELQKRTLKSIIESCNSEGGTTTCEKNYNAWKCITKNIDRNKDQLKIKS
